# Antimicrobial Trioxacarcin 1,2-Dihydroxyanthraquinones from the Bacterium *Streptomyces* sp. 127Q Isolated from the Stingless Bee *Tetragonula carbonaria*

**DOI:** 10.3390/biom16081159

**Published:** 2026-08-10

**Authors:** Anastasiia Filimonova, Dieter Spiteller

**Affiliations:** 1 Chemical Ecology, Department of Biology, University of Konstanz, 78457 Konstanz, Germany; anastasiia.filimonova@uni-konstanz.de; 2 Konstanz Research School Chemical Biology, University of Konstanz, 78457 Konstanz, Germany

**Keywords:** anthraquinone, bee pathogens, epoxide, gutingimycin, human pathogens, *Lysinibacillus sphaericus*, nicotinic acid, polyketide, *Neisseria gonorrhoeae*, *Staphylococcus aureus*

## Abstract

In contrast to honeybees, the Australian stingless bee *Tetragonula carbonaria* appears to be robust against microbial pathogens. *Streptomyces* sp. 127Q, isolated from *T. carbonaria*, inhibited the growth of *Lysinibacillus sphaericus*, which is the only microorganism reported to affect *T. carbonaria*. The antimicrobials were purified from *Streptomyces* sp. 127Q by bioassay-guided isolation using ethyl acetate extraction and Diaion HP20 chromatography, followed by reverse phase HPLC fractionation. The antimicrobial compounds were identified by high-resolution mass spectrometry, UV-Vis-spectroscopy, nuclear magnetic resonance spectroscopy, and genome mining as new members of the trioxacarcin/gutingimycin family having a 1,2-dihydroxyanthraquinone aromatic core structure. A closely related gutingimycin with a 1,2-dihydroxyanthraquinone moiety was previously observed in a crystallisation experiment of trioxacarcin A with an oligonucleotide. *Streptomyces* sp. 127Q produces a wide range of trioxacarcins/gutingimycins. At the onset of trioxacarcins production, *Streptomyces* sp. 127Q appeared to rapidly convert trioxacarcin epoxides with water, guanine, and nicotinic acid. The 1,2-dihydroxyanthraquinone trioxacarcins, trioxacarcin 692 and trioxacarcin 780, inhibited *L. sphaericus* with minimal inhibitory concentrations (MICs) of 37.5 μM and 75 μM, respectively. The MIC against *Escherichia coli* and *Staphylococcus aureus* was 75 μM and 150 μM, respectively. In the photoantimicrobial screening, the MIC for trioxacarcin 692 against *E. coli* and *S. aureus* decreased by 8-fold, and for trioxacarcin 780 by 4-fold. Following light pretreatment, trioxacarcin 692 (9.4 μM) inhibited *E. coli* and *S. aureus* at concentrations comparable to those of the established antibiotics ciprofloxacin and vancomycin. *Neisseria gonorrhoeae* was only inhibited at an MIC of ca. 18.8 μM after light preincubation. The 1,2-dihydroxyanthraquinone trioxacarcins constitute powerful antimicrobial compounds belonging to the trioxacarcin/gutingimycin family.

## 1. Introduction

Microorganisms are enormously widespread across the Earth and are essential for the functioning of its ecosystems [1,2,3]. They are so successful because of their enormous diversity, their metabolic capabilities, and their short generation cycles, which enable them to adapt to diverse habitats and challenges [3,4]. The most obvious microorganisms to humans are those that cause disease [5] or affect agriculture [6]. Moreover, microorganisms are a rich source of secondary metabolites that are indispensable to modern medicine, for example, in the form of antibiotics, immunosuppressants, and anticancer drugs [7]. A major problem is the emergence of antimicrobial-resistant pathogens, which render established antibiotics ineffective. These pathogens already cause about five million deaths worldwide each year, and will lead to a drastic increase in patient mortality in the near future [8]. The WHO recognizes this as a severe future health problem [9]. Thus, there is an urgent need for new antibiotics, among other measures [5,9]. A common strategy for discovering bioactive compounds is to exploit the rich secondary metabolites from microorganisms, although the rediscovery of established compounds hinders the identification of new antibiotic leads. However, high-resolution mass spectrometry allows the fast dereplication of known compounds [7,10]. Various strategies are employed to identify promising secondary metabolites. These include genome mining for unknown biosynthetic gene clusters, metagenome analyses, variations of cultivation conditions, and the modification and optimization of established antibiotics using biotechnological and semisynthetic approaches [7,10,11,12]. Additionally, innovative isolation techniques [13] and screening techniques [14] can facilitate access to unknown microorganisms and their secondary metabolites. Furthermore, secondary metabolites from microorganisms in lesser-investigated environments are being investigated [15]. Moreover, secondary metabolite production can be triggered by the environmental context and interactions of microorganisms with other organisms, and this can be used to identify novel natural products [7,16,17,18,19]. Microorganisms associated with higher organisms constitute a promising source for the discovery of bioactive natural products. In recent decades, the chemistry of microbial symbionts has gained increasing attention, not only as a source of secondary metabolites, but also to understand the chemical basis of the mutual benefits between microbial symbionts and their hosts. This changed our view of the significance and impact of microorganisms on higher organisms [17], such as humans [20], plants [21], marine organisms [22], and insects [23]. Insects often form partnerships with diverse microorganisms because these microorganisms provide vitamins, make nutrients available, influence the insects’ life cycle, and protect them from pathogens [23].

Our understanding of the chemical details of microbial symbiotic interactions has increased significantly in recent decades, particularly with regard to leaf-cutting ants [24,25,26,27,28,29,30], termites [31,32,33], *Pederus* beetles [34], bark beetles [35], *Ambrosia* beetles [36,37], or the beewolf [38]. Recently, microorganisms associated with stingless bee species have gained attention, demonstrating that they could provide antimicrobial compounds against bee pathogens, as well as influencing the life cycle of the stingless bees [39,40,41]. These microorganisms may contribute to the remarkable resilience of stingless bees against common bee diseases. In contrast, honeybees, which are key pollinators of agricultural crops, and thus are essential for securing human nutrition, suffer from many diseases. Honeybees are seriously affected by *Varroa* mite infestations [42], viruses [43], and microbial pathogens [44,45]. Moreover, human impact destroys their habitats, and the use of pesticides has an adverse effect on honeybee fitness. These factors have led to a worrying decline in bee numbers, known as colony collapse disorder [46].

In this respect, the Australian stingless bee, *Tetragonula carbonaria,* is of great interest, as it appears to be unaffected by the diseases that affect honeybees [44,45,47]. The only known reported pathogen of *T. carbonaria* is *Lysinibacillus sphaericus* [47]. We therefore reasoned whether microorganisms associated with *T. carbonaria* might contribute to its resilience. These microorganisms could potentially help to protect honeybees as biocontrol organisms, or their secondary metabolites could become useful in combatting bee diseases. Furthermore, antimicrobial metabolites from these microorganisms could provide novel antimicrobials or lead compounds against human pathogens. We previously isolated microorganisms associated with *T. carbonaria* from one beehive in a garden in Brisbane, Australia. Because the microorganisms were isolated from *T. carbonaria* bees of just one beehive, we cannot currently deduce whether our microbial isolates are widespread among *T. carbonaria*. Moreover, we cannot determine whether the isolates constitute permanent mutualistic symbionts or bacteria that were simply found in association with *T. carbonaria* during our snapshot sampling. Future experiments are needed to address the potential biological role of the isolated microorganisms from *T. carbonaria.* In order to begin studying the microorganisms associated with *T. carbonaria*, we identified them by their 16S rDNA, screened them for antimicrobial potential, and sequenced the genomes of some selected isolates. This resulted in the description of a new species of the little-studied genus *Rosenbergiella*, *Rosenbergiella meliponini* D21B [48], which had previously only been found in flowers, the food collection range of bees [49,50,51]. Among the microbial isolates from *T. carbonaria*, several *Streptomyces* strains exhibited pronounced antimicrobial activity against the bee pathogens *L. sphaericus* and *Paenibacillus alvei*. From these strains, *Streptomyces* sp. 127Q was selected in order to identify its antimicrobial secondary metabolites. Here, we present the bioassay-guided isolation and identification of 1,2-dihydroxyanthraquinones belonging to the trioxacarcin/gutingimycin family from *Streptomyces* sp. 127Q [52,53,54,55]. Moreover, we evaluated the antimicrobial potential of these 1,2-dihydroxyanthraquinones against selected bee and human pathogens.

## 2. Materials and Methods

Chemicals: Acetic acid and media ingredients were obtained from Carl Roth (Karlsruhe, Germany). MOPS buffer was from Bld Pharm (Reinbek, Germany). HPLC-grade methanol, acetonitrile and dichloromethane were obtained from VWR (Darmstadt, Germany). Double-distilled water was prepared using a quartz glass distillation apparatus. Deuterated methanol (CD_3_OD, 99.95%), chloroform (CD_3_Cl, 99.95%), and DCl (ca. 4% DCl in D_2_O 99.5%) were purchased from Deutero GmbH (Kastellaun, Germany). The Diaion HP20 resin was from ThermoFisher Scientific (Dreieich, Germany). Reversed phase Polygoprep C8 resin (50–60 µm, Macherey-Nagel, Düren, Germany) and the Nucleodur HPLC columns were bought from Macherey Nagel (Düren, Germany). The Synergi polar RP column was obtained from Phenomenex (Aschaffenburg, Germany). Vancomycin and ciprofloxacin were from Sigma Aldrich (Taufkirchen, Germany).

Instruments: A Waters Acquity UPLC System (Waters GmbH, Eschborn, Germany), equipped with a Surveyor Plus PDA detector (Thermo Fisher, San Jose, CA, USA), and connected to an LTQ mass spectrometer (Thermo Fisher, San Jose, CA, USA), fitted with a heated electrospray ionisation source (HESI II) operated in positive ionisation mode, was used for standard LC-UV-Vis-MS analysis. An LTQ Orbitrap XL mass spectrometer (Thermo Fisher, Bremen, Germany), with a heated electrospray ionization source (HESI II) operated in positive ionization mode and hyphenated to a Ultimate 3000 UPLC system (Dionex Softron GmbH, Germering, Germany), was used for liquid chromatography–high-resolution mass spectrometry (LC-ESI-HR-MS). Mass spectra were recorded at a resolution setting of 100,000, using the lock mass function of the freshly calibrated instrument to determine the molecular compositions.

Nuclear magnetic resonance (NMR) spectra were recorded using a Bruker Avance Neo 800 MHz spectrometer (Bruker, Rheinstetten, Germany) (^1^H NMR 800 MHz, ^13^C NMR 201 MHz), equipped with a TCI-H/C/N triple resonance cryoprobe with Z-gradient. The calibration of the NMR spectra was performed using residual CHCl_3_ (^1^H 7.26 ppm, ^13^C 77.16) or CH_3_OH signals of the deuterated solvents (^1^H 3.31 ppm, ^13^C 49.00) [56].

A Büchi Sepacore medium-pressure liquid chromatography system with a fraction collector (MPLC, Büchi, Essen, Germany), fitted with a Macherey Nagel reversed-phase Polygoprep C8 column (15 cm × 2 cm i.d., 50–60 µm particle size), was used for the preparative scale fractionation of crude samples.

The samples were then further purified using an Agilent 1100 HPLC system (Waldbronn, Germany), which was connected to a Gilson 206 fraction collector (Gilson, Berlin, Germany). For semipreparative separations, a Nucleodur Sphinx RP column (250 × 4.6 mm, 5 µm, Macherey-Nagel, Düren, Germany) or a Nucleodur Isis column (250 × 4.6 mm, 5 µm, Macherey Nagel) was used. For routine LC-MS measurements, a Nucleodur C8 Gravity column (250 × 2 mm, 5 µm) was employed. The optical rotation was determined using Jasco 2000 polarimeter (Jasco GmbH Deutschland, Pfungstadt, Germany).

Microorganisms: *Streptomyces* sp. 127Q was isolated in 2017 by Dr. Anthony Farlow and Dr. Darshani Rupasinghe from a domesticated hive of the stingless bee, *Tetragonula carbonaria*, located in Brisbane, Australia (27°33′ S, 152°56′ E) [48]. *Lysinibacillus sphaericus* DSM1867 and *Paenibacillus alvei* DSMZ 29 were obtained from the German Collection of Microorganisms and Cell Cultures GmbH (Braunschweig, Germany). Clinical isolates of the human pathogens *Staphylococcus aureus* 33P, *Staphylococcus epidermidis* P417, *Escherichia coli* EPEG, *Klebsiella pneumoniae* 234P, *Moraxella catarrhalis* 49P, and *Neisseria gonorrhoeae* 9P were kindly provided by Prof. Dr. Christof Hauck, University of Konstanz.

Cultivation conditions: *Streptomyces* sp. 127Q was re-streaked from a cryostock stored at −72 °C onto three plates of SFM agar medium [57] (20 g/L soya flour, 20 g/L mannitol, 20 g/L agar) using a flamed sterilized inoculating loop, and grown for 7 d. The grown mycelium was then used to prepare a spore suspension in 20 mL of R5A medium [57] (103 g/L sucrose, 0.25 g/L K_2_SO_4_, 10.12 g/L MgCl_2_·6 H_2_O, 10 g/L glucose, 0.1 g/L casamino acids, 5 g/L yeast extract, 21 g/L MOPS buffer, 2 mL trace element solution—40 mg/L ZnCl_2_, 200 mg/L FeCl_3_·6 H_2_O, 10 mg/L CuCl_2_·2 H_2_O, 10 mg/L MnCl_2_·4 H_2_O, 10 mg/L Na_2_B_4_O_7_·10 H_2_O, 10 mg/L (Mn_4_)_6_Mo_7_O_24_·4 H_2_O; pH adjusted to 6.8). A spore suspension (2.5 mL) was used to inoculate eight precultures, each containing 100 mL of R5A medium in a 500 mL Erlenmeyer flask with a stainless-steel spring. The *Streptomyces* sp. 127Q seed cultures were grown for 3 d at 28 °C and 120 rpm. Subsequently, 1 L Erlenmeyer flasks with stainless steel springs, and containing 300 mL of R5A medium, were inoculated with 15 mL of the pre-culture, and cultivated for 21 d at 28 °C and 120 rpm.

To investigate the influence of media on secondary metabolite production, additional media were tested. The SYG medium [58] was used for trioxacarcins production in *S. bottropensis* DO-45 (60 g/L soluble starch, 10 g/L glucose, 10 g/L yeast extract, 3 g/L NaCl, 1 g/L MgSO_4_·5 H_2_O, 1 g/L KH_2_PO_4_, 70 mg/L CuSO_4_·5 H_2_O, 10 mg/L FeSO_4_·7 H_2_O, 8 mg/L MnCl_2_·4 H_2_O, 2 mg/L ZnSO_4_·7 H_2_O, 6 µg/L CoCl_2_·7 H_2_O). Moreover, we used R2 medium [57] (103 g/L sucrose, 21 g/L MOPS buffer, 0.25 g/L K_2_SO_4_, 10.12 g/L MgCl_2_·6 H_2_O, 10 g/L glucose, 0.1 g/L casamino acids, 1 mL KH_2_PO_4_, 8 mL CaCl_2_·2 H_2_O, 1.5 mL L-proline, 40 mg/L ZnCl_2_, 200 mg/L FeCl_3_·6 H_2_O, 10 mg/L CuCl_2_·2 H_2_O, 10 mg MnCl_2_·4 H_2_O, 10 mg/L Na_2_B_4_O_7_·10 H_2_O, 10 mg/L (NH_4_)_6_Mo_7_O_24_·4 H_2_O; pH adjusted to 7.2), SV2 medium [59] (15 g/L glucose, 15 g/L glycerol, 15 g/L soya peptone, and 1 g/L CaCO_3_; pH adjusted to 7.0), and GYM medium [57] (10 g/L malt extract, 4 g/L yeast extract, 4 g/L glucose).

To assess the origin of the nicotinic acid that was incorporated into the trioxacarcin derivatives, *Streptomyces* sp. 127Q was grown in a modified R5A medium [57], in which the 5 g/L of yeast extract, a source of nicotinic acid (0.7 g per 100 g of yeast extract) and tryptophan (0.9 g per 100 g) [60], was replaced with 5 g/L of casamino acids, containing no additional sources of nicotinic acid or tryptophan. Moreover, an additional cultivation was performed in this modified R5A medium, this time supplemented with either 2 mM nicotinic acid or 2 mM tryptophan. This was done to evaluate the effect of precursor availability on the production of nicotinic acid-containing trioxacarcins.

To assess the source of nicotinic acid and the production of the trioxacarcin/gutingimycin compounds, *Streptomyces* sp. 127Q was cultivated under the same conditions as for large-scale fermentation in R5A medium, as previously described.

Agar diffusion assay against *Lysinibacillus sphaericus* and *Paenibacillus alvei*: Sterile-filtered supernatant, extracts, and fractions were tested in agar diffusion assays against *L. sphaericus* DMSZ 1867 and *Paenibacillus alvei* DMSZ 29 on KB agar medium [61] plates (10 g/L proteose peptone, 1.5 g/L K_2_HPO_4_, 15 g/L glycerol, 5 mL of 1 M MgSO_4_, 15 g/L agar) and J broth agar medium plates (5 g/L tryptone-peptone from casein, 3 g/L K_2_HPO_4_, 15 g/L yeast extract, 2 g/L glucose, 20 g/L agar; pH adjusted to 7.3) [62], respectively. Prior to the bioassay, both test organisms were grown overnight in 10 mL of the respective liquid medium. For the assay, 30 μL of the cultured microorganisms were spread over the agar plates (size—150 mm × 15 mm) using a Drigalski spatula. Holes (ca. 0.5 cm in diameter) were punched into the agar using the flame-sterilized wide end of a Pasteur pipette. The supernatant, extracts, and test fractions were then applied to the holes (ca. 35–50 µL). The plates were then incubated overnight at 28 °C. Inhibition zones were documented by taking photos.

Bioassay-guided purification of trioxacarcin derivatives: The spent medium (12 L) was harvested by centrifugation (8000× *g*, 4 °C, 30 min). The collected supernatant was extracted three times with 1.5 volumes of ethyl acetate at pH 6.8. The extract was concentrated *in vacuo* yielding 1.2 g of a crude extract. However, bioactivity remained in the aqueous phase after ethyl acetate extraction. In addition, incomplete extraction was evident due to the strong characteristic UV-Vis absorption maxima at 260–270 nm and 400–430 nm, which were also partially observed in a crude ethyl acetate extract. Polar trioxacarcin-like compounds, which could not be fully extracted with ethyl acetate, were extracted by mixing the aqueous phase with Diaion HP-20 (30 g/L) resin overnight with gentle shaking at 100 rpm. The mixture was then packed into a column (4.5 × 35 cm). The resin was washed with 1 L of water. The metabolites bound to Diaion HP20 were eluted using a stepwise gradient with 2 L of 25%, 50%, and 75% of aqueous methanol, and 100% methanol, giving fractions F1, F2, F3, and F4, respectively. The bioactive F3 and F4 were concentrated to dryness *in vacuo*, yielding 1.2 g and 1.4 g of extract, respectively.

The dried ethyl acetate extract, F3 and F4 were each redissolved in 4–8 mL of methanol or a water/methanol mixture and subjected separately to MPLC. Purification was carried out using a Polygoprep C8 column (15 cm × 2 cm i.d., 50–60 µm particle size). The mobile phase consisted of solvent A (water 0.1% acetic acid) and solvent B (methanol 0.1% acetic acid). The flow rate was set to 8 mL/min. The gradient program was as follows: 30–50% B in 5 min; 50–65% B in 15 min; 65–80% B in 15 min; 80–100% B in 5 min, and 100% B for 5 min. One-minute fractions (8 mL) were collected in test tubes and freeze-dried.

The MPLC and subsequent HPLC fractions were analyzed for purity using LC-MS with either the LTQ or an Orbitrap XL. UV-Vis spectra were recorded from 200 to 800 nm. The HPLC separation program (mobile phase A) consisted of a 5 mM ammonium acetate buffer (pH 6.5) containing 10% acetonitrile. The for mobile phase B consisted of 90% acetonitrile with 10% of 5 mM ammonium acetate buffer (pH 6.5). Programmed elution was used at a flow rate of 0.2 mL/min employing a Nucleodur C8 Gravity column (250 × 2 mm, 5 µm). HPLC program (designated as LC-MS program 1): 15% B for 1 min; gradient elution from 15 to 25% B within 6 min; 25 to 35% B within 15 min; 35 to 100% B within 10 min, and 100% B for 5 min.

The semi-pure MPLC fractions F26–F30, obtained after the HP20 fractionation (F3 and F4), contained bioactive compound **1** and were combined and designated as fraction **S1**. Similarly, the MPLC fractions F31–F34, derived from the MPLC fractionation of the ethyl acetate extract, contained compound **2** and were therefore pooled and designated as **S2**. **S1** and **S2** were then purified by semi-preparative HPLC fractionation.

**S1** (F26–F30, 64 mg) was separated using a Nucleodur Sphinx RP column (250 × 4.6 mm, 5 µm) at a flow rate of 0.6 mL/min. A volume of 35 µL was injected per run (65 runs). The mobile phase consisted of solvent A (10 mM ammonium acetate buffer containing 10% acetonitrile (pH 6.5, adjusted with acetic acid)) and solvent B (90% acetonitrile containing 10% of 10 mM ammonium acetate buffer (pH 6.5, adjusted with acetic acid)). The HPLC program was 15% B for 1 min; 15–25% within 10 min; 25–28% B within 6 min; 28–30% B within 4 min, 30–60% within 6 min; 60–100% B within 4 min; 100% B for 5 min. Fractions were collected from minute 10.5 onwards at 0.5 min fraction size and lyophilized. Under these conditions, compound **1** eluted at t = 18.1 min, yielding 3.8 mg from 12 L of spent medium.

**S2** (F31–F34, 36 mg) was purified using a Nucleodur Isis C18 column (250 × 4.0 mm, 5 µm) at a flow rate of 0.6 mL/min. A volume of 30 µL was injected per run (40 runs). A 10 mM ammonium acetate buffer containing 10% acetonitrile (pH 6.5 adjusted with acetic acid) served as solvent A, and acetonitrile 0.1% acetic acid (pH 6.5) served as solvent B. The HPLC program was 15% B for 1 min; 15–25% B within 5 min; isocratic condition 10 min at 25% B; 25–40% B within 7 min; 40–90% B within 7 min; 90% B for 5 min. Fractions were collected from minute 12.5 min every 0.5 min and lyophilized. In these conditions, compound **2** eluted at t = 22.1 min, yielding 1.6 mg from 12 L spent medium.

Structure elucidation of trioxacarcin derivatives from *Streptomyces* sp. 127Q: High-resolution mass spectra (HR-HESI-MS) were recorded in positive ionisation mode using an LTQ Orbitrap XL mass spectrometer. A hit list of molecular formulas was determined with Δppm 3–5, and the search space was narrowed down by determining the number of carbons using the ^13^C isotope peak. Moreover, MS/MS spectra were recorded to identify indicative losses (sugar moieties). ^1^H NMR, ^1^H,^1^H-Cosy, HSQC, HMBC, ROESY, and ^13^C NMR spectra were recorded and analyzed to determine the structures of the purified trioxacarcins **1** and **2**. UV-Vis spectra were analyzed to identify specific chromophores. Additionally, the biosynthetic gene cluster (BGC), predicted by AntiSMASH [63], supported the structural elucidation (see below).

Hydrolysis of trioxacarcin 780 (**1**): Trioxacarcin 780 (**1**, 300 µg) was dissolved in methanol (30 µL). Then, 6 M KOH (60 µL) was added to the mixture, which was incubated at 99 °C for 16 h. After cooling to room temperature, the reaction mixture was neutralized with 40 µL of 6 M HCl. The solvent was blown off with a gentle nitrogen flow, and the sample was reconstituted in 300 µL of distilled water. The sample (1 µL) was injected into the LC-MS system and screened for the suspected nicotinic acid. The HPLC conditions (designated as LC-MS program **2**) were Phenomenex Synergi Polar RP column (250 × 2 mm, 4 µm). The mobile phase consisted of solvent A (water 0.1% acetic acid) and solvent B (acetonitrile 0.1% acetic acid), with a flow rate of 0.2 mL/min. HPLC program: 2% B for 2 min; 2–95% B within 12 min; 95% B for 5 min. The retention time of the nicotinic acid in the hydrolysate (retention time: 5.9 min) was compared to that of an authentic nicotinic acid standard.

Study on the incorporation of nicotinic acid into trioxacarcin 780 (**1**): To explain the possible incorporation of nicotinic acid into the biosynthesis of trioxacarcin 780 (**1**), *Streptomyces* sp. 127Q was cultivated in the aforementioned regular R5A medium, and a modified version of the R5A medium in which 5 g/L of yeast extract was replaced with 5 g/L of casamino acids. Moreover, a modified R5A medium, containing 5 g/L of casamino acids (without yeast extract) and supplemented with either 2 mM of nicotinic acid or 2 mM of tryptophan, was used. In parallel, other media were tested, namely, R2, SYG, GYM, and SV2. The cultivation conditions were the same as those described earlier for fermentation in R5A. After 21 d of growth, the cultures were harvested (10,000 g, 30 min, 4 °C), and the supernatant was mixed with Diaion HP20 resin (30 g/L) and stirred overnight at 100 rpm. The bound metabolites were eluted with 100% methanol. The extracts were subsequently analyzed by LC-MS.

Time course of trioxacarcins formation: Here, 500 mL Erlenmeyer flasks containing 100 mL of R5A medium were inoculated with precultures of different ages (2, 3, and 4 d). The production of trioxacarcins by *Streptomyces* sp. 127Q was monitored over a period of 26 d, with samples harvested (10,000 g, 30 min, 4 °C) every 3–5 d. Each sample (1.5 mL of culture supernatant) was extracted with ethyl acetate (1.5 mL) at the native pH of the culture (pH 6.8). The extracts were subsequently analyzed by LC-MS to monitor the production levels and the temporal appearance of all detected trioxacarcin metabolites.

Identification of further trioxacarcin and gutingimycin variants: The sample was obtained after culturing *Streptomyces* sp. 127Q in 4 L of R5A for 21 d, followed by the usual extraction procedure described earlier using Diaion HP20 resin. The F3 and F4 eluates were concentrated *in vacuo* to dryness, and redissolved in 50 mL of methanol or a water/methanol mixture. Equal aliquots (2 mL) of each F3 and F4 were then combined. A volume of 10 µL (corresponding to ca. 4.35 µg/µL) was injected into the LC-MS system. Data-dependent scanning was employed for MS/MS fragment acquisition. The spectrum files were uploaded directly to the Global Natural Product Social Molecular Network website (GNPS) website [10], and a molecular network was created using the default online workflow. The precursor ion mass tolerance was set to 2.0 Da, and the fragment ion mass tolerance to 0.1 Da. Network edges were filtered to have a cosine score above 0.7, and more than four matched peaks. Edges between two nodes were retained only if each node appeared in the other’s ten most similar nodes.

Purification of gutingimycin A (**7**) from *Streptomyces* sp. 127Q: From a two-month-grown culture (6 L) of *Streptomyces* sp. 127Q, the supernatant of spent medium was collected by centrifugation (8000× *g*, 4 °C, 30 min). The spent medium was incubated over night with Diaion HP20 (250 g). The Diaion HP20 resin was transferred into a glass column, the spent medium was eluted and the resin was washed with 500 mL water, 500 mL water:MeOH (50:50), and with 750 mL MeOH. Gutingimycin A (**7**) containing fractions was combined and subjected to MPLC purification (fraction size: 1 min) using gradient elution from 20% to 100% B in 30 min, and 100% B for 5 min at a flow rate of 15 mL/min. We used reversed-phase Polygoprep C8 resin (20 cm × 2 cm i.d., 50–60 µm particle size, solvent A—water and solvent B—MeOH). The gutingimycin A (**7**)-containing fractions were further purified by HPLC fractionation using the Nucleodur ISIS column. HPLC conditions: solvent A—water, solvent B—acetonitrile, flow rate—0.6 mL/min, HPLC program—30% B for 1 min, gradient to 100% B in 24 min, for 5 min 100%B, in 1 min to 30%B, re-equilibration for 5 min at 30% B, injection volume 10 µL, 80 times. Fractions (fraction size: 0.25 min) from 9 to 9.5 min contained pure gutingimycin A (**7**). The fractions were dried under vacuum at 40 °C using a rotavap. Pure gutingimycin A (**7**) (3.5 mg) was analyzed by NMR and its optical rotation was determined.

Optical rotation measurement of gutingimycin A (**7**): The optical rotation of HPLC-purified gutingimycin A (**7**) (0.58 mg/mL in CHCl_3_) was determined using a Jasco 2000 polarimeter at 589 nm at 22 °C.

Genome sequencing of *Streptomyces* sp. 127Q and identification of the trioxacarcin biosynthetic gene cluster: To isolate the genomic DNA, *Streptomyces* sp. 127Q was grown in a liquid GYM medium for 48 h. The pellet was then collected by centrifugation (5000× *g*, 15 min, 4 °C). Genomic DNA extraction, genome sequencing, and sequence assembly into contigs were performed by Novogene (Munich, Germany). A 150 bp paired-end library (3 Gb) was generated and sequenced using the Nova Seq X Plus platform (Illumina GmbH, Berlin, Germany). The draft genome was analyzed for its secondary metabolite biosynthetic gene clusters using antiSMASH [63]. To compare the trioxacarcin BGCs from different *Streptomyces* strains with the trioxacarcin BGC of *Streptomyces* sp. 127Q, clinker v0.0.28 was used with the default parameters [64]. The trioxacarcin BGC sequences were retrieved from GenBank using search tools such as antiSMASH [63] and cblaster v1.3.18 [65].

Antibacterial assays against human and bee pathogens: The antibacterial activities of trioxacarcin 780 (**1**) and trioxacarcin 692 (**2**) were evaluated using the broth microdilution method in a 96-well plate. HPLC pure samples of trioxacarcin 780 (**1**) and trioxacarcin 692 (**2**) were used for the antibacterial assays. The optical densities at 600 nm (OD_600_) were measured using a Berthold Apollo microplate reader (Bad Wildbad, Germany). The following test microorganisms and their growth conditions were used: *P. alvei* was cultured in J broth medium and on J broth agar plates [62]; *L. sphaericus* was grown in KB medium and on KB agar plates [61].

Clinical isolates *Klebsiella pneumoniae*, *Escherichia coli*, *Staphylococcus aureus*, and *Staphylococcus epidermidis* were cultured in LB medium (10 g/L tryptone, 5 g/L yeast extract, 10 g/L NaCl, 20 g/L agar; pH 7.0) and on LB agar plates [66]. *Moraxella catarrhalis* and *Neisseria gonorrhoeae* were cultivated in liquid PPM medium (15 g/L proteose peptone, 1 g/L soluble starch, 5 g/L NaCl, 4 g/L KH_2_PO_4_, 1 g/L K_2_HPO_4_, pH 7.5) [67] and on GC agar plates (BD Difco, GC Medium Base) [68], both supplemented with 1% IsoVitale vitamin mix (100 g/L glucose, 10 g/L glutamine, 26 g/L L-cysteine, 100 mg/L cocarboxylase, 250 mg/L nicotinamide adenine dinucleotide, 20 mg/L Fe(NO_3_)_3_, 150 mg/L arginine, 3 mg/L vitamin B_1_, 10 mg/L vitamin B_12_, 13 mg/L p-aminobenzoic acid, 1.1 g/L cystine, 1 g/L adenine, 500 mg/L uracil, 30 mg/L guanine in ddH_2_O; pH 7). Prior to the antimicrobial assay, each strain was streaked out twice on a solid medium to ensure purity. The microorganisms were cultivated overnight at 37 °C in 5% CO_2_ (solid medium). On the third day, the bacterial cultures were collected from the plates with a sterile cotton swab and inoculated into 5–10 mL of the corresponding liquid medium for 2–3 h at 37 °C with 220 rpm to reach the logarithmic growth phase. For the bioassay, the pre-cultures were diluted to an OD_600_ of 0.2 to reach a final OD_600_ of 0.1 in the well.

Each 96-well plate assay included a medium blank, a medium blank containing the test compound (at the highest concentration, 150 µM), a growth control, a positive control, and a solvent control. The medium blank contained 100 μL of medium. The medium blank with the test compound contained 99 µL of the corresponding medium, to which 1 µL of the test compound was added. The growth control consisted of 50 μL of medium and 50 μL of grown culture. The solvent control contained 50 μL of medium, 49 μL of culture, and 1 μL of DMSO. The positive control contained 50 μL of medium, 49 μL of culture, and 1 μL of the respective established antibiotics (vancomycin or ciprofloxacin) in DMSO. The treated samples contained 50 μL of medium, 49 μL of culture, and 1 μL of either trioxacarcin 780 (**1**) or trioxacarcin 692 (**2**) in DMSO. Stock solutions of the test compounds and positive controls were prepared at a concentration of 15 mM in DMSO. Trioxacarcin 780 (**1**) and trioxacarcin 692 (**2**) were serially diluted to obtain final assay concentrations ranging from 150 to 9.4 µM, corresponding to a maximum of 1% (*v*/*v*) of DMSO in the wells. The positive control antibiotics, vancomycin and ciprofloxacin, were tested in parallel using final concentrations ranging from 37.5 to 0.3 µM. For the preliminary screening, the test compounds were tested in one replicate at the highest concentration (150 µM). Based on the results of this preliminary screening, *E. coli*, *S. aureus*, *L. sphaericus*, and *N. gonorrhoeae* were selected to evaluate the antimicrobial activity of trioxacarcin 780 (**1**) and trioxacarcin 692 (**2**) at concentrations ranging from 150 to 9.4 µM. All samples and controls were inoculated from the same preculture, in three replicates (*n* = 3).

For the photochemical bioassay [69], a second 96-well plate was prepared in parallel using the same method. This plate was irradiated using an LED plant growth lamp (λ = 465 + 620 nm, blue light photon flux density 100 μmol m^−2^ s^−1^ and red light photon flux density 70 μmol m^−2^ s^−1^, t_PI_ = 60 min pre-illumination time), while the first plate was placed in the dark as a control (dark condition) [69]. During irradiation, the incubation temperature remained constant at 26 °C. The LED lamp did not warm up the 96-well plate. After irradiation, both plates were incubated in the dark for 20 h at 37 °C, with 550 rpm. Growth was monitored after 7 h and 20 h.

Statistics: Data from the bioassays were evaluated by subtracting the initial OD_600_ (time point 0 h) from the OD_600_ measured after 7 h or 20 h of incubation. To account for background absorbance caused by the coloured test compounds, the ΔOD_600_ values were corrected by subtracting the OD_600_ of a medium blank containing the highest concentration of the tested compound (150 µM). Negative values were set to zero. Data are presented as the mean ± standard deviation (SD) of three replicates.

The statistical significance of the differences between the groups treated with compounds and the group treated with the solvent control (1% DMSO) was assessed as indicated in the figure legends, using a one-way analysis of variance (ANOVA) followed by a Dunnett’s multiple comparison test in Prism 11 (GraphPad, Boston, MA, USA). Statistical significance was defined as *p* < 0.05 (*), *p* < 0.01 (**), *p* < 0.001 (***) and *p* < 0.0001 (****).

Percentage inhibition was calculated from mean ΔOD_600_ values (*n* = 3) relative to the solvent control (*NC*; negative control) corresponding to 1% DMSO using ΔOD_600_ values according to the following formula:

$$\%Inhibition=(1-\left(\frac{mean\;\Delta OD600,sample}{mean\;\Delta OD600,NC}\;\right))\times 100$$

A heatmap of percentage inhibition across bacterial strains was generated using the ggplot2 package (version 3.4) in R (version 4.3.1).

## 3. Results

### 3.1. Bioassay-Guided Purification and Identification of Trioxacarcins from Streptomyces sp. 127Q

*Streptomyces* sp. 127Q, which was isolated from the stingless bee *T. carbonaria*, was cultivated in an R5A liquid medium for 21 days. Every 3–4 days, supernatant was harvested, sterile-filtered, and tested in an agar diffusion bioassay against *L. sphaericus* and *P. alvei*. Strong activity was observed on days 18–21. An ethyl acetate extract was subsequently prepared from this culture and tested in an agar diffusion assay to verify the extraction efficiency (Figure S1). Although the ethyl acetate extract inhibited the test organisms, residual bioactivity was detected in the aqueous supernatant, indicating incomplete extraction. Therefore, the residual supernatant was mixed with Diaion HP20 resin to bind its bioactive secondary metabolites. The antimicrobial metabolites were eluted into four fractions using aqueous methanol at 25%, 50%, and 75%, with the final fraction being eluted using 100% methanol. The last two fractions were bioactive, while the flow-through was inactive. To obtain a sufficient yield of the bioactive compounds, the cultivation of *Streptomyces* sp. 127Q was scaled up to 12 L.

The crude ethyl acetate extract and the bioactive Diaion HP20 resin fractions (F3 and F4) were both concentrated *in vacuo*. The bioactive crude ethyl acetate extract (1.2 g), F3 (1.2 g), and F4 (1.4 g) Diaion HP20 eluates were fractionated separately by RP8 MPLC. Alternatively, trioxacarcins and gutingimycins can be purified using Diaion HP20, thereby eliminating the need for the ethyl acetate extraction step. The contiguous MPLC fractions (F26–F30), designated as **S1** (64 mg), were combined and further separated by HPLC using a Nucleodur Sphinx RP column, yielding the pure antimicrobial compound **1** (3.8 mg, 0.32 mg/L). The MPLC fractions (F31–F34), designated as **S2** (36 mg), were combined and purified using a Nucleodur Isis C18 column, yielding the pure compound **2** (1.6 mg, 0.13 mg/L). While the yields were quite low, this is not unusual for trioxacarcins, such as trioxacarcin A (**3**) and B (**4**) (0.3–1.1 mg/L) [53].

**Figure 1:**
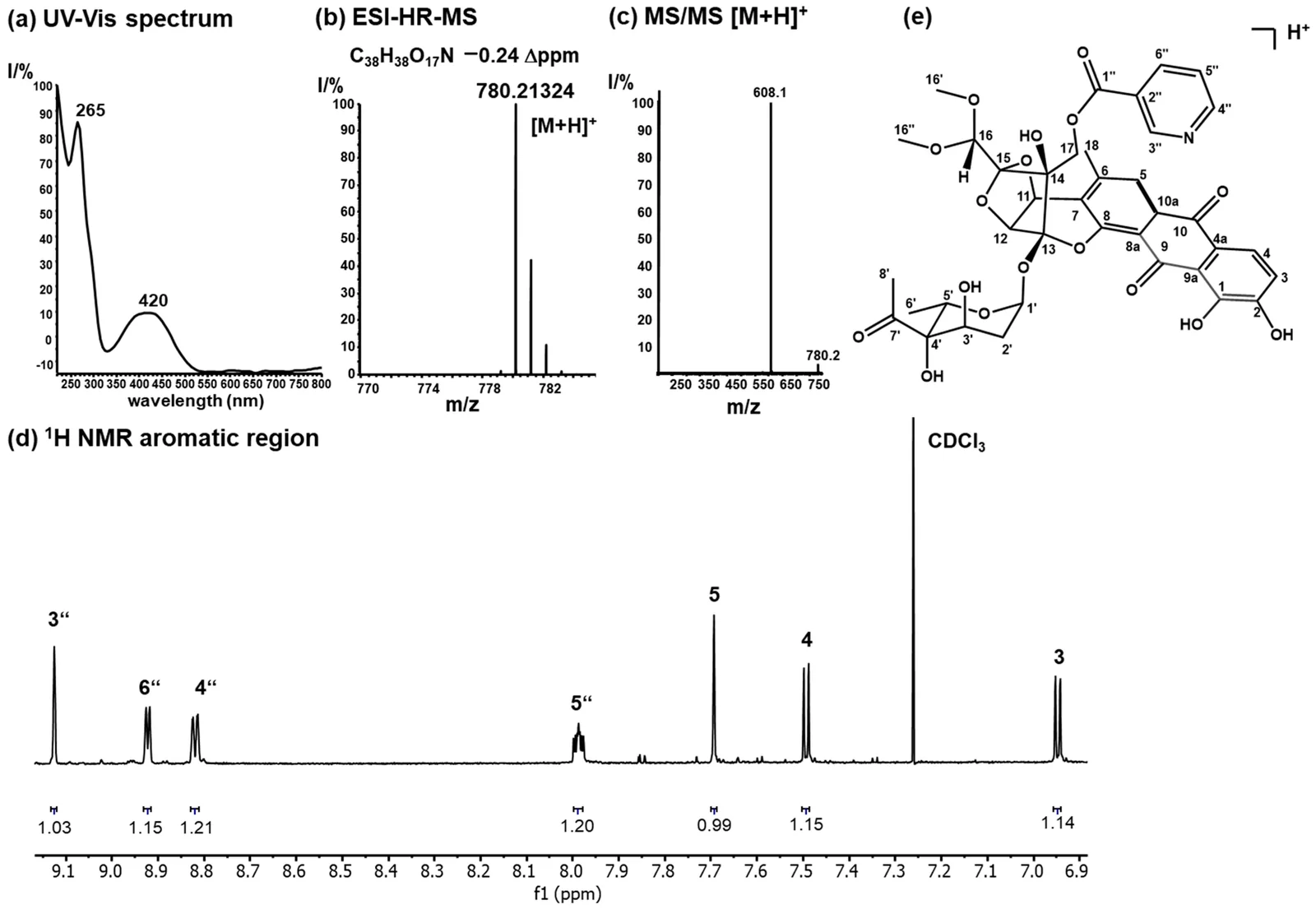
Identification of trioxacarcin 780 (**1**). (**a**) UV-Vis spectrum, (**b**) HR-ESI-MS spectrum, (**c**) MS/MS spectrum of the [M+H]^+^ quasimolecular ion, (**d**) aromatic region of the ^1^H NMR spectrum, (**e**) structure of trioxacarcin 780 (**1**).

Compound **1** appeared as an amorphous, reddish-purple powder. LC-UV-Vis-HR-ESI-MS analysis revealed that **1** eluted at 20.2 min. The UV-Vis spectrum of **1** exhibited λ_max_ at 265 and 425 nm in 90% methanol with 10% 10 mM ammonium acetate buffer (Figure 1 and Figure S2). Absorbance at 425 nm is characteristic of 1,2-dihydroxyanthraquinones, such as alizarin (430 nm in methanol) [70]. In 1 N HCl/MeOH (1:9) at pH 3, the antibiotic **1** appeared bright yellow, and its UV-Vis absorption maxima shifted to λ_max_ 230, 270, and 420 nm. In 1 N KOH/MeOH (1:9) at pH 9, compound **1** appeared dark blue, with a shift in the UV-Vis absorption maxima to λ_max_ 265 and 430 nm. This behavior is consistent with that observed in extended aromatic ring systems, such as 1,2-dihydroxyanthraquinones, which contain phenolic hydroxy moieties. Deprotonation/protonation changes the aromatic system, causing different color impressions [70]. Compound **1** yielded a [M+H]^+^ quasimolecular ion at *m*/*z* 780.21324, suggesting a molecular composition of C_38_H_38_O_17_N (Δppm −0.240, Figure 1 and Figure S3). MS/MS fragmentation of the [M+H]^+^ ion furnished an intense ion at *m*/*z* 608, corresponding to a loss of 172 Da (C_8_H_13_O_4_), indicative of a sugar-like moiety (Figure 1 and Figure S4).

To identify the structure of antibiotic **1**, the following spectra were recorded: ^1^H NMR, ^13^C NMR, COSY, HSQC, HMBC and ROESY (Figure 1, Figures S5–S14 and Table S1). The NMR spectra of **1** were recorded in CDCl_3_ and CD_3_OD with the addition of a tiny amount of diluted DCl. This was necessary because **1** exhibited only broad, poorly resolved signals in the aromatic region of the ^1^H NMR in both CD_3_OD and CDCl_3_. However, compound **1** was found to be labile to acid treatment, and thus the careful use of diluted acid was needed.

The ^1^H NMR spectrum of the antibiotic **1** showed both differences and similarities to the ^1^H NMR spectra of trioxacarcins, such as trioxacarcin A (**3**) and trioxacarcin B (**4**), and gutingimycins, such as gutingimycin A (**7**) (Figure 1 and Figures S5–S8) [54,55,71,72,73,74]. The NMR of **1** had signals characteristic of the α-L-trioxacarcinose B moiety [4′-C-acetyl-2′,6′-dideoxy-α-L-glucose, (**5**)] moiety (5.44, 1.67–1.70, 3.66–3.68, 4.54, 0.84, 2.06/2.08 ppm), but not the α-L-trioxacarcinose A moiety [4′-*epi*-α-L-mycarose (**6**)] moiety. The α-L-trioxacarcinose B (**5**) moiety was cleaved already with 2 N HCl at room temperature within 2 h, giving the sugar free aglycon trioxacarcin 608 (**8**). This compound was also present in the crude extract of *Streptomyces* sp. 127Q (Figures S15–S17).

Signals caused by the non-aromatic ring system of the aglycon of trioxacarcins were also present in the NMR spectra of **1** [CH(11) 4.98 ppm, CH(12) 5.14 ppm, CH(16) 4.79 ppm, CH_2_(17) 4.44 and 4.83 ppm] (Figures S5–S13). However, two ^1^H NMR signals for the aromatic core of the aglycon of **1** [CH(3) 6.94 and CH(4) 7.49 ppm] appeared in addition to the common signal CH(5) 7.69 ppm in trioxacarcin A (**3**) and B (**4**) [54,71], as well as the related gutingimycin A (**7**) [55]. The analysis of the aromatic region of the ^1^H NMR and ^1^H, ^1^H COSY of **1** indicated the presence of an isolated aromatic ring (7.97–8.00, 8.82, 8.92, 9.12 ppm), a nicotinic acid moiety, and a 1,2-substituted aromatic ring in the aglycon (Figure 1, Figures S5–S8 and S10). The presence of the nicotinic acid ester moiety was proven by hydrolyzing **1**. Drastic conditions were required for this hydrolysis; 6 M KOH, 99 °C for 16 h. Free nicotinic acid was detected by LC-MS at *m*/*z* 124, with an identical retention time to the nicotinic acid standard (Figure S18). The resistance of the sterically hindered nicotinic acid ester of **1** to mild acidic or alkaline hydrolysis is consistent with other nicotinic acid ester-containing natural products [75,76]. The nicotinic acid ester moiety in trioxacarcin 780 (**1**) was deduced to be attached at the hydroxyl moiety of position C17 due to the ROESY correlations of the C17 hydrogens (4.44 ppm, 4.83 ppm) to the nicotinic acid protons at C(3″) (9.12 ppm), and much weaker to C(4″) (8.92 ppm) (Figure S13). ^1^H, ^13^C, ^1^H, ^1^H COSY, HSQC, and HMBC correlations, as well as the UV-Vis spectrum (Figures S2, S5–S14 and Table S1), verified the 1,2-dihydroxyanthraquinone core structure of **1**. To verify the 1,2-dihydroxyanthraquinone moiety in particular, the following ^1^H−^13^C-HMBC correlations were important: CH_3_(18) 2.41 ppm to C(10a) 117.72 ppm, C(7) 122.13 ppm and C(5) 123.41; CH(5) 7.69 ppm to CO(10) 180.52 ppm, C(7) 122.13 ppm, C(8a) 117.91 ppm and C(18) 19.21 ppm; CH(4) 7.49 ppm to CO(10) 180.52 ppm, C(2) 153.04 ppm and C(9a) 116.67 ppm; CH(3) 6.94 ppm to C(1) 150.46 ppm and C(4a) 123.27 ppm. Due to its similarity to trioxacarcins and its distinctive quasimolecular ion, we named **1** trioxacarcin 780. The analysis of the ^1^H, ^1^H COSY, HSQC, HMBC and ROESY spectra allowed the complete assignment of the NMR signals to the atoms in trioxacarcin 780 (**1**), as presented in Table S1. The key ^1^H, ^1^H COSY and HMBC correlations that verified the structure of **1** are given in Figure S14.

The stereochemistry of trioxacarcin 780 (**1**) could not be clearly identified by NMR, since many of its stereocenters lack neighboring hydrogens exhibiting a characteristic coupling pattern. We also had to run trioxacarcin 780 (**1**) in a different NMR solvent than that used for related trioxacarcins, due to its different solubility [71,72,73,74]. This means that the NMR spectra of **1** could not be directly compared to the established trioxacarcins/gutingimycins NMR data. However, the stereochemistry of gutingimycin A (**7**) was previously identified by X-ray crystallography, and its optical rotation was determined ([α]^20^_D_= −56.5, CHCl_3_) [55]. In order to address the stereochemistry of the trioxacarcins/gutingimycins from *Streptomyces* sp. 127Q, we purified gutingimycin A (**7**) from *Streptomyces* sp. 127Q. The UV, MS, and NMR spectra (Figures S19–S27 and Table S2) and the specific optical rotation ([α]^22^_D_= −56.2, CHCl_3_) of gutingimycin A (**7**) from *Streptomyces* sp. 127Q matched the data reported by Maskey et al. for *Streptomyces* B8652 [55]. Because the core structure of gutingimycins and trioxacarcins is produced by the same enzymes, the stereochemistry of the stereocenters of compounds **1** and **2** from *Streptomyces* sp. 127Q is identical to that of gutingimycin A (**7**).

The antimicrobial compound **2** eluted in the LC-MS at a retention time of 27.6 min. The UV-Vis spectrum of compound **2** had absorption maxima at λ_max_ 235, 265, and 425 nm in 90% methanol with 10% 10 mM ammonium acetate buffer, appearing in a bright yellow color (Figure S28). In 1 N HCl/MeOH (1:9) at pH 3, while compound **2** remained bright yellow, and its UV-Vis spectrum exhibited the same absorption maxima as in methanol/ammonium acetate. In 1 N KOH/MeOH (1:9) at pH 9, compound **2** appeared with a light purple color, and its UV-Vis absorption spectrum exhibited absorption maxima at λ_max_ 235, 260, and 420 nm. These UV-Vis spectra were highly similar to those spectra recorded for trioxacarcin 780 (**1**), indicating the presence of the same 1,2-dihydroxyanthraquinone aromatic core as in trioxacarcin 692 (**2**). In the mass spectrometer, compound **2** gave a [M+NH_4_]^+^ ion at *m*/*z* 692.21887, matching the molecular composition of C_32_H_38_O_16_N (Δppm 0.360, Figure S29). MS/MS fragmentation of the [M+NH_4_]^+^ ion yielded an ion at *m*/*z* 503, corresponding to the loss of 172 Da, indicative for trioxacarcinose B (**5**), followed by the loss of methanol (*m*/*z* 471) (Figure S30). The comparison of the molecular formulas of **1** and **2** indicated a difference of C_6_H_3_ON, corresponding to the difference of a hydroxyl group instead of a nicotinic acid ester moiety at carbon 17. This deduction was verified by the NMR data; ^1^H NMR, ^1^H,^1^H COSY, HSQC, and HMBC (Figures S31–S37). The assignments of NMR signals to the atoms of compound **2** are given in Table S3, and the key ^1^H, ^1^H COSY and HMBC correlations are indicated in Figure S38.

**Figure 2:**
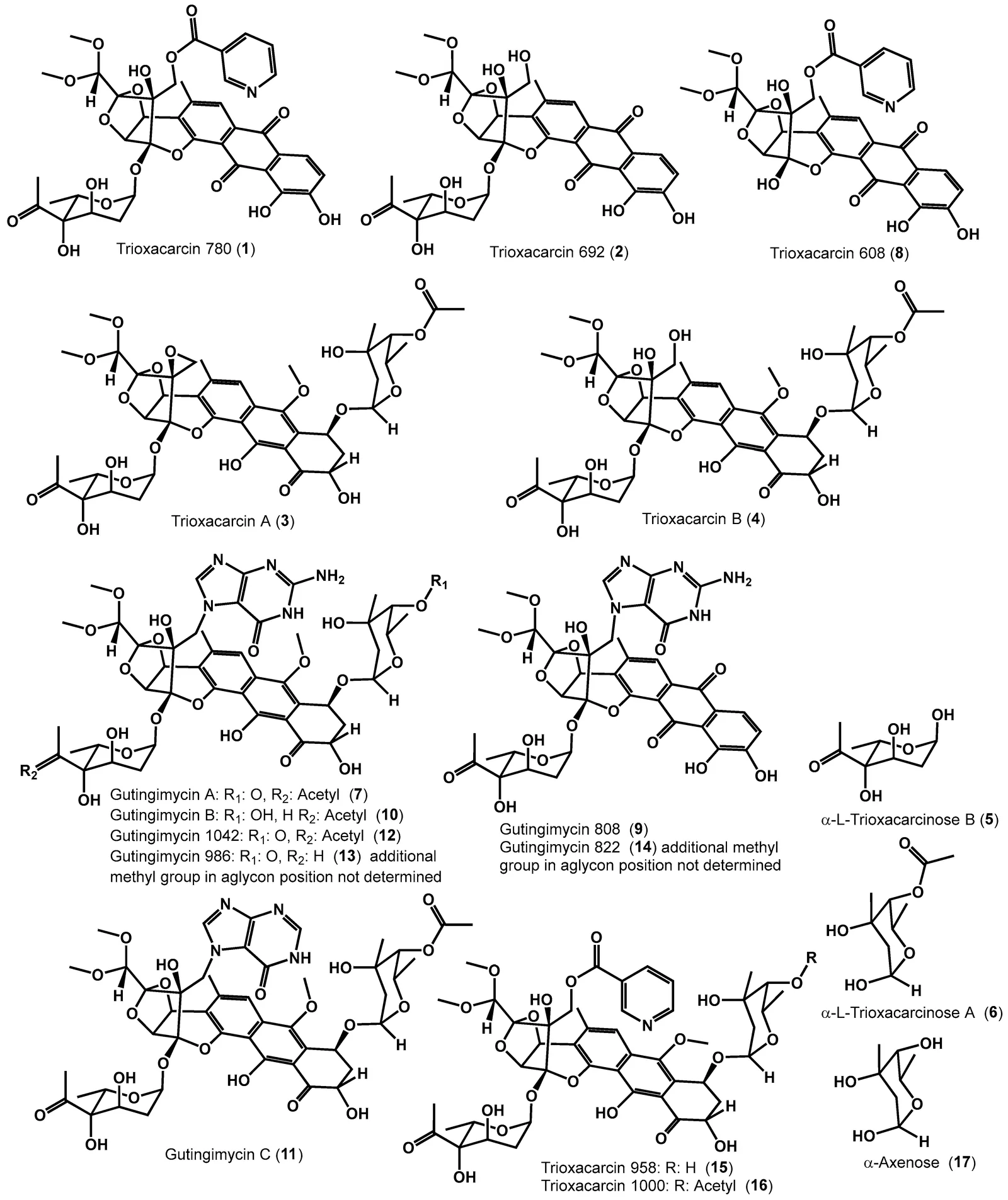
Diversity of trioxacarcins and gutingimycins from *Streptomyces* sp. 127Q, which produces previously unknown trioxacarcins with a 1,2-dihydroxyanthraquinone aglycon and trioxacarcins/gutingimycins bearing a nicotinic acid ester moiety. Moreover, respective epoxide precursors, such as trioxacarcin A (**3**) [54], were not or hardly detectable by LC-MS, indicating their rapid conversion. Gutingimycin 808 (**9**) was previously observed during X-ray crystallization from synthetic trioxacarcin A with the duplex oligonucleotide d(CGTATACG) [77].

Alongside the LC-MS and NMR analyses of the purified antimicrobials of *Streptomyces* sp. 127Q, its genome was sequenced in order to allow screening for the secondary metabolite biosynthetic gene clusters (BGCs) using antiSMASH [63]. The only gene cluster that matched the deduced structural properties of compounds **1** and **2** exhibited 80% similarity to the trioxacarcin BGC. Screening for trioxacarcins in *Streptomyces* sp. 127Q extracts using LC-MS yielded the closely related gutingimycin A (**7**) and traces of trioxacarcin B (**4**). However, compounds **1** and **2** differ strongly from common trioxacarcins [54,71,73,74] and gutingimycins [55], due to their altered aromatic core and the absence of the trioxacarcinose A (**6**) moiety, and the presence of a nicotinic acid ester moiety (Figure 2). The closely related 1,2-dihydroxyanthraquinone gutingimycin 808 (**9**) was observed previously by Pröpper et al. when they crystalized synthetic trioxacarcin A (**3**) with the duplex oligonucleotide d(CGTATACG), as observed during X-ray crystallization from [77].

### 3.2. Incorporation of Nicotinic Acid into Trioxacarcins

Trioxacarcin 780 (**1**) is the reaction product of its epoxide precursor with nicotinic acid. As trioxacarcins containing a nicotinic acid ester moiety were unknown up to now, we questioned whether they are formed due to the presence of 5 g/L of yeast extract in R5A medium, which contains nicotinic acid (0.7 g per 100 g of yeast extract) and tryptophan (0.9 g per 100 g) [60]. Consequently, *Streptomyces* sp. 127Q was cultivated under various fermentation conditions. In one set of experiments, the yeast extract in the R5A medium was replaced with 5 g/L casamino acids to maintain the availability of the nitrogen source, as a negative control. In parallel, cultures were prepared in the same way, but supplemented with either 2 mM nicotinic acid or 2 mM tryptophan. The modified R5A medium, in which the yeast extract was replaced with casamino acids, also supported the production of nicotinic acid-containing trioxacarcin derivatives. However, LC-MS analysis revealed low production levels in this condition (signal intensity ~10^5^), whereas cultures supplemented with either 2 mM nicotinic acid or 2 mM tryptophan exhibited increased production (~10^6^). The highest abundance of trioxacarcin 780 (**1**) (~10^7^) was observed in unmodified R5A medium (Figure S39). Also, when *Streptomyces* sp. 127Q was cultivated in an R2 medium, which does not contain nicotinic acid, trioxacarcin 780 (**1**) was formed, indicating that *Streptomyces* sp. 127Q can use its own produced nicotinic acid to synthesize trioxacarcin 780 (**1**).

### 3.3. Diversity of Trioxacarcins and Gutingimycins in Streptomyces sp. 127Q: GNPS Molecular Networcking and LC-UV Profiling

Apart from trioxacarcin 780 (**1**) and trioxacarcin 692 (**2**), screening the UV-Vis and LC-MS chromatograms of the *Streptomyces* sp. 127Q supernatants and extracts revealed that *Streptomyces* sp. 127Q produces a variety of trioxacarcins/gutingimycins (Figure 2 and Figure S40). Molecular networking analysis [10] confirmed the presence of many trioxacarcin variants suspected from the UV-Vis and LC-MS analyses (Figure 2 and Figures S40, S41). The structures of many abundant trioxacarcin variants were deduced by analysis of their UV-Vis, HR-ESI-MS, and MS/MS spectra (Figure 2 and Figures S42–S65). Both the epoxide ring-opened gutingimycins (**7**, **9**, **10**, **11**, **12**, **13**, **14**) and trioxacarcins with 1,2-dihydroxyanthraquinone aglycons (**1**, **2**, **8**, **15**, **16**), which lack the α-trioxacarcinose A (**6**) moiety, were found in *Streptomyces* sp. 127Q, with variations in the products of trioxacarcin epoxides having reacted either with water, guanine, or nicotinic acid (Figure 2).

Moreover, subtle modifications occurred in the methylation pattern of the aglycon, or the α-L-trioxacarcinose A (**6**) or α-L-trioxacarcinose B (**5**) moiety was seen to be replaced by the α-axenose (**17**) moiety, which lacked the acetylation and reduction of the oxo group in the α-L-trioxacarcinose B (**5**), and sugar moiety can occur (Figure 2). Additionally, two more compounds were linked to the molecular networking of trioxacarcins and gutingimycins. The compounds exhibited [M+H]^+^ ions at *m*/*z* 1100.38167 and *m*/*z* 865.26470, and appeared to be members of the trioxacarcins/gutingimycins family (Figure S41). In order to identify them, they will need to be purified to obtain 1D and 2D NMR datasets, which should be subjects of future experiments. Because of the different polarity of trioxacarcin derivatives, and our choice of ethyl acetate for the extractions, it is possible that further trioxacarcin derivatives may be found in *Streptomyces* sp. 127Q and may have escaped our profiling.

Ethyl acetate quite efficiently extracted the major metabolites; however, substantial bioactivity and characteristic UV-Vis absorption signals were still observed in the aqueous phase. Therefore, the aqueous fraction was subsequently subjected to adsorption on Diaion HP-20 resin to recover the remaining metabolites. This observation is consistent with earlier published reports on trioxacarcin- and gutingimycin-producing strains *S. bottropensis* DO-45 [53] and *Streptomyces* B8652 [71]. Tamaoki et al. combined Diaion HP-20 adsorption with ethyl acetate extraction during the isolation of trioxacarcin, highlighting the limitations of relying on a single extraction method for these metabolites [53]. Similarly, Maskey et al. reported the incomplete recovery of gutingimycin by ethyl acetate extraction, and therefore employed an additional extraction procedure with methanol after the concentration of the culture filtrate [71]. Together, these studies suggest that trioxacarcin-related compounds can differ substantially in polarity and extraction behavior, requiring a combination of extraction and adsorption techniques for their efficient recovery.

The influence of different growth media (SV2, GYM, SYG, and R2A) on the production of trioxacarcins by *Streptomyces* sp. 127Q was studied (Figure S66). Although the SYG and GYM media were previously used for trioxacarcin production by *Streptomyces bottropensis* DO-45 [73] and the marine *Streptomyces* sp. B8652 [71], *Streptomyces* sp. 127Q did not produce any trioxacarcins in these media. The R2 medium supported the production of trioxacarcin 780 (**1**) and trioxacarcin 692 (**2**) in low amounts, although the metabolic profile differed substantially from that observed in the R5A medium (Figure S66).

To further investigate the formation of trioxacarcins, particularly when epoxides may be produced, a time-course experiment was conducted. The samples were withdrawn every 3–5 days until day 26. LC-MS analysis revealed that trioxacarcin 780 (**1**), trioxacarcin 692 (**2**), and trioxacarcin 1000 (**16**) were the most abundant, and appeared as early as on day 7. Their concentrations steadily increased throughout the cultivation period (Figure S67). In *Streptomyces* sp. 127Q, epoxide-containing trioxacarcins arose only in trace amounts, indicating transient production and fast, potentially enzymatically catalyzed, ring opening. The alteration of the cultivation conditions, such as changing the growth medium or the age of the preculture used for inoculation, did not lead to the accumulation of epoxide-containing trioxacarcins. In addition, several trioxacarcins with modifications in the aromatic core (O-methylation) and sugar moieties (reduction of the oxo moiety of α-L-trioxacarcinose B (**5**) and axenose (**17**), rather than α-L-trioxacarcinose A (**6**) moiety) were also observed. Interestingly, trioxacarcin B (**4**) was only detected transiently. Trioxacarcin B (**4**) accumulated around day 7 and increased at the midpoint of the growth phase, but it was no longer detectable after day 14, suggesting its conversion.

### 3.4. Trioxacarcin Biosynthetic Gene Cluster in Streptomyces sp. 127Q

**Figure 3:**
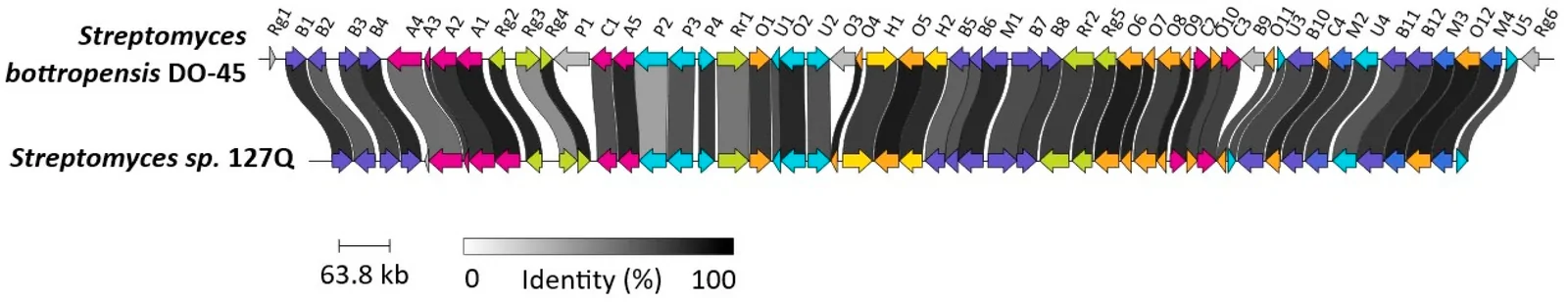
Trioxacarcin BGC from *Streptomyces* sp. 127Q in comparison to the established gene cluster from *S. bottropensis* DO-45 [73] using clinker [64]. Color coding indicates genes for the PKS and its associated enzymes (pink), oxidation-reduction enzymes (orange), deoxy sugar biosynthetic enzymes (purple), epoxide hydrolases (yellow), regulators and resistance proteins (green), DNA-alkylating enzymes and unknown-function enzymes (blue). Genes absent in *Streptomyces* sp. 127Q or *S. bottropensis* DO-45 are highlighted in grey.

The trioxacarcin BGC in *Streptomyces* sp. 127Q (accession nr.: PZ363022, Table S4) shares 80% sequence homology with the trioxacarcin BGC from *Streptomyces bottropensis* DO-45 (Figure 3, accession nr.: KP410250) [73], and 70% homology with the BGC of *Streptomyces vinaceusdrappus* NRPL 15735 (accession nr.: CP104697) [78]. This cluster codes for the biosynthesis of trioxacarcin LL-D49197α1 and trioxacarcin LL-D49197β. These differ in their glycosylation pattern [79]. Furthermore, the trioxacarcin BGC of *Streptomyces* sp. 127Q shares 84% and 76% homology with the BGCs of two other putative trioxacarcin producers: *Streptomyces grisoviridies* strain F1-27 (accession nr.: CP034687) and *Streptomyces tendae* strain VITAKN (accession nr.: JAAIFS000000000) [80] (Figure S68).

The trioxacarcin BGC from *S. bottropensis* DO-45 [73] comprises 56 open reading frames and is flanked by the pathway-specific regulators *txn*Rg1 and *txn*Rg6, which define its boundaries [73]. In contrast, the trioxacarcin BGC of *Streptomyces* sp. 127Q contains only 52 open reading frames. Compared to the BGC of *S. bottropensis* DO-45 [73], the BGC of *Streptomyces* sp. 127Q lacks five genes (*txn*Rg1, *txn*P1, *txn*Rg6, *txn*O3, and *txn*B9), but comprises one additional gene of unknown function (GM001785) (Figure 3). Similarly to the BGC of *Streptomyces* sp. 127Q, the BGCs of *Streptomyces vinaceusdrappu*s NRPL 15735 [78], *Streptomyces grisoviridies* strain F1 and *Streptomyces tendae* strain VITAKN [80] lack these genes, and can have additional genes compared to the BGC of *S. bottropensis* DO-45 (Figure S68).

*Txn*Rg1 and *txn*Rg6 constitute regulatory genes, which are only present in the BGC of *S. bottropensis* DO-45 [73]. The *txn*P1 gene [73] is annotated as an ATP-dependent CoA synthetase. The *txn*O3 gene [73] codes for a ferredoxin reductase. The *txn*B9 gene [73] codes for a glycosyltransferase, and is therefore expected to be involved in the attachment of the trioxacarcinose sugars.

### 3.5. Antimicrobial Activity of Trioxacarcins

**Figure 4:**
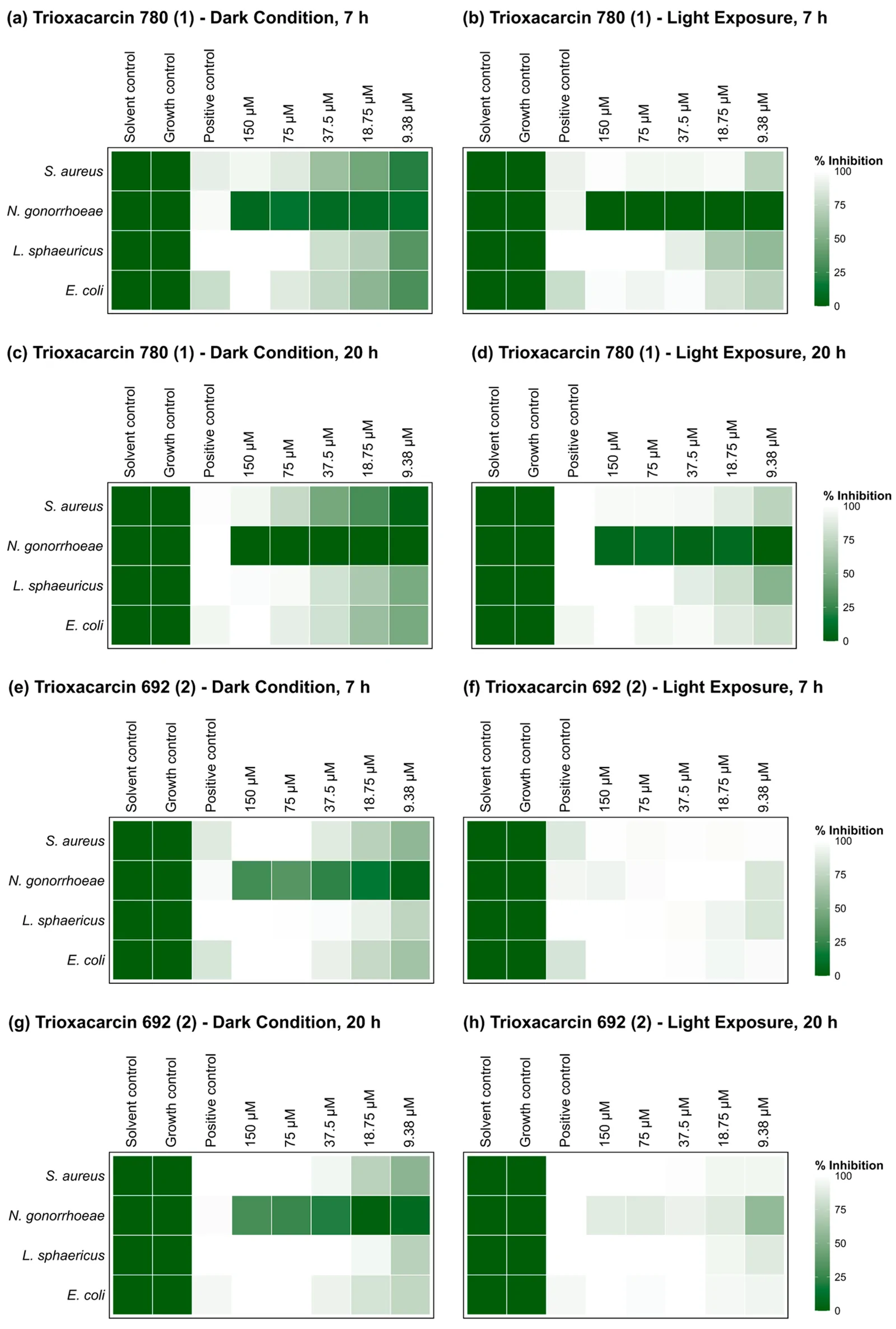
The antibacterial activity of trioxacarcin 780 (**1**) and trioxacarcin 692 (**2**) was tested against the human pathogens *E. coli*, *S. aureus*, *N. gonorrhoeae* and the stingless bee pathogen *L. sphaericus*. Bacterial growth was measured after 7 h and 20 h in a 96-well plate. The antibiotic activity of **1** and **2** was determined under dark conditions [(**a**,**c**,**e**,**g**)] and after light exposure (λ = 465 nm + 620 nm, 60 min) [(**b**,**d**,**f**,**h**)]. As positive controls, vancomycin or ciprofloxacin (9.38 μM) were used. Percentage growth inhibition was calculated relative to the 1% DMSO solvent control. The heatmap color scale represents the percentage of inhibition, ranging from 0% (dark green, no inhibition) to 100% (white, complete inhibition). The results represent the mean of three independent replicates (*n* = 3).

Due to the altered structures of trioxacarcin 780 (**1**) and trioxacarcin 692 (**2**), compared to the established trioxacarcins and gutingimycins, investigating their antimicrobial potential was of interest. In a preliminary screening, trioxacarcin 780 (**1**) and trioxacarcin 692 (**2**) were tested against a set of human pathogens, clinical isolates (*E. coli*, *S. aureus*, *S. epidermidis*, *K. pneumoniae*, *N. gonorrhoeae*, and *M. catarrhalis*) as well as the bee pathogens *P. alvei* and *L. sphaericus*. As natural products with an anthraquinone structure have been found to exhibit pronounced antimicrobial activity upon light activation (λ = 428–468 nm, 1 h) [69,81], photoantimicrobial tests were performed in parallel. The preliminary screening (Figures S69 and S70) revealed that **1** and **2** exhibited activity against *E. coli*, *S. aureus*, and *L. sphaericus* in both dark and light conditions. Moreover, the antimicrobial activity of trioxacarcin 692 (**2**) was observed against *N. gonorrhoeae*, but only after light pretreatment. As *E. coli*, *S. aureus*, *L. sphaericus* and *N. gonorrhoeae* were inhibited by both trioxacarcin 780 (**1**) and trioxacarcin 692 (**2**), they were selected to estimate the MIC of **1** and **2** against these microorganisms (Figure 4 and Figures S71–S78).

Generally, trioxacarcin 692 (**2**) exhibited greater activity than trioxacarcin 780 (**1**) against the selected microorganisms (Figure 4 and Figures S71–S78). In the dark conditions, trioxacarcin 692 (**2**) inhibited the growth of *E. coli* and *S. aureus* at the MIC of 75 µM (Figure 4, Figures S75a,c and S76a,c), while for trioxacarcin 780 (**1**), the MIC was at 150 µM (Figure 4, Figures S71a,c and S72a,c) against the same microorganisms.

Light pretreatment enhanced the antimicrobial activity of both trioxacarcins **1** and **2** against the two *E. coli* and *S. aureus* strains. Trioxacarcin 780 (**1**) exhibited a ca. 4-fold increase in activity against both strains (37.5 µM) (Figure 4, Figures S71b,d and S72b,d), while trioxacarcin 692 (**2**) exhibited a ca. 8-fold increase in activity (9.4 µM) (Figure 4, Figures S75b,d and S76b,d). The MIC of trioxacarcin 692 (**2**) may be slightly lower than the lowest tested concentration (9.4 µM). After light pretreatment, trioxacarcin 692 (**2**) at 9.4 μM inhibited the growth of *E. coli* and *S. aureus* similarly to the clinically used ciprofloxacin [82] and vancomycin [83], respectively (Figure 4, Figures S75b,d and S76b,d).

Furthermore, both trioxacarcins exhibited inhibitory activity against *L. sphaericus*, both with and without light pre-treatment, with corresponding MICs of 37.5 μM for trioxacarcin 692 (**2**) and 75 μM for trioxacarcin 780 (**1**) (Figure 4, Figures S74 and S78). Additionally, pronounced photoantimicrobial activity was observed for trioxacarcin 692 (**2**) against *N. gonorrhoeae* at the MIC of 18.75 μM (Figure 4 and Figure S77b,d), corresponding to approximately 93% inhibition, which decreased after 20 h. However, it was almost completely inactive in the dark condition (Figure S77a,c). In contrast, trioxacarcin 780 (**1**) did not inhibit *N. gonorrhoeae* in any tested conditions (Figure 4 and Figure S73).

In order to assess whether trioxacarcin 780 (**1**) and trioxacarcin 692 (**2**) permanently inhibit the growth of the test organisms, the mciroorganisms’ growth was monitored after both 7 h and 20 h of incubation.

For trioxacarcin 692 (**2**), which was tested against *E. coli*, *S. aureus*, and *N. gonorrhoeae* in the dark, the inhibitory effect remained stable, with only slight decreases or increases in activity after 20 h (Figures S75–S77). After 20 h, trioxacarcin 692 (**2**), when tested in the dark condition, inhibited *L. sphaericus* much less effectively at the lowest concentration tested (Figure S78). Following light exposure, trioxacarcin 692 (**2**) exhibited a similar growth inhibition of *E. coli*, *S. aureus*, and *L. sphaericus* after 20 h as after 7 h (Figures S75, S76 and S78). However, *N. gonorrhoeae* appeared to be significantly less inhibited by trioxacarcin 692 (**2**) after 20 h of light exposure (Figure S77).

For the less active trioxacarcin 780 (**1**), growth inhibition tends to decrease after 20 h compared to 7 h under light and dark conditions (Figures S71–S74). The growth inhibition of *E. coli* by trioxacarcin 780 (**1**) remained stable under light conditions (Figure S71).

These results suggest that trioxacarcins **1** and **2** exhibit specific antimicrobial activities against various microorganisms: *E. coli*, *S. aureus*, *N. gonorrhoeae*, and *L. sphaericus*.

## 4. Discussion

A large variety of novel trioxocarcin and gutingimycin derivatives were identified from *Streptomyces* sp. 127Q, which was isolated from the stingless bee *T. carbonaria*. 1,2-Dihydroxyanthraquinone trioxacarcins, such as trioxacarcin 780 (**1**) and trioxacarcin 692 (**2**), differ from common trioxacarcins by their tricyclic aromatic aglycon (Figure 2). Pröpper et al. observed the 1,2-dihydroxyanthraquinone gutingimycin 808 (**9**) when incubating synthetic trioxacarcin A (**3**) with the self-complementary duplex oligonucleotide d(CGTATACG), and suggested its formation by base catalysis and oxidation [77].

Trioxacarcins are known for their highly reactive epoxide moiety, which irreversibly reacts with guanine residues to form DNA adducts [84,85], as observed with trioxacarcin A (**3**), which leads to gutingimycin A (**7**) [55,77,84]. Consequently, trioxacarcin epoxides exhibit promising anti-cancer activity [54,71,86]. In addition, trioxacarcin epoxides are highly cytotoxic and exhibit antibacterial, antitumor, and antimalarial activities [71].

Although trioxacarcin 780 (**1**) and trioxacarcin 692 (**2**) lack the epoxide moiety, they were found to inhibit the stingless bee pathogen *L. sphaericus*, and human pathogens. As a wide range of pathogens is increasingly threatening pollinators [43,44,45], identifying antimicrobial compounds against bee pathogens is of great interest. As *T. carbonaria* has so far proven robust against most bee pathogens [47], it is interesting to investigate the underlying factors of its resilience. Moreover, *T. carbonaria* constitutes a potential source of microorganisms producing powerful antimicrobial compounds. The presence of microorganisms such as *Streptomyces* sp. 127Q that produce potent antibiotics, such as trioxacarcins, could support *T. carbonaria* against microbial pathogens. However, it remains unclear whether *Streptomyces* sp. 127Q is a widespread mutualistic symbiont of *T. carbonaria* that plays a defensive role in the natural context by producing trioxacarcins, for example. *Streptomyces* sp. 127Q has only been isolated from *T. carbonaria* bees from one beehive by us. Nevertheless, trioxacarcin 780 (**1**) and trioxacarcin 692 (**2**) inhibited *L. sphaericus*, the only established microbial pathogen that affects *T. carbonaria* [47]. Therefore, such antimicrobials would be advantageous for *T. carbonaria*.

Moreover, trioxacarcin 780 (**1**) and trioxacarcin 692 (**2**) were found to inhibit human pathogens, particularly *E. coli* and *S. aureus*. The activity of **1** and **2** falls within the range of gutingimycin A (**7**) [55,84]. However, when trioxacarcin 780 (**1**) and trioxacarcin 692 (**2**) were pretreated with light, similarly to other anthraquinone natural products [69,81,87,88], their antimicrobial activity was strongly enhanced by ca. 4–8 fold against some of the tested pathogens (*N. gonorrhoeae*, *E. coli*, and *S. aureus*). This activation by light is also potentially a relevant scenario under natural sunlight conditions [88]. *N. gonorrhoeae* was only inhibited by trioxacarcin 692 (**2**) after exposure to light. The results indicate antibiotic selectivity and imply multiple modes of action against different microorganisms. The 1,2-dihydroxyanthraquinone trioxacarcins lack the epoxide moiety that reacts covalently with targets, such as DNA, leading to the production of gutingimycins [55,84]. Therefore, trioxacarcin 780 (**1**) and trioxacarcin 692 (**2**) need to exhibit a different mode of action, in which the anthraquinone structure may play an important role. The pronounced activity after light exposure arises because anthraquinones promote reactive oxygen species (ROS) formation, such as hydroxyl radical and superoxide radical formation, thereby causing cell death. This mode of action is well documented for related anthraquinones, such as alizarin (1,2-dihydroxyanthraquinone) [81], and is therefore also suspected to be crucial for the activity of **1** and **2** after light exposure. Further experiments are needed to study the mode of action of 1,2-dihydroxyanthraquinone trioxacarcins in detail.

The superior antimicrobial activity of trioxacarcin 692 (**2**) compared to trioxacarcin 780 (**1**) (Figure 4 and Figures S75–S78) suggests that structural variation within the trioxacarcin family can increase the antimicrobial spectrum against pathogens. The large diversity of trioxacarcin products may provide tailored antimicrobials against a variety of potential threats, similar to previous observations of the diversity of piericidins in the beewolf [38]. Moreover, a single microorganism has the genetic capacity to form multiple antimicrobial compounds, despite the fact that many microorganisms are usually associated with insects. *P. alvei* was not inhibited by trioxacarcin 780 (**1**) and trioxacarcin 692 (**2**) (Figures S69 and S70), but the crude ethyl acetate extract inhibited its growth (Figure S1), indicating that either other trioxacarcin/gutingimycin variants, or another, as yet unidentified, secondary metabolite from *Streptomyces* sp. 127Q may be responsible for activity against *P. alvei*.

Remarkably, we did not detect the accumulation of trioxacarcin epoxides during the growth of *Streptomyces* sp. 127Q, despite our purification protocol being similar to that used for the isolation of trioxacarcins [53,71]. This contrasts with the identified trioxacarcins from *S. bottropensis* [53,71,73] When we analyzed the supernatant directly by LC-MS, without a workup, which could potentially cause epoxide hydrolysis, the epoxides were not detected. However, the usual trioxacarcin profile was observed, including trioxacarcin 780 (**1**), trioxacarcin 692 (**2**), and gutingimycin (**7**) from *Streptomyces* sp. 127Q. This strongly suggests that the trioxacarcin epoxides undergo rapid hydrolysis by *Streptomyces* sp. 127Q, which is potentially catalyzed by cluster-encoded epoxide hydrolases, GM001804 (*txn*H1) and GM001806 (*txn*H2) [73]. Clearly, trioxacarcin epoxides are the precursors of all trioxacarcin and gutingimycin products. Our time course experiment following the formation of trioxacarcin/gutingimycins by *Streptomyces* sp. 127Q revealed that gutingimycin A (**7**) (Figure S67) appeared as early as day four, with additional gutingimycin metabolites (**10**–**16**) appearing from day seven onwards.

It would be of particular interest to combine the epoxide DNA-binding activity [77,84,85,89] and light-inducible activity of the 1,2-dihydroxyanthraquinones in a trioxacarcin, in order to test its potential against both pathogens and cancer. Under suitable conditions, this combined activity of epoxide and ROS formation potentially occurs, as observed by the reaction of trioxacarcin A (**3**) and the oligonucleotide d(CGTATACG), as reported by Pröpper et al. [77]. This results in the loss of the α-L-trioxacarcinose A (**6**) sugar moiety, which acts as a protection group, setting off this second reactive site in addition to the epoxide moiety. In this context, the photochemical induction activity of 1,2-dihydroxyanthraquinones is of interest in drug design for the selective targeting of cancer cells [90].

1,2-dihydroxyanthraquinone trioxacarcin epoxides would be accessible by total synthesis, by modifying the established synthetic routes to trioxacarcins [72,91,92]. Alternatively, engineering the trioxacarcin biosynthetic gene cluster is a possible route to obtaining trioxacarcins with both epoxide and 1,2-dihydroxyanthraquinone moiety.

That *Streptomyces* sp. 127Q produced trioxacarcin epoxide reaction products with nicotinic acid is remarkable, because until now, only epoxide hydrolysis [54,71] or reaction with guanine [84] from DNA has been seen. Secondary metabolites containing a nicotinic acid or nicotinamide moiety are rather rare; examples include the NRPS-produced myxochelins N1-N3 from *Corallococcus* sp. MCy9049 [93], kosinostatins from *Micromonospora* sp. [94], the fungal meroterpenoid, pyripyropene [95], and the asperphenamates from *Penicillum astrolabium* [96].

In *Streptomyces* sp. 127Q, trioxacarcin epoxides reacted readily with either nicotinic acid or a nicotinic acid-containing metabolite. The reaction was not limited to the presence of nicotinic acid in the growth medium, although the R5A medium, which is rich in nicotinic acid due to the presence of yeast extract, yielded trioxacarcin nicotinic acid esters in the best yield. *Streptomyces* sp. 127Q can evidently produce nicotinic acid esters of trioxacarcins independently of nicotinic acid. However, no genes coding for nicotinic acid production were detected within the trioxacarcin BGC of *Streptomyces* sp. 127Q or in its environment. The formation of nicotinic acid esters potentially comprises a resistance mechanism against trioxacarcin epoxides in *Streptomyces* sp. 127Q, or it may serve to diversify the trioxacarcin family. However, in our antimicrobial tests, trioxacarcin 780 (**1**) was less active than the hydrolyzed epoxide, trioxacarcin 692 (**2**). The selectivity of trioxacarcin 1,2-dihydroxyanthraquinone epoxide for nicotinic acid would be an interesting subject to study. The reaction of the trioxacarcin epoxides with nicotinic acid could potentially constitute an additional mechanism of action, weakening the fitness of other organisms by depriving them of nicotinic acid, which is needed for NAD(P)H and serves as a vitamin for many organisms [97].

Trioxacarcins were produced by *Streptomyces* sp. 127Q only in R5A and R2 media, containing a high concentration of sucrose (103 g/L). Other trioxacarcin producers form them in much less sugar-rich media [52,58]. This may reflect the adaptation of *Streptomyces* sp. 127Q to a sugar-rich nutrient supply from its bee host, *T. carbonaria*. In contrast, *S. bottropensis* DO-45 produced the highest levels of trioxacarcins in the presence of soluble starch, dextrin, and fructose, whereas glucose resulted in significantly lower levels of production. Sucrose (20 g/L) only moderately supported trioxacarcin production by *S. bottropensis* DO-45 [52]. Differences in the regulation of the trioxacarcin biosynthetic gene cluster (*txn*Rg1 and *txn*Rg6) potentially explain the variation in trioxacarcin production in response to different levels of nutrient availability. Moreover, regulatory differences may influence product yield, alter expression timing, or cause alterations in product ratios.

The production of both the known trioxacarcins/gutingimycins and 1,2-hydroxyanthraquinone variants by *Streptomyces* sp. 127Q is suspected to be caused by differences in the trioxacarcin BGC of *Streptomyces* sp. 127Q compared to that of the well-studied BGC from the trioxacarcin producer *S. bottropensis* DO-45 (Figure 4 and Figure S68) [73,98]. The key differences lie in missing/additional genes, changes in the gene cluster arrangement, and differing gene sequences, which could affect trioxacarcins production. The *txn*A4 gene, which codes for an enzyme involved in the polyketide formation, has an altered *N*-terminal sequence compared to the *txn*A4 gene from *S. bottropensis* DO-45. This could alter ACP docking and thus enzyme activity and PKS intermediate formation [99,100]. Moreover, the ferredoxin reductase *txn*O3, which is suspected to be involved in the oxidative tailoring of the polyketide core to form trioxacarcins [73], is missing in *Streptomyces* sp. 127Q, and may potentially influence the different product formation by *Streptomyces* sp. 127Q compared to *S. bottropensis* DO-45.

**Figure 5:**
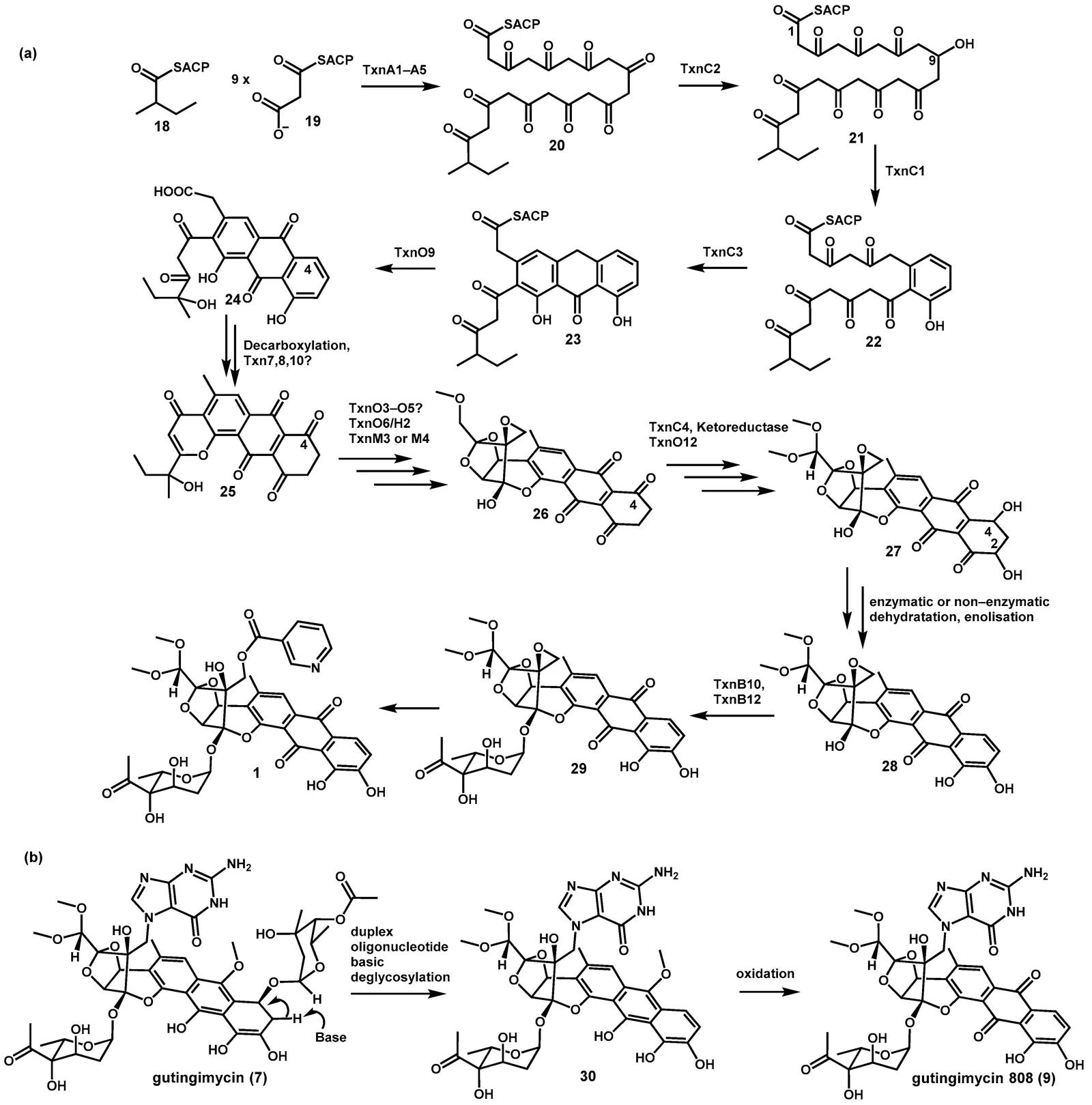
Possibilities affecting the formation of 1,2-dihydroxyanthraquinone trioxacarcin/gutingimycin: (**a**) Proposed variation in the trioxacarcin biosynthesis to produce trioxacarcins with 1,2-dihydroxyanthraquinone aglycon, such as trioxacarcin 780 (**1**). (**b**) Observed reaction of synthetic trioxacarcin A with duplex oligonucleotide d(CGTATACG) to gutingimycin 808 (**9**), with the suggested duplex oligonucleotide base-catalyzed deglycolysation and formation of the 1,2-dihydroxyanthraquinone gutingimycin (**9**) during crystallization for X-ray analysis by Pröpper et al. [77].

Most obviously, *Streptomyces* sp. 127Q lacks the glycosyltransferase TxnB9 compared to *S. bottropensis* DO-45, which is suspected to be involved in the attachment of the trioxacarcinose A (**6**) to the aglycon at carbon C4 of the intermediate **27** in the biosynthetic pathway (Figure 5) [73]. It has previously been observed that glycosyltransferases can require other enzymes to function as helpers. However, in the case of EryCII/EryCIII, the helper EryCIII is instead a P450-like enzyme [101]. As *Streptomyces* sp. 127Q forms trioxacarcins/gutingimycins with α-L-trioxacarcinose A (**6**) moieties, the other two glycosyltransferases, TxnB10 and TxnB12, may be sufficient to add both trioxacarcinose sugar moieties, but probably with reduced efficiency.

Additionally, *Streptomyces* sp. 127Q lacks the *txn*P1 gene, which codes for an ATP-dependent CoA synthetase. The substrate that this enzyme activates is unclear; potentially, other CoA synthetases can take over this function. This alteration may therefore lead to differences in the kinetics of product formation, and thus indirectly influence the product spectrum.

Due to the X-ray experiments conducted by Pröpper et al. [77], which led to the conversion of trioxacarcin A (**7**) to 1,2-dihydroxyanthraquinone gutingimycin 808 (**9**), one could speculate whether the formation of 1,2-dihydroxyanthraquinone trioxacarcins and gutingimycins is due to the pH conditions present during growth and/or workup. However, we isolated the compounds from the spent medium at the native pH of 6.8. Moreover, the 1,2-dihydroxyanthraquinone trioxacarcins and gutingimycins occur in similar amounts to the established trioxacarcins and gutingimycins, and have a characteristic UV spectrum and similar retention time. Therefore, it is highly unlikely that they would have escaped previous isolations by other research groups studying trioxacarcins/gutingimycins from *S. bottropensis* strains [53,55,71,73]. The direct LC-MS analysis of the spent medium supernatants of *Streptomyces* sp. 127Q revealed the presence of 1,2-dihydroxyanthraquinone trioxacarcins and gutingimycins identical to worked-up samples, indicating that these compounds are not produced during the workup process.

Moreover, the long cultivation of *Streptomyces* sp. 127Q for two months did not lead to a pronounced amount of gutingimycin 808 (**9**) as a breakdown product of gutingimycin A (**7**), but rather its precursor gutingimycin A (**7**) was a major product (ca. 1:9). 1,2-Dihydroxyanthraquinone trioxacarcins and gutingimycins are present in similar amounts alongside established trioxacarcins at early time points (e.g., trioxacarcin 780 (**1**): gutingimycin A (**7**) ca. 3:1, Figure S67), suggesting a conversion due to the altered gene cluster.

In the experiment by Pröpper et al., we suspect that a basic moiety of the duplex oligonucleotide d(CGTATACG) specifically catalyzed the cleavage of α-L-trioxacarcinose A (**6**) from trioxacarcin A (**3**). Moreover, it reacted with the oligonucleotide by epoxide opening to yield the 1,2-dihydroxyanthraquinone gutingimycin 808 (**9**) [77]. Future experiments should study this potential specific reaction by using an appropriate oligonucleotide. Other studies investigating the reaction of trioxacarcin A (**3**) with DNA using other oligonucleotides did not observe the formation of the 1,2-dihydroxyanthraquinone gutingimycin 808 (**9**) [84,85,89]. The basic hydrolysis of glycosides by deprotonation of β-substituted alcohol glycosides is well established [102,103,104]. If the 4-hydroxy moiety of the trioxacarcin aglycon is unprotected, either by the loss of α-L-trioxacarcinose A (**6**) sugar moiety or by the alteration of the biosynthesis, missing glycosylation, the aglycon will react to the 1,2-dihydroxyanthraquinone moiety (Figure 5). The 1,2-dihydroxyanthraquinone moiety potentially constitutes a protection group in trioxacarcin A (**3**), which can be released upon binding to DNA, creating a second active site. However, it is unclear whether 1,2-dihydroxyanthraquinones are formed by modifications of biosynthesis, or non-enzymatically due to the physiological conditions during the growth of *Streptomyces* sp. 127Q.

Apart from the potential of *Streptomyces* sp. 127Q to form 1,2-dihydroxy-anthraquinone trioxacarcins, it produces a variety of trioxacarcins with different methylation patterns introduced to the aglycons (**13**, **14**). This suggests that the methyltransferases can act on different hydroxy groups of the trioxacarcin aglycons, leading to further product diversity.

Further experiments are needed to clarify whether *Streptomyces* sp. 127Q forms the 1,2-dihydroxyanthraquinone trioxacarcins via the proposed pathway (Figure 5a), and which differences in the trioxacarcin BGC of *Streptomyces* sp. 127Q would be responsible for the altered product formation compared to *S. bottropensis* DO-45 [73].

For example, the suspected role of the glycosyltransferase *txn*B9 should be studied by introducing the relevant genes into *Streptomyces* sp. 127Q and analyzing the resulting trioxacarcin products. Further trioxacarcin variants from *Streptomyces* sp. 127Q and from strains containing the putative trioxacarcin gene clusters should be identified and screened for their bioactivity (Figure S61). Moreover, nature effectively demonstrates the potential to create new bioactive secondary metabolites, which could inspire the engineering of the trioxacarcin BGC to produce new bioactive products. The pharmaceutical potential of new trioxacarcin compounds compared to established ones should also be addressed.

## 5. Conclusions

Microorganisms isolated from insects such as the Australian stingless bee *T. carbonaria* are a promising source for powerful, novel antimicrobial compounds. These antimicrobial compounds could potentially support the insect host against microbial infections, and may provide protection against pathogens affecting honeybees and humans. *Streptomyces* sp. 127Q produces a large variety of trioxacarcins, gutingimycins, and trioxacarcin 1,2-dihydroxyanthraquinones. The epoxide precursors of these compounds are considered to be highly reactive, because they contain both the reactive epoxide and the ROS-inducing anthraquinone moiety. Nevertheless, trioxacarcin epoxide reaction products with water or nicotinic acid, such as trioxacarcin 692 (**2**) and trioxacarcin 780 (**1**), strongly inhibit the growth of *E. coli* and *S. aureus*. Potentially, by making use of a few changes to its BGC compared to the BGC of *S. bottropensis* DO-45 [73], *Streptomyces* sp. 127Q generates a variety of previously unknown antimicrobial trioxacarcins/gutingimycins, which may constitute an efficient strategy against diverse potential microbial competitors.

## Data Availability

The data presented in this study are available in Supporting Information and from the authors.

## Supplementary Materials

The following supporting information can be downloaded at https://www.mdpi.com/article/10.3390/biom16081159/s1: Figure S1. Antibacterial activity of ethyl acetate extract from *Streptomyces* sp. 127Q against (a) *L. sphaericus* and (b) *P. alvei*; Figure S2. UV-Vis spectrum of trioxacarcin 780 (**1**); Figure S3. HR-ESI-MS of trioxacarcin 780 (**1**); Figure S4. HR-ESI-MS/MS of the quasimolecular ion at *m*/*z* 780 of trioxacarcin 780 (**1**); Figure S5. ^1^H NMR spectrum (800 MHz, CD_3_OD/CDCl_3_/DCl) of trioxacarcin 780 (**1**); Figure S6. ^1^H NMR spectrum (800 MHz, CD_3_OD/CDCl_3_/DCl) of trioxacarcin 780 (**1**) aromatic region; Figure S7. ^1^H NMR spectrum (800 MHz, CD_3_OD/CDCl_3_/DCl) of trioxacarcin 780 (**1**) 4.4–5.5 region; Figure S8. ^1^H NMR spectrum (800 MHz, CD_3_OD/CDCl_3_/DCl) of trioxacarcin 780 (**1**) 1.6–2.5 region; Figure S9. ^13^C NMR spectrum (800 MHz, CD_3_OD/CDCl_3_/DCl) of trioxacarcin 780 (**1**); Figure S10. ^1^H−^1^H COSY NMR spectrum (800 MHz, CD_3_OD/CDCl_3_/DCl) of trioxacarcin 780 (**1**); Figure S11. ^1^H−^13^C-HSQC NMR spectrum (800 MHz, CD_3_OD/CDCl_3_/DCl) of trioxacarcin 780 (**1**); Figure S12. ^1^H−^13^C HMBC NMR spectrum (800 MHz, CD_3_OD/CDCl_3_/DCl) of trioxacarcin 780 (**1**); Figure S13. ^1^H−^1^H-ROESY NMR spectrum (800 MHz, CD_3_OD/CDCl_3_/DCl) of trioxacarcin 780 (**1**); Figure S14. Key (a) ^1^H−^1^H COSY and (b) ^1^H−^13^C HMBC correlations of trioxacarcin 780 (**1**); Figure S15. UV-Vis spectrum of trioxacarcin 608 (**8**); Figure S16. HR-ESI-MS of trioxacarcin 608 (**8**); Figure S17. HR-ESI-MS/MS fragmentation of trioxacarcin 608 (**8**); Figure S18. Detection of nicotinic acid (*m*/*z* 124) after the hydrolysis of trioxacarcin 780 (**1**); Figure S19. UV-Vis spectrum of gutingimycin A (**7**); Figure S20. HR-ESI-MS of gutingimycin A (**7**); Figure S21. HR-ESI-MS/MS fragmentation of gutingimycin A (**7**); Figure S22. ^1^H NMR spectrum of gutingimycin A (**7**) (800 MHz, CDCl_3_); Figure S23. ^13^C NMR spectrum of gutingimycin A (**7**) (201 MHz, CDCl_3_); Figure S24. ^1^H−^1^H-COSY NMR spectrum of gutingimycin A (**7**) (800 MHz, CDCl_3_); Figure S25. ^1^H−^13^C-HSQC NMR spectrum of gutingimycin A (**7**) (800 MHz, CDCl_3_); Figure S26. ^1^H−^13^C-HMBC NMR spectrum of gutingimycin A (**7**) (800 MHz, CDCl_3_); Figure S27. ^1^H−^1^H-ROESY NMR spectrum of gutingimycin A (**7**) (800 MHz, CDCl_3_); Figure S28. UV-Vis spectrum of trioxacarcin 692 (**2**); Figure S29. HR-ESI-MS of trioxacarcin 692 (**2**); Figure S30. HR-ESI-MS/MS fragmentation of trioxacarcin 692 (**2**); Figure S31. ^1^H NMR spectrum (800 MHz, CD_3_OD/CDCl_3_) of trioxacarcin 692 (**2**); Figure S32. ^1^H NMR spectrum (800 MHz, CD_3_OD/CDCl_3_) of trioxacarcin 692 (**2**), aromatic region; Figure S33. ^1^H NMR spectrum (800 MHz, CD_3_OD/CDCl_3_) of trioxacarcin 692 (**2**), 4.5–5.6 ppm; Figure S34. ^1^H NMR spectrum (800 MHz, CD_3_OD/CDCl_3_) of trioxacarcin 692 (**2**), 1.7–3.8 ppm; Figure S35. ^1^H−^1^H COSY NMR spectrum (800 MHz, CD_3_OD/CDCl_3_) of trioxacarcin 692 (**2**); Figure S36. ^1^H−^13^C-HSQC NMR spectrum (800 MHz, CD_3_OD/CDCl_3_) of trioxacarcin 692 (**2**); Figure S37. ^1^H−^13^C HMBC NMR spectrum (800 MHz, CD_3_OD/CDCl_3_) of trioxacarcin 692 (**2**); Figure S38. Key (a) ^1^H−^1^H COSY and (b) ^1^H−^13^C HMBC correlations of trioxacarcin 692 (**2**); Figure S39. Detection of trioxacarcin 780 (**1**) in the processed supernatant of 21 *d Streptomyces* sp. 127Q culture; Figure S40. Relative production of trioxacarcin and gutingimycin compounds by *Streptomyces* sp. 127Q; Figure S41. Molecular networking of trioxacarcins and gutingimycins detected in Diaion HP20 eluate, prepared from 4 L of 21 d old *Streptomyces* sp. 127Q culture; Figure S42. UV-Vis spectrum of gutingimycin 808 (**9**); Figure S43. HR-ESI-MS of trioxacarcin 808 (**9**); Figure S44. HR-ESI-MS/MS fragmentation of gutingimycin 808 (**9**); Figure S45. UV-Vis spectrum of gutingimycin B (**10**); Figure S46. HR-ESI-MS of gutingimycin B (**10**); Figure S47. HR-ESI-MS/MS fragmentation of gutingimycin B (**10**); Figure S48. UV-Vis spectrum of gutingimycin C (**11**); Figure S49. HR-ESI-MS of gutingimycin C (**11**); Figure S50. HR-ESI-MS/MS fragmentation of gutingimycin C (**11**); Figure S51. UV-Vis spectrum of gutingimycin 1042 (**12**); Figure S52. HR-ESI-MS of gutingimycin 1042 (**12**); Figure S53. HR-ESI-MS/MS fragmentation of gutingimycin 1042 (**12**); Figure S54. UV-Vis spectrum of gutingimycin 986 (**13**); Figure S55. HR-ESI-MS of trioxacarcin 986 (**13**); Figure S56. HR-ESI-MS/MS fragmentation of gutingimycin 986 (**13**); Figure S57. UV-Vis spectrum of gutingimycin 822 (**14**); Figure S58. HR-ESI-MS of trioxacarcin 822 (**14**); Figure S59. HR-ESI-MS/MS fragmentation of gutingimycin 822 (**14**); Figure S60. UV-Vis spectrum of trioxacarcin 958 (**15**); Figure S61. HR-ESI-MS of trioxacarcin 958 (**15**); Figure S62. HR-ESI-MS/MS fragmentation of trioxacarcin 958 (**15**); Figure S63. UV-Vis spectrum of trioxacarcin 1000 (**16**); Figure S64. HR-ESI-MS of trioxacarcin 1000 (**16**); Figure S65. HR-ESI-MS/MS fragmentation of trioxacarcin 1000 (**16**); Figure S66. (a) Detection of trioxacarcin 780 (**1**), and (b) trioxacarcin 692 (**2**), in the processed supernatant of 21-day old *Streptomyces* sp. 127Q culture; Figure S67. Time course of trioxacarcins and gutingimycins formation in R5A medium inoculated with precultures of different ages; Figure S68. Alignment of the trioxacarcin biosynthetic gene clusters from three trioxacarcin producers and two putative trioxacarcin producers; Figure S69. Preliminary screening of the antibacterial activity of trioxacarcin 780 (**1**) against the human pathogens *E. coli*, *S. aureus*, *N. gonorrhoeae*, *M. catarrhalis*, *K. pneumoniae*, and *S. epidermidis*, as well as the bee pathogens *L. sphaericus* and *P. alvei*; Figure S70. Preliminary screening of the antibacterial activity of trioxacarcin 692 (**2**) against the human pathogens *E. coli*, *S. aureus*, *N. gonorrhoeae*, *M. catarrhalis*, *K. pneumoniae*, and *S. epidermidis*, as well as the bee pathogens *L. sphaericus* and *P. alvei*; Figure S71. Antibacterial activity of trioxacarcin 780 (**1**) against *E. coli*; Figure S72. Antibacterial activity of trioxacarcin 780 (1) against *S. aureus*; Figure S73. Antibacterial activity of trioxacarcin 780 (**1**) against *N. gonorrhoeae*; Figure S74. Antibacterial activity of trioxacarcin 780 (**1**) against *L. sphaericus*; Figure S75. Antibacterial activity of trioxacarcin 692 (**2**) against *E. coli*; Figure S76. Antibacterial activity of trioxacarcin 692 (**2**) against *S. aureus*; Figure S77. Antibacterial activity of trioxacarcin 692 (**2**) against *N. gonorrhoeae*; Figure S78. Antibacterial activity of trioxacarcin 692 (**2**) against *L. sphaericus*; Table S1. NMR data of trioxacarcin 780 (1) (800 MHz, CD_3_OD/CDCl_3_/DCl); Table S2. NMR data of gutingimycin A (7) (800 MHz, CDCl_3_); Table S3. NMR data of trioxacarcin 692 (2) (800 MHz, CD_3_OD/CDCl_3_); Table S4. Comparison of the genes in the trioxacarcin biosynthetic gene clusters of *Streptomyces* sp. 127Q (accession nr.: PZ363022) and *Streptomyces bottropensis* DO-45 (KP410250).

## Abbreviations

The following abbreviations are used in this manuscript:

ACP	Acyl carrier protein
BGC	Biosynthetic gene cluster
^13^C NMR	Carbon nuclear magnetic resonance
COSY	Correlated spectroscopy
ESI	Electrospray ionization
HPLC	High performance liquid chromatograph/high performance liquid chromatograph
HR-MS	High resolution mass spectrometry/mass spectrometer
HMBC	Heteronuclear multiple bond correlation
^1^H NMR	Proton nuclear magnetic resonance
HSQC	Heteronuclear single quantum coherence
LC-MS	Liquid chromatograph hyphenated to mass spectrometer
LC-UV-Vis-MS	Liquid chromatograph hyphenated to UV-Vis- and mass-spectrometer
MS	Mass spectrometry/mass spectrometer
NC	negative control
NMR	Nuclear magnetic resonance
MIC	Minimal inhibitory concentration
MPLC	Medium pressure chromatography
OD	Optical density
PKS	Polyketide synthase
ROS	Reactive oxygen species
SD	Standard deviation
UV-Vis	Ultraviolet visible
