# Supplementary material for: Antimicrobial Trioxacarcin 1,2-Dihydroxyanthraquinones from the Bacterium Streptomyces sp. 127Q Isolated from the Stingless Bee Tetragonula carbonaria
Source: Biomolecules. 2026 Aug 10;16(8):1159. doi: 10.3390/biom16081159 (PMC13511009; doi:10.3390/biom16081159)
Supplement: Supplementary file 1 [file biomolecules-16-01159-s001.zip › biomolecules-4328959-supplementary.pdf]

Antimicrobial trioxacarcin 1,2-dihydroxyanthraquinones from the bacterium *Streptomyces* sp. 127Q isolated from the stingless bee *Tetragonula carbonaria*

Anastasiia Filimonova[1, 2] and Dieter Spiteller[1, 2*]

[1] Chemical Ecology/Biological Chemistry, Department of Biology, University of Konstanz, Universitätsstraße 10, 78457 Konstanz, Germany
[2] Konstanz Research School Chemical Biology, University of Konstanz, 78457 Konstanz, Germany; anastasiia.filimonova@uni-konstanz.de, dieter.spiteller@uni-konstanz.de

Table of contents

Antibacterial activity of ethyl acetate extract from *Streptomyces* sp. 127Q against *L. sphaericus* and *P. alvei* 4
Trioxacarcin 780 (1) 4
UV-Vis spectrum of trioxacarcin 780 (1) 4
HR-ESI-MS of trioxacarcin 780 (1) 5
HR-ESI-MS/MS of trioxacarcin 780 (1) 5
^{1}H NMR spectrum of trioxacarcin 780 (1) 6
^{13}C NMR spectrum of trioxacarcin 780 (1) 8
^{1}H-^{1}H-COSY NMR spectrum of trioxacarcin 780 (1) 9
^{1}H-^{13}C-HSQC NMR spectrum of trioxacarcin 780 (1) 10
^{1}H-^{13}C-HMBC NMR spectrum of trioxacarcin 780 (1) 11
^{1}H-^{1}H-ROESY NMR spectrum of trioxacarcin 780 (1) 12
Key ^{1}H-^{1}H COSY and ^{1}H-^{13}C HMBC correlations of trioxacarcin 780 (1) 13
NMR data of trioxacarcin 780 (1) 14
Trioxacarcin 608 aglycon (8) 15
UV-Vis spectrum of trioxacarcin 608 (8) 15
HR-ESI-MS of trioxacarcin 608 (8) 15
HR-ESI-MS/MS of trioxacarcin 608 (8) 16
Hydrolysis of trioxacarcin 780 (1) 17
Gutingimycin A (7) 18
UV-Vis spectrum of gutingimycin A (7) 18
HR-ESI-MS of gutingimycin A (7) 18
ESI-MS/MS of gutingimycin A (7) 19
^{1}H NMR spectrum of gutingimycin A (7) 20

^{13}C NMR spectrum of gutingimycin A (7) 21
^{1}H-^{1}H-COSY NMR spectrum of gutingimycin A (7) 22
^{1}H-^{13}C-HSQC NMR spectrum of gutingimycin A (7) 23
^{1}H-^{13}C-HMBC NMR spectrum of gutingimycin A (7) 24
^{1}H-^{1}H-ROESY spectrum of gutingimycin A (7) 25
NMR data of gutingimycin A (7) 26
Trioxacarcin 692 (2) 28
UV-Vis spectrum of trioxacarcin 692 (2) 28
HR-ESI-MS of trioxacarcin 692 (2) 28
ESI-MS/MS of trioxacarcin 692 (2) 29
^{1}H NMR spectrum of trioxacarcin 692 (2) 29
^{1}H-^{1}H-COSY NMR spectrum of trioxacarcin 692 (2) 32
^{1}H-^{13}C-HSQC NMR spectrum of trioxacarcin 692 (2) 33
^{1}H-^{13}C-HMBC NMR spectrum of trioxacarcin 692 (2) 34
Key ^{1}H-^{1}H COSY and ^{1}H-^{13}C HMBC correlations of trioxacarcin 692 (2) 34
NMR data of trioxacarcin 692 (2) 35
Production of trioxacarcin 780 (1) in modified R5A medium 36
LC-MS chromatogram of *Streptomyces* sp. 127Q crude extract: formation of trioxacarcin and gutingimycin compounds 37
Molecular networking analysis of trioxacarcin and gutingimycin compounds of *Streptomyces* sp. 127Q 38
Gutingimycin 808 (9) 39
UV-Vis spectrum of gutingimycin 808 (9) 39
HR-ESI-MS of gutingimycin 808 (9) 39
ESI-MS/MS of gutingimycin 808 (9) 40
Gutingimycin B (10) 41
UV-Vis spectrum of gutingimycin B (10) 41
HR-ESI-MS of gutingimycin B (10) 41
ESI-MS/MS of gutingimycin B (10) 42
Gutingimycin C (11) 43
UV-Vis spectrum of gutingimycin C (11) 43
HR-ESI-MS of gutingimycin C (11) 43
ESI-MS/MS of gutingimycin C (11) 44
Gutingimycin 1042 (12) 45
UV-Vis spectrum of gutingimycin 1042 (12) 45
HR-ESI-MS of gutingimycin 1042 (12) 45

ESI-MS/MS of gutingimycin 1042 (12) ... 46
Gutingimycin 986 (13) ... 47
UV-Vis spectrum of gutingimycin 986 (13) ... 47
HR-ESI-MS of gutingimycin 986 (13) ... 47
ESI-MS/MS of gutingimycin 986 (13) ... 48
Gutingimycin 822 (14) ... 49
UV-Vis spectrum of gutingimycin 822 (14) ... 49
HR-ESI-MS of gutingimycin 822 (14) ... 49
ESI-MS/MS of gutingimycin 822 (14) ... 50
Trioxacarcin 958 (15) ... 51
UV-Vis spectrum of trioxacarcin 958 (15) ... 51
HR-ESI-MS of trioxacarcin 958 (15) ... 51
ESI-MS/MS of trioxacarcin 958 (15) ... 52
Trioxacarcin 1000 (16) ... 53
UV-Vis spectrum of trioxacarcin 1000 (16) ... 53
HR-ESI-MS of trioxacarcin 1000 (16) ... 53
ESI-MS/MS of trioxacarcin 1000 (16) ... 54
Production of trioxacarcin 780 (1) and trioxacarcin 692 (2) in different production media ... 55
Time course of trioxacarcins and gutingimycins formation ... 56
Trioxacarcin biosynthetic gene cluster in *Streptomyces* sp. 127Q ... 58
Comparison of trioxacarcin biosynthetic gene clusters ... 60
Preliminary screening of the antimicrobial activity of trioxacarcin 780 (1) against human and bee pathogens ... 61
Preliminary screening of the antimicrobial activity of trioxacarcin 692 (2) against human and bee pathogens ... 62
Antibacterial activity of trioxacarcin 780 (1) against *Escherichia coli* ... 63
Antibacterial activity of trioxacarcin 780 (1) against *Staphylococcus aureus* ... 64
Antibacterial activity of trioxacarcin 780 (1) against *Neisseria gonorrhoeae* ... 65
Antibacterial activity of trioxacarcin 780 (1) against *Lysinibacillus sphaericus* ... 66
Antibacterial activity of trioxacarcin 692 (2) against *Escherichia coli* ... 67
Antibacterial activity of trioxacarcin 692 (2) against *Staphylococcus aureus* ... 68
Antibacterial activity of trioxacarcin 692 (2) against *Neisseria gonorrhoeae* ... 69
Antibacterial activity of trioxacarcin 692 (2) against *Lysinibacillus sphaericus* ... 70

Antibacterial activity of ethyl acetate extract from *Streptomyces* sp. 127Q against *L. sphaericus* and *P. alvei*

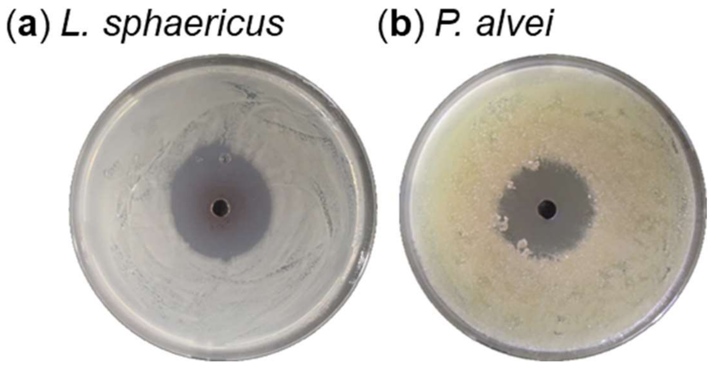

Figure S1. Antibacterial activity of ethyl acetate extract from *Streptomyces* sp. 127Q against (**a**) *L. sphaericus* and (**b**) *P. alvei.* Crude ethyl acetate extract at pH 6.8 (corresponding to approximately 0.1 g of crude extract) was tested in agar diffusion assays. The extract was prepared from a 1 l *Streptomyces* sp. 127Q culture grown for 21 d. A volume of 35-50 µl was applied per hole (corresponding to a final concentration of ca. 2.3-3.3 mg per hole).

Trioxacarcin 780 (1)

UV-Vis spectrum of trioxacarcin 780 (1)

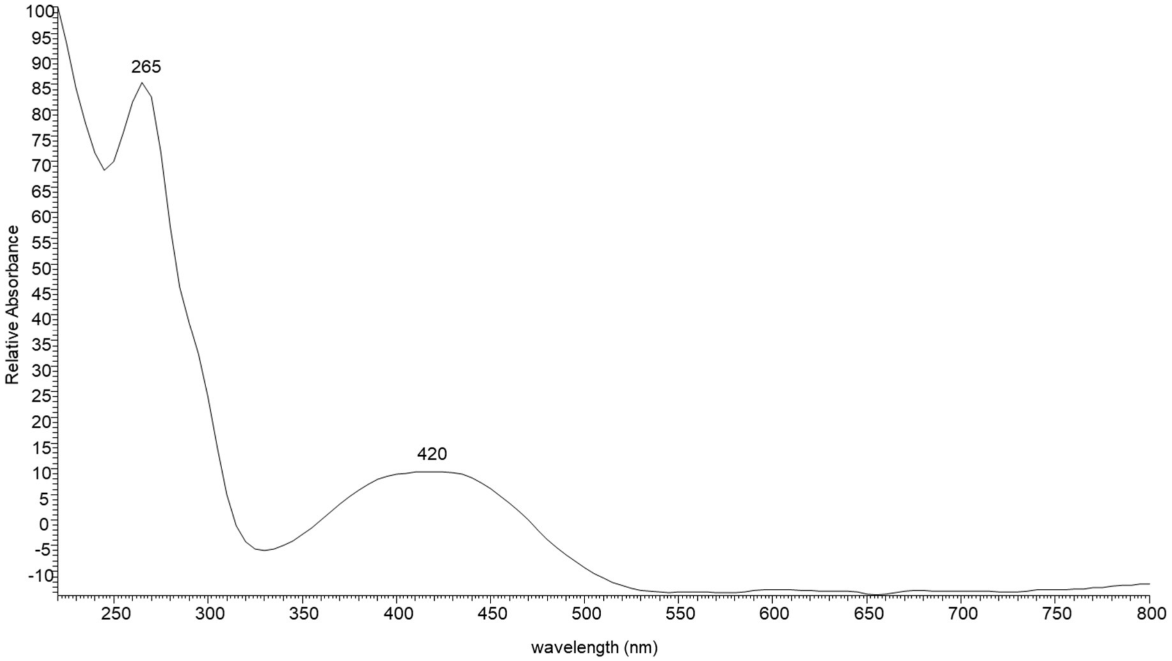

Figure S2. UV-Vis spectrum of trioxacarcin 780 (**1**).

HR-ESI-MS of trioxacarcin 780 (1)

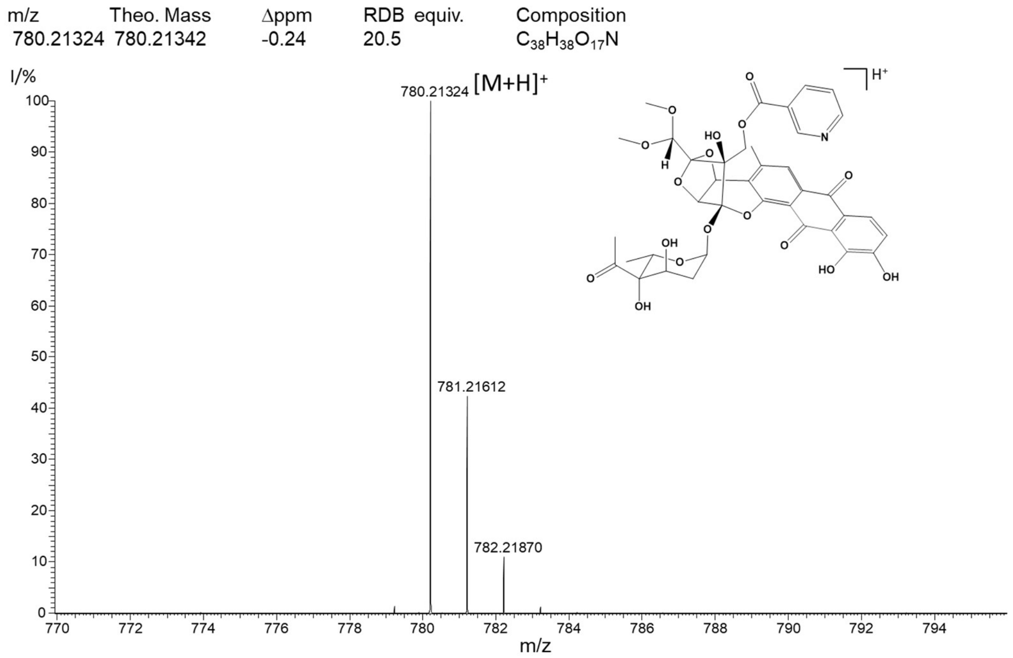

Figure S3. HR-ESI-MS of trioxacarcin 780 (**1**). Retention time 20.2 min, Macherey-Nagel RP8 column (250 × 2 mm, 5 µm), LC-MS program 1.

HR-ESI-MS/MS of trioxacarcin 780 (1)

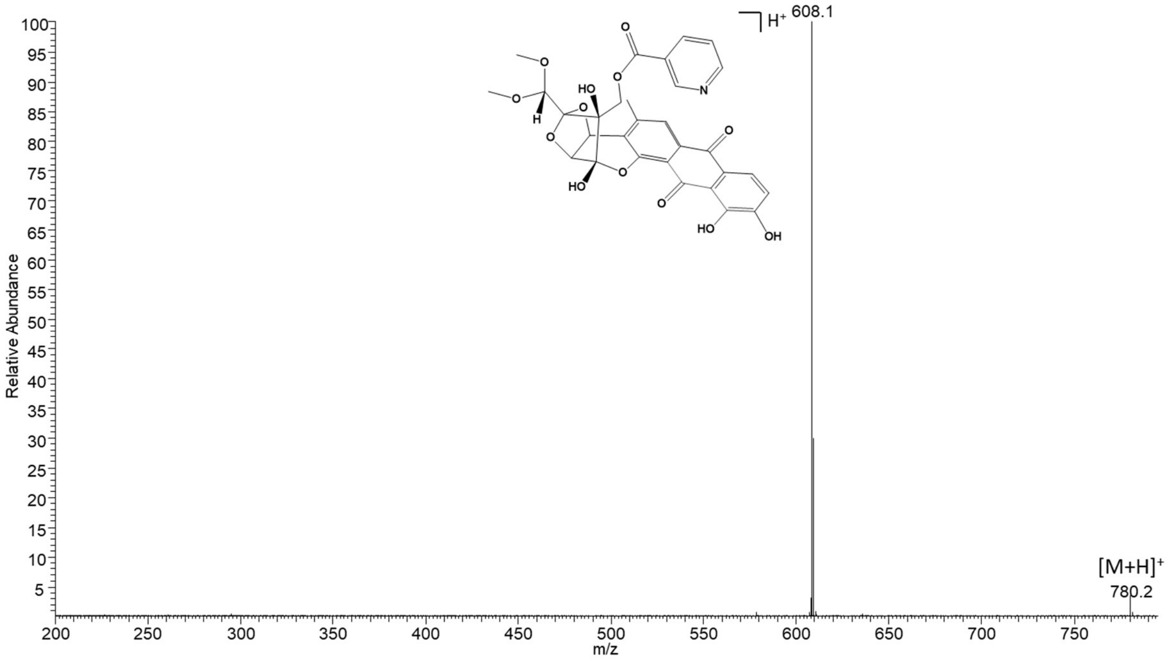

Figure S4. HR-ESI-MS/MS of the quasimolecular ion at *m/z* 780 of trioxacarcin 780 (**1**).

^{1}H NMR spectrum of trioxacarcin 780 (1)

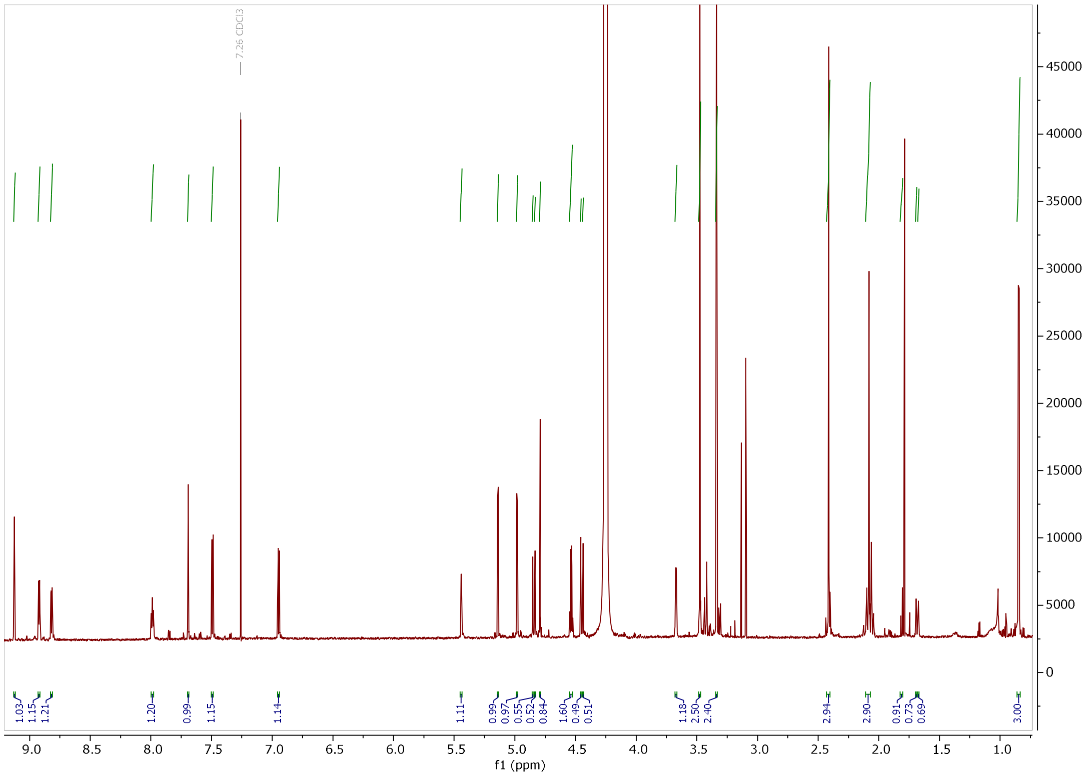

Figure S5. ^{1}H NMR spectrum (800 MHz, $CD_3OD/CDCl_3/DCl$) of trioxacarcin 780 (**1**).

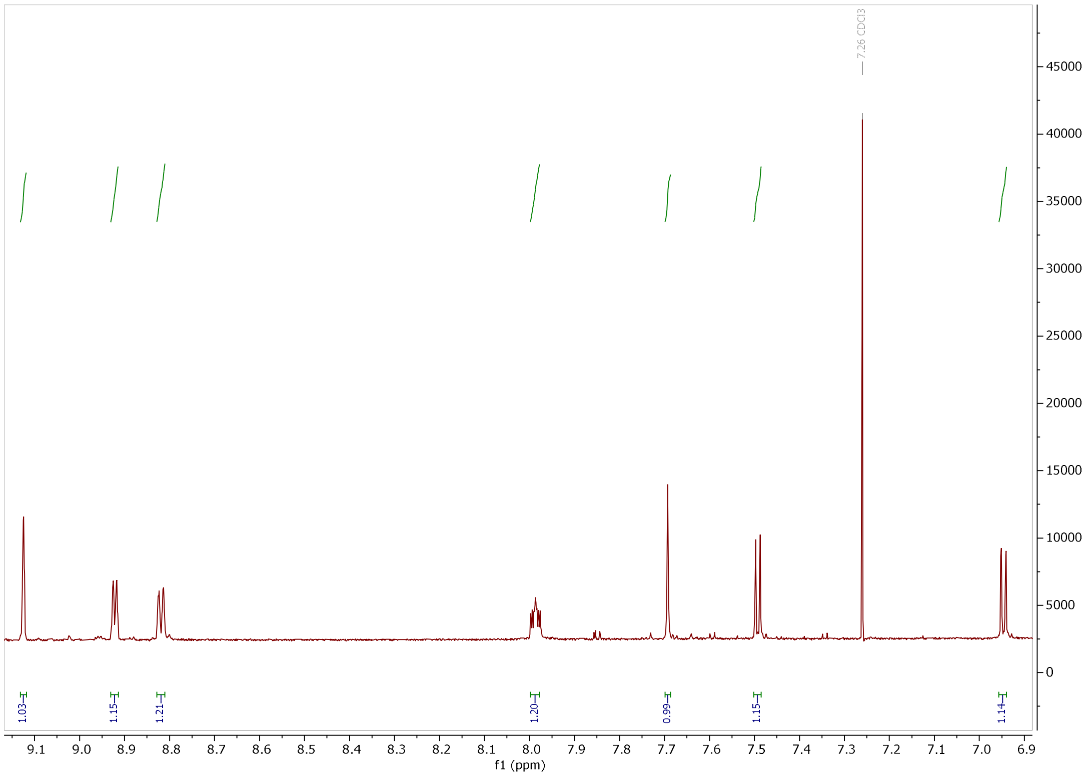

Figure S6. ^{1}H NMR spectrum (800 MHz, $CD_3OD/CDCl_3/DCl$) of trioxacarcin 780 (**1**) aromatic region.

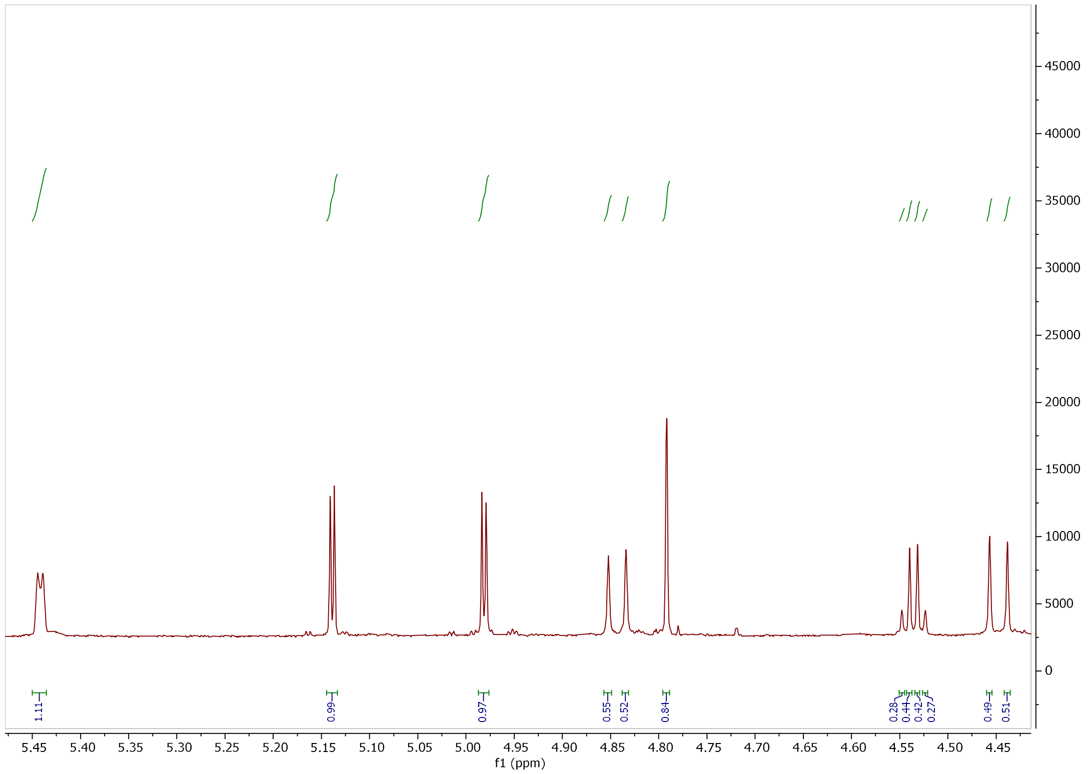

Figure S7. ^{1}H NMR spectrum (800 MHz, $CD_3OD/CDCl_3/DCl$) of trioxacarcin 780 (**1**) 4.4-5.5 region.

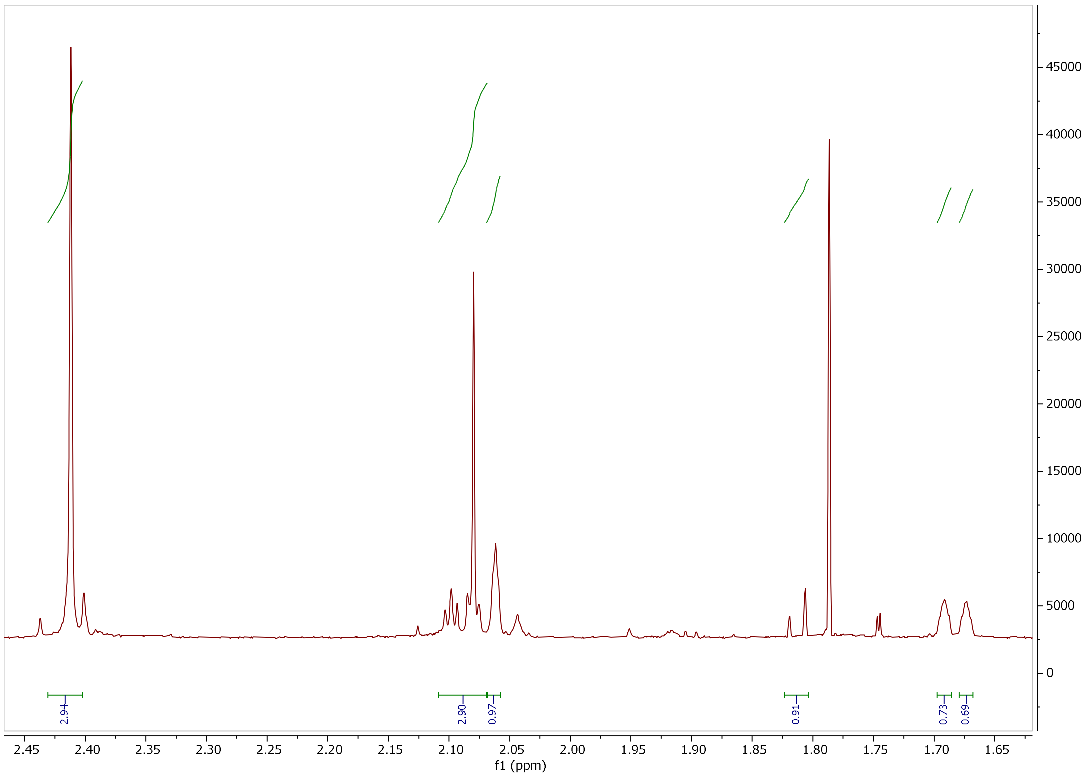

Figure S8. ^{1}H NMR spectrum (800 MHz, $CD_3OD/CDCl_3/DCl$) of trioxacarcin 780 (**1**) 1.6-2.5 region.

^{13}C NMR spectrum of trioxacarcin 780 (1)

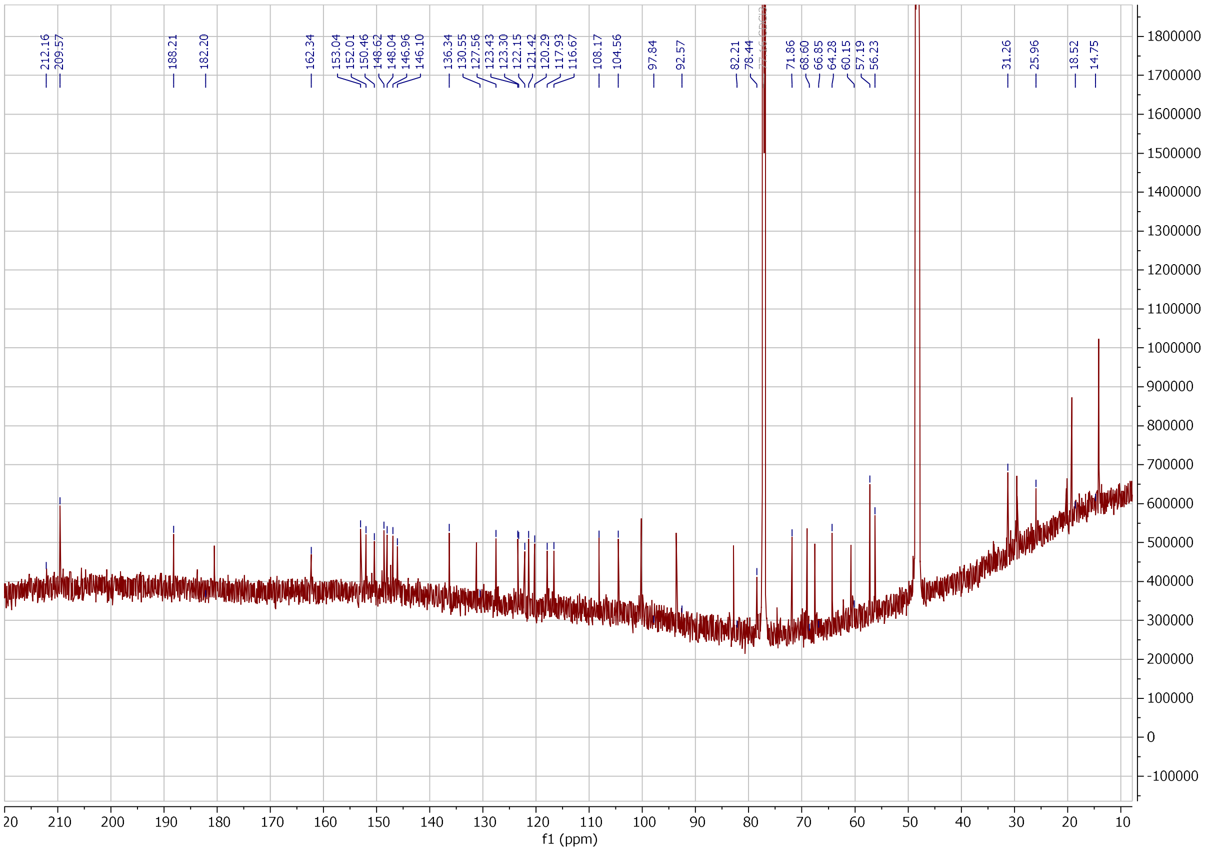

Figure S9. ^{13}C NMR spectrum (201 MHz, $CD_3OD/CDCl_3/DCl$) of trioxacarcin 780 (**1**).

^{1}H-^{1}H-COSY NMR spectrum of trioxacarcin 780 (1)

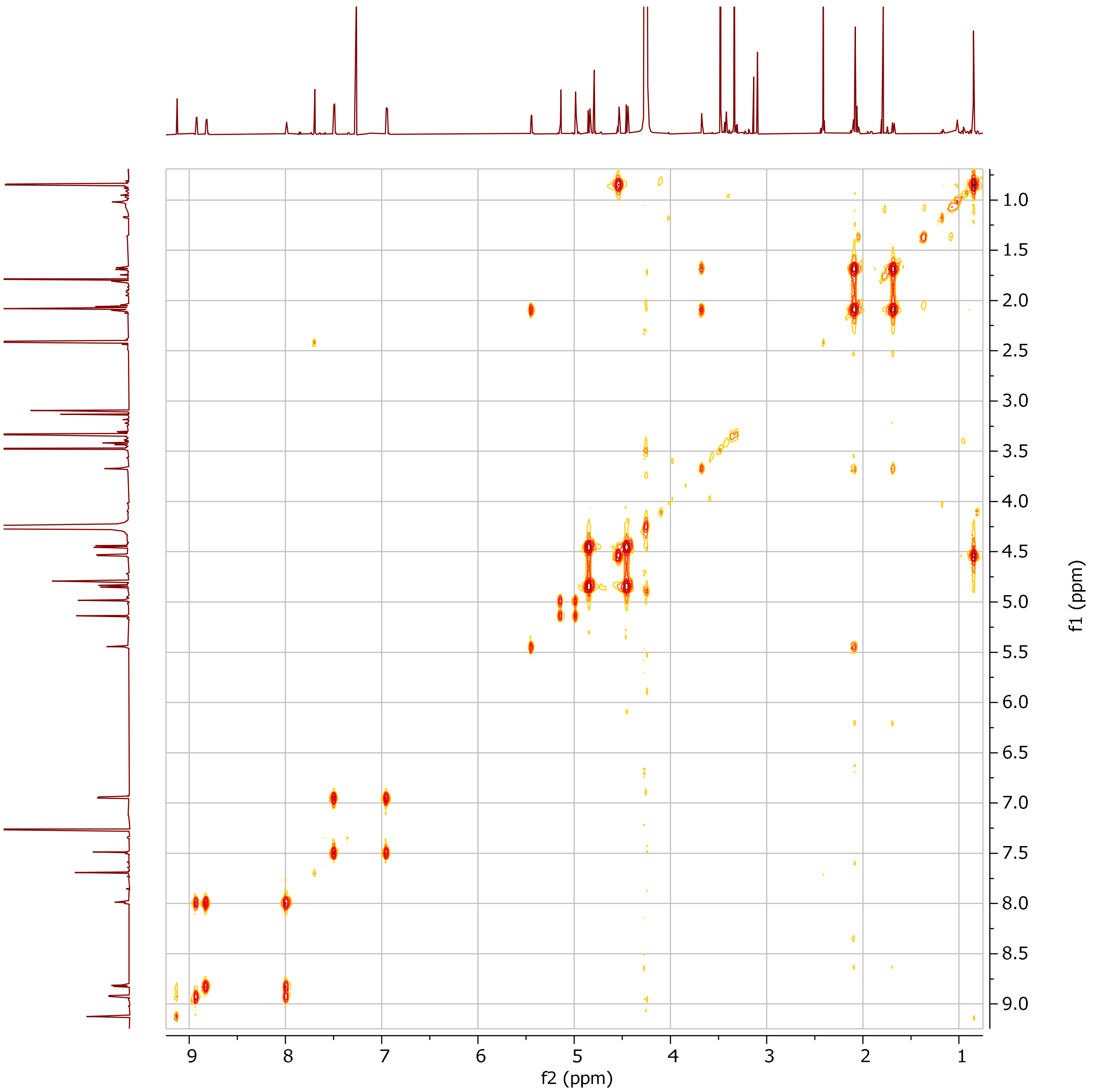

Figure S10. ^{1}H-^{1}H COSY NMR spectrum (800 MHz, $CD_3OD/CDCl_3/DCl$) of trioxacarcin 780 (**1**).

^{1}H-^{13}C-HSQC NMR spectrum of trioxacarcin 780 (1)

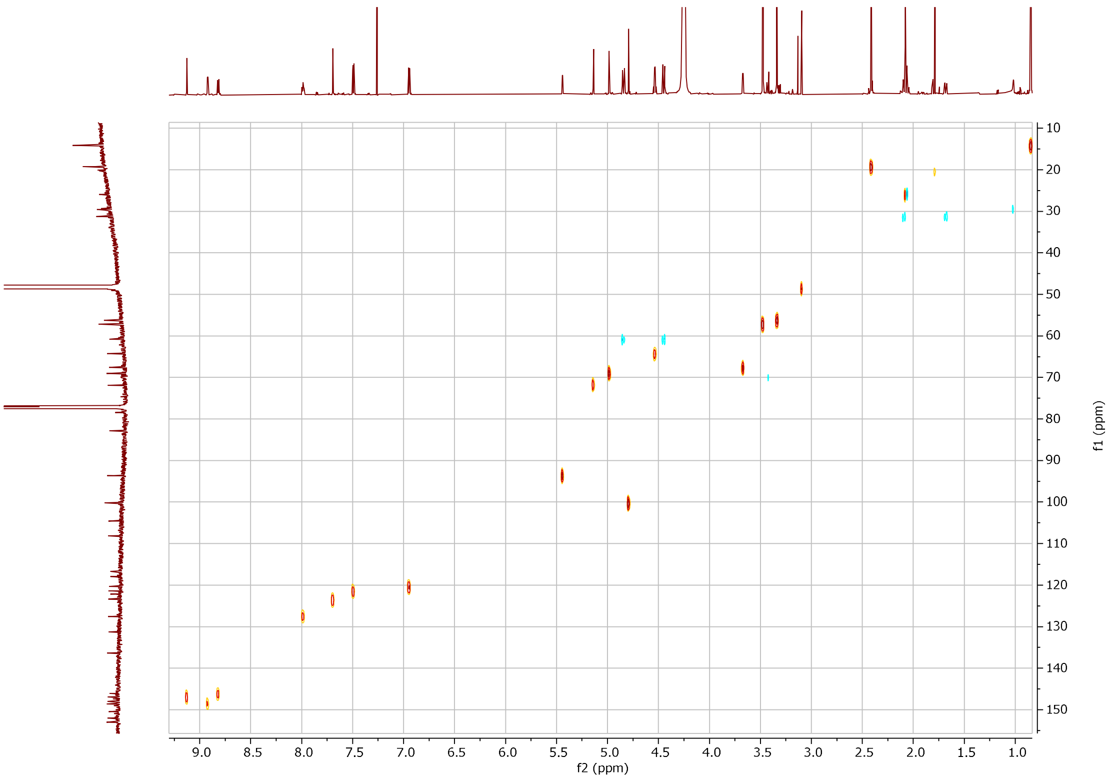

Figure S11. ^{1}H-^{13}C-HSQC NMR spectrum (800 MHz, $CD_3OD/CDCl_3/DCl$) of trioxacarcin 780 (**1**).

^{1}H-^{13}C-HMBC NMR spectrum of trioxacarcin 780 (1)

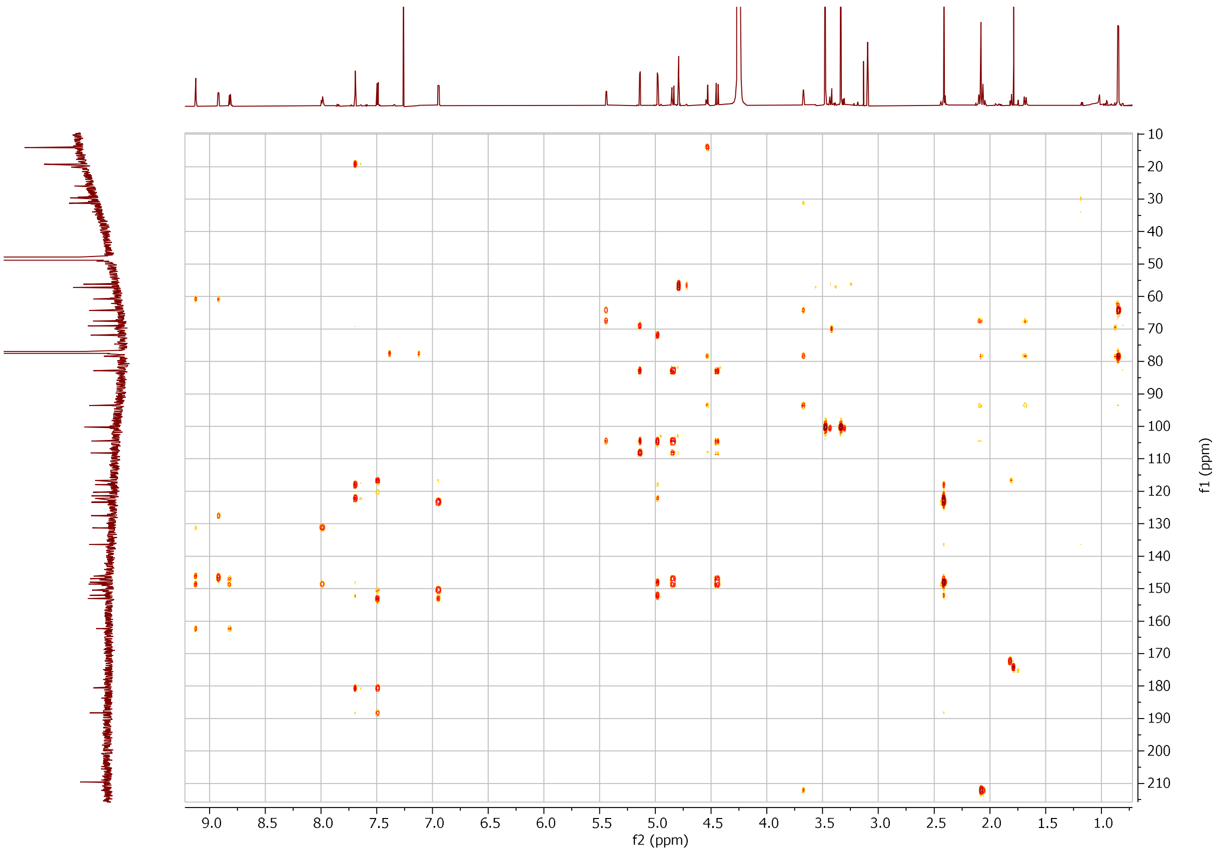

Figure S12. ^{1}H-^{13}C HMBC NMR spectrum (800 MHz, $CD_3OD/CDCl_3/DCl$) of trioxacarcin 780 (**1**).

^{1}H-^{1}H-ROESY NMR spectrum of trioxacarcin 780 (1)

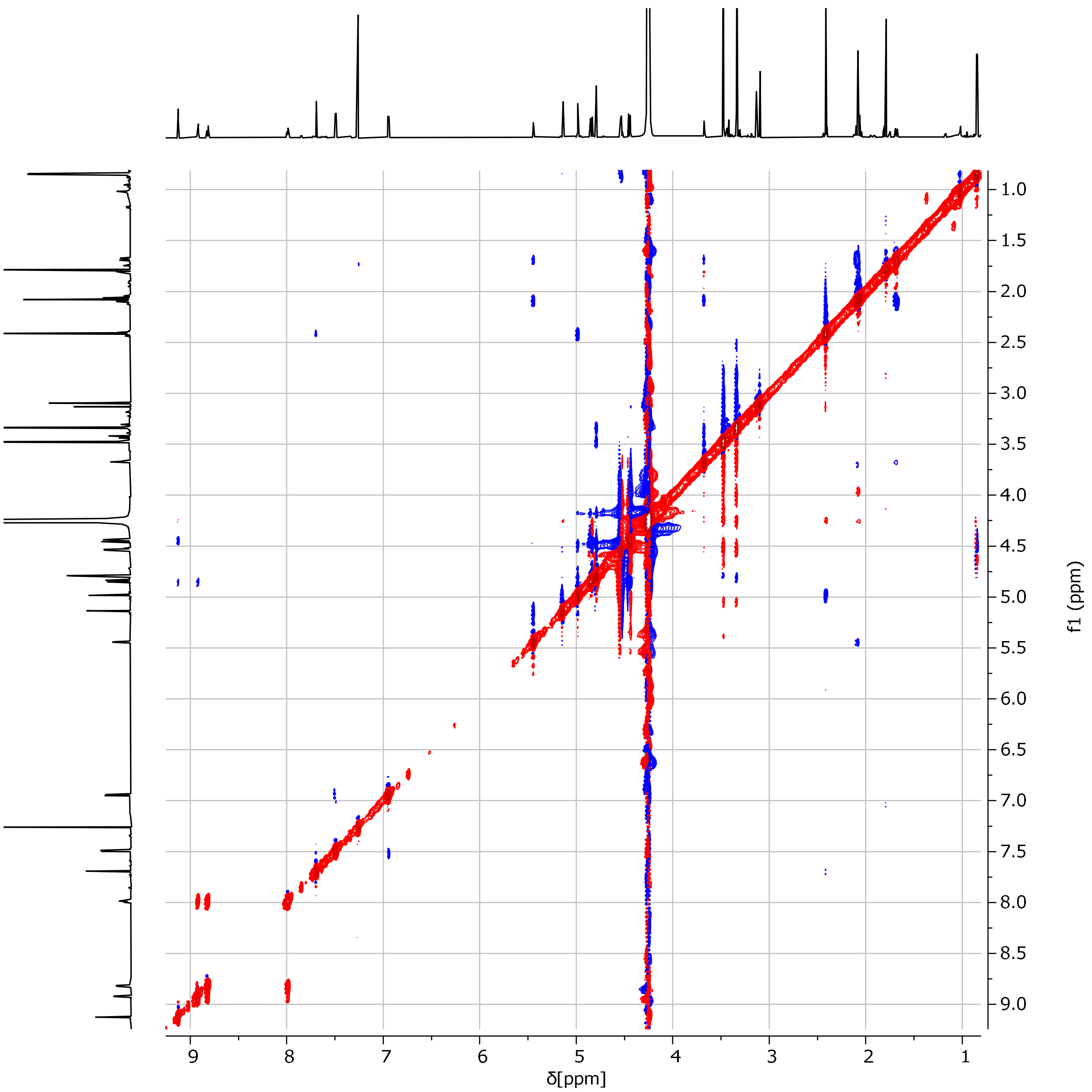

Figure S13. ^{1}H-^{1}H-ROESY NMR spectrum (800 MHz, $CD_3OD/CDCl_3/DCl$) of trioxacarcin 780 (**1**).

Key ^{1}H-^{1}H COSY and ^{1}H-^{13}C HMBC correlations of trioxacarcin 780 (1)

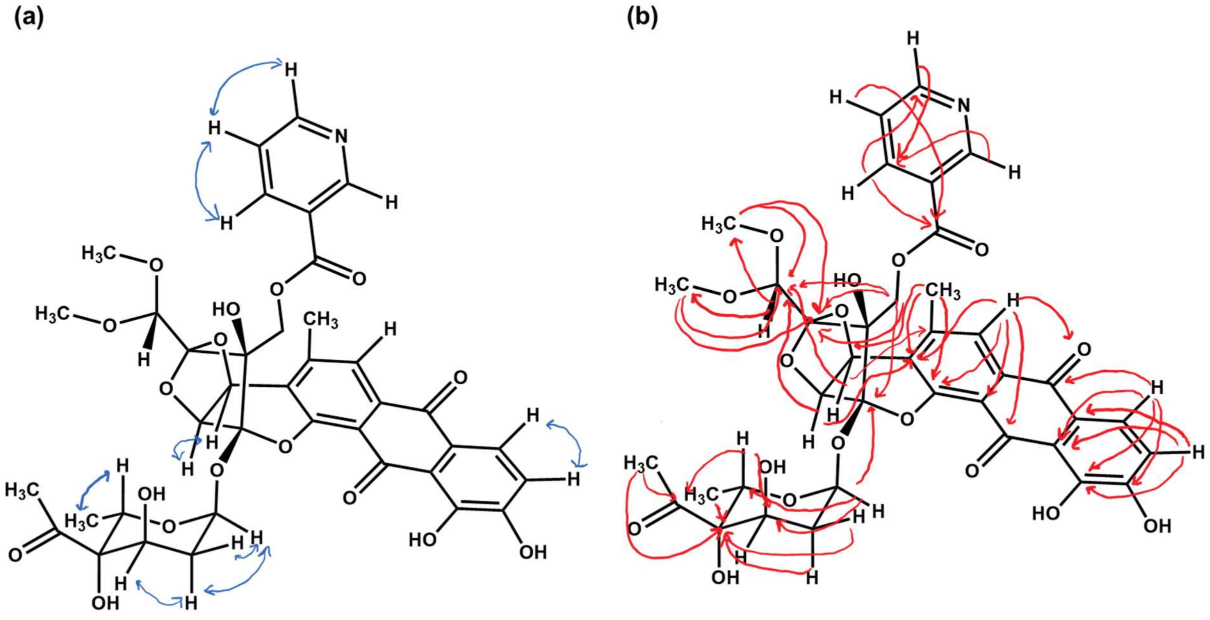

Figure S14. Key (**a**) ^{1}H-^{1}H COSY and (**b**) ^{1}H-^{13}C HMBC correlations of trioxacarcin 780 (**1**).

NMR data of trioxacarcin 780 (1)

3.8 mg from 12 l, reddish-purple amorphous powder.

Table S1. NMR data of trioxacarcin 780 (**1**) (800 MHz, $CD_3OD/CDCl_3/DCl$).

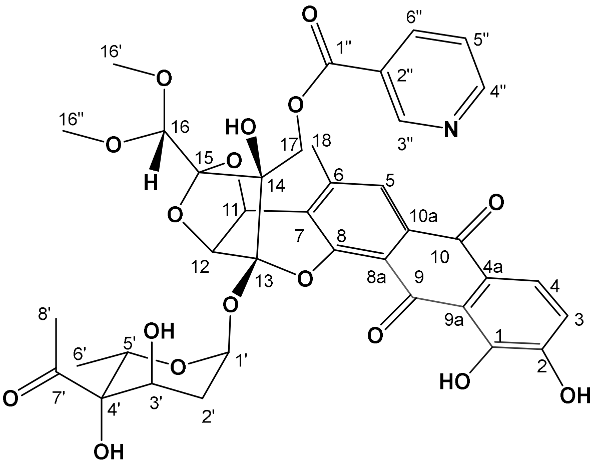

C atom number	δ ¹H/ppm (multiplicity, coupling constant *J*/Hz, integration)	δ ¹³C/ppm
1	-	150.46
2	-	153.04
3	6.94 (d, *J*=8.2, 1H)	120.28
4	7.49 (d, *J*=8.2, 1H)	121.42
4a	-	123.27
5	7.69 (s, 1H)	123.41
6	-	148.04
7	-	122.13
8	-	151.99
8a	-	117.91
9	-	188.21
9a	-	116.67
10	-	180.52
10a	-	117.72
11	4.98 (d, *J*=3.5, 1H)	69.07
12	5.14 (d, *J*=3.5, 1H)	71.87
13	-	104.55
14	-	82.87
15	-	104.56
16	4.79 (s, 1H)	100.24
16′, 16′′	3.34 (s, 3H), 3.48 (s, 3H)	56.27, 57.20
17	4.44 (d, *J*=14.7, 1H), 4.83 (d, *J*=14.7, 1H)	60.82
18	2.41 (s, 3H)	19.21
1′	5.44 (d, *J*=4.3, 1H)	93.66
2′	1.67-1.7 (m, 1H), 2.09 (dt, *J*=14.3, *J*=4.0, 1H)	31.27
3′	3.66-3.68 (m, 1H)	67.56
4′	-	78.53
5′	4.54 (q, *J*=6.5, 1H)	64.31
6′	0.84 (d, *J*=6.5, 3H)	14.12
7′	-	212.13
8′	2.08, 2.06 enol(s, 3H)	25.93
1′′	-	162.29
2′′	-	131.29
3′′	9.12 (s, 1H)	146.97
4′′	8.92 (dd, *J*=8.2, *J*=1.0, 1H)	148.63
5′′	7.97-8.00 (m, 1H)	127.56
6′′	8.82 (dd, *J*=8.2, *J*=1.3, 1H)	146.11

Trioxacarcin 608 aglycon (8)

UV-Vis spectrum of trioxacarcin 608 (8)

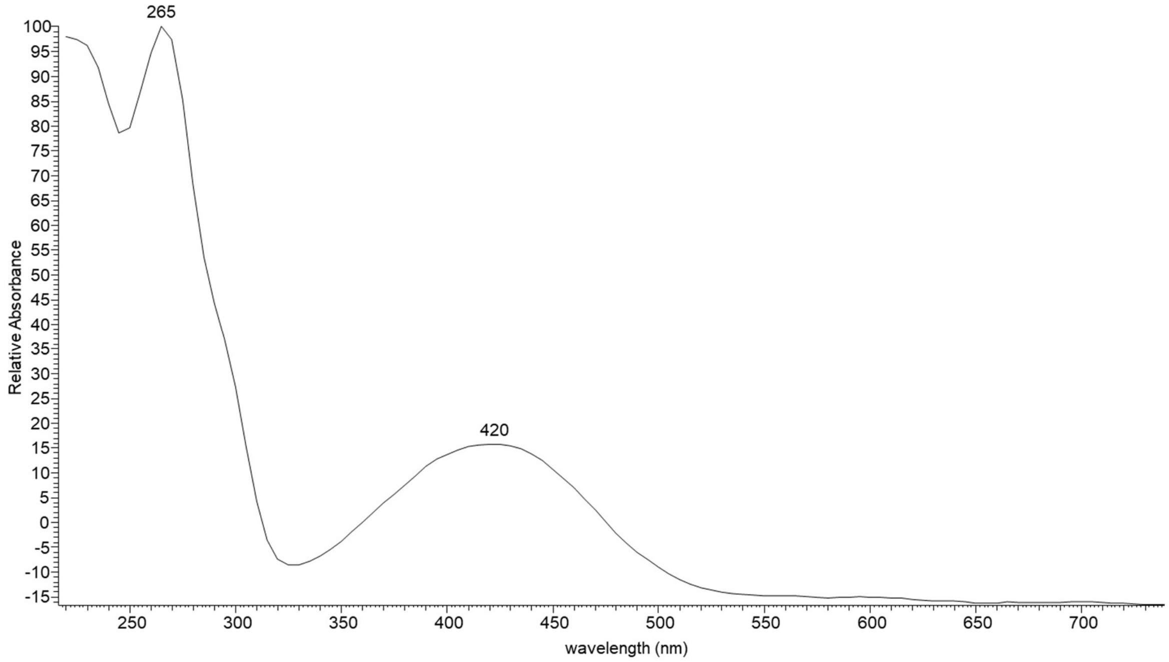

Figure S15. UV-Vis spectrum of trioxacarcin 608 (**8**).

HR-ESI-MS of trioxacarcin 608 (8)

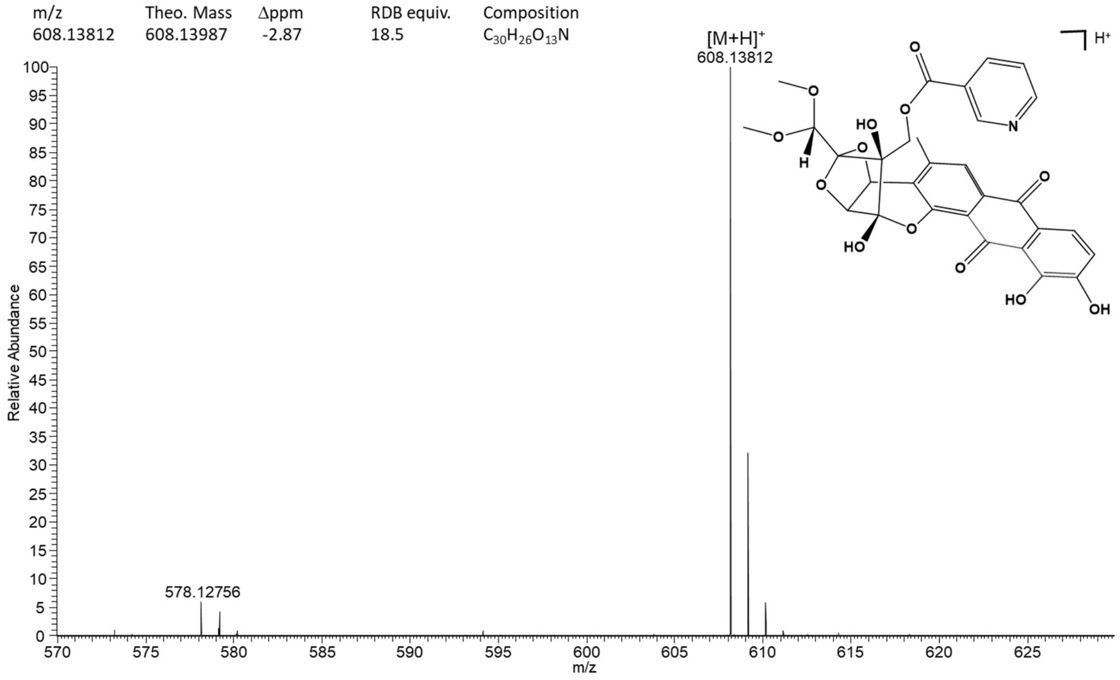

Figure S16. HR-ESI-MS of trioxacarcin 608 (**8**). Retention time 18.7 min, Macherey-Nagel RP8 column (250 × 2 mm, 5 µm), LC-MS program 1.

HR-ESI-MS/MS of trioxacarcin 608 (8)

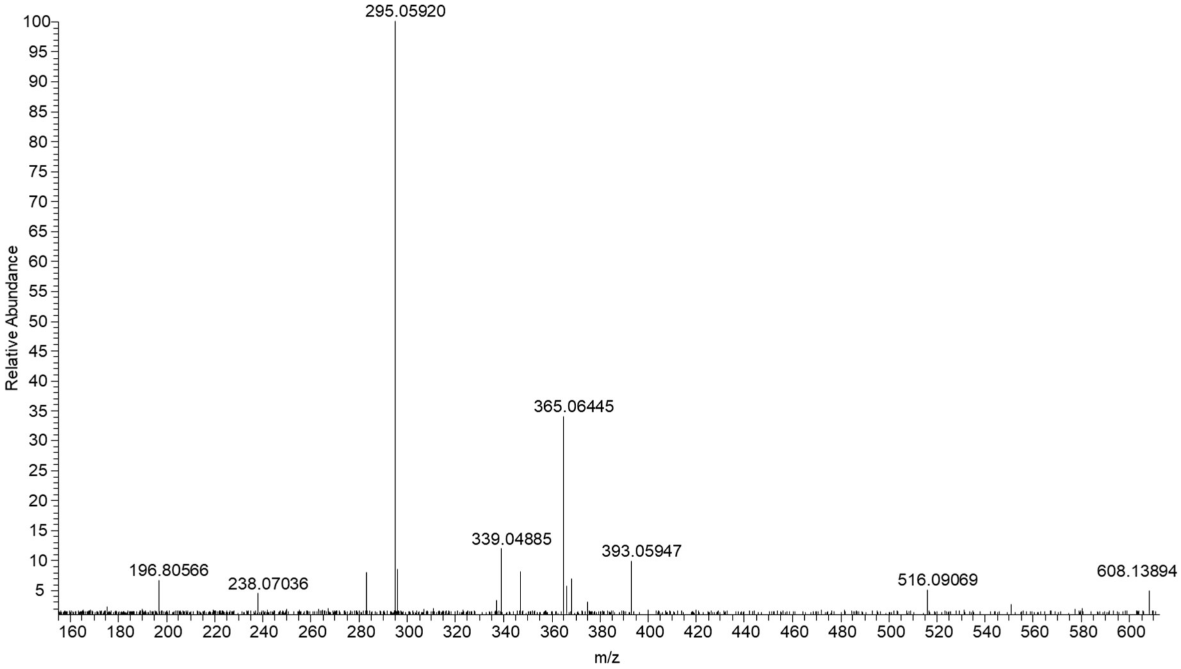

Figure S17. MS/MS fragmentation of trioxacarcin 608 (**8**). MS/MS of the quasimolecular ion *m/z* 608 of **8**.

Hydrolysis of trioxacarcin 780 (1)

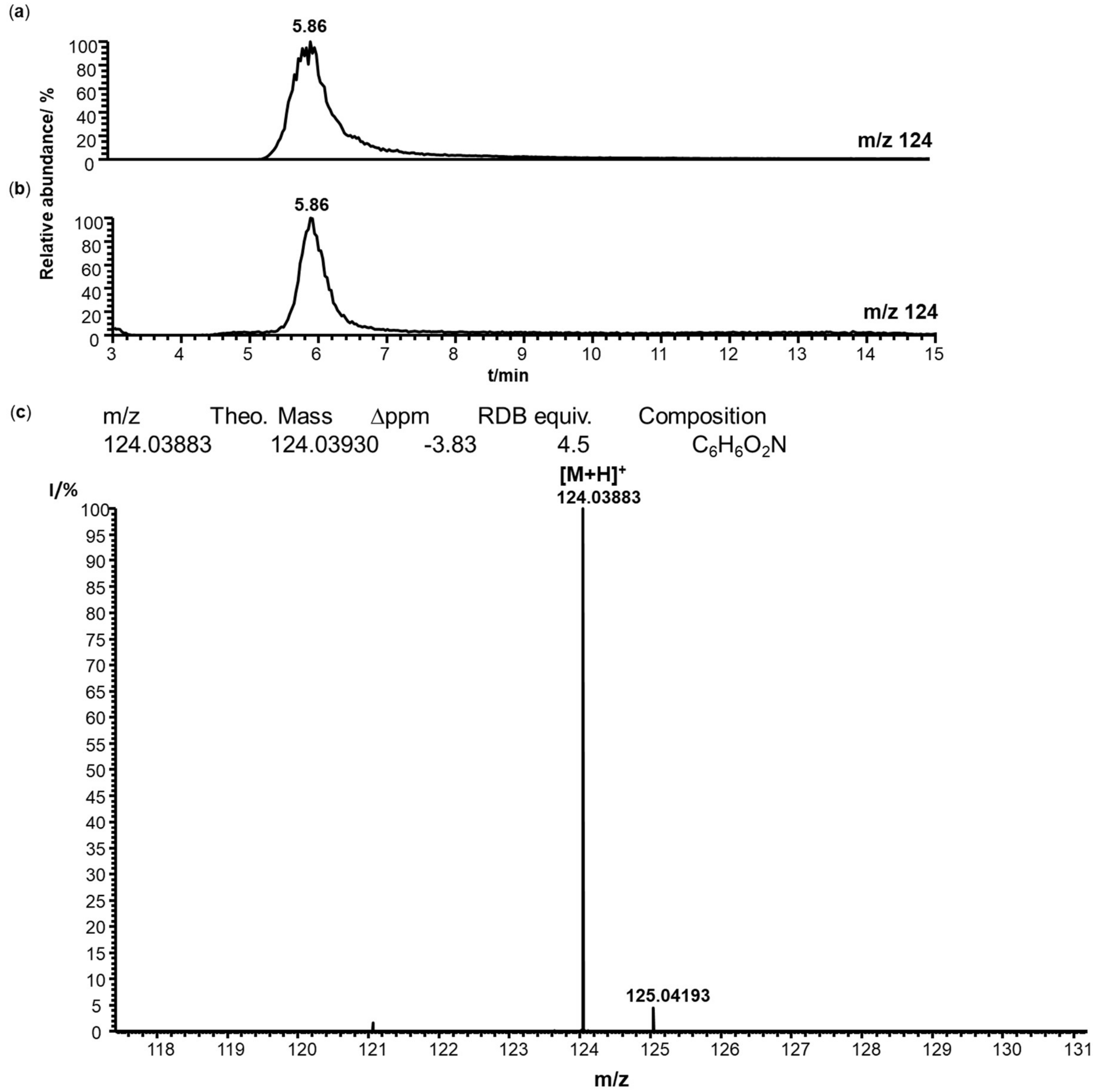

Figure S18. Detection of nicotinic acid (*m*/*z* 124) after the hydrolysis of trioxacarcin 780 (**1**). The retention time of *m*/*z* 124 is 5.86 min. (**a**) Standard of nicotinic acid. (**b**) Released nicotinic acid from hydrolyzed trioxacarcin 780 (**1**), (**c**) HR-ESI-MS of nicotinic acid ($[M+H]^+$ 124.03883, Δppm -3.83, $C_6H_6O_2N$), hydrolyzed from trioxacarcin 780 (**1**). The retention time of the hydrolyzed nicotinic acid matches the retention time of nicotinic acid standard. Phenomenex Synergi Polar RP column (250 × 2 mm, 4 μm), LC-MS program 2.

Gutingimycin A (7)

UV-Vis spectrum of gutingimycin A (7)

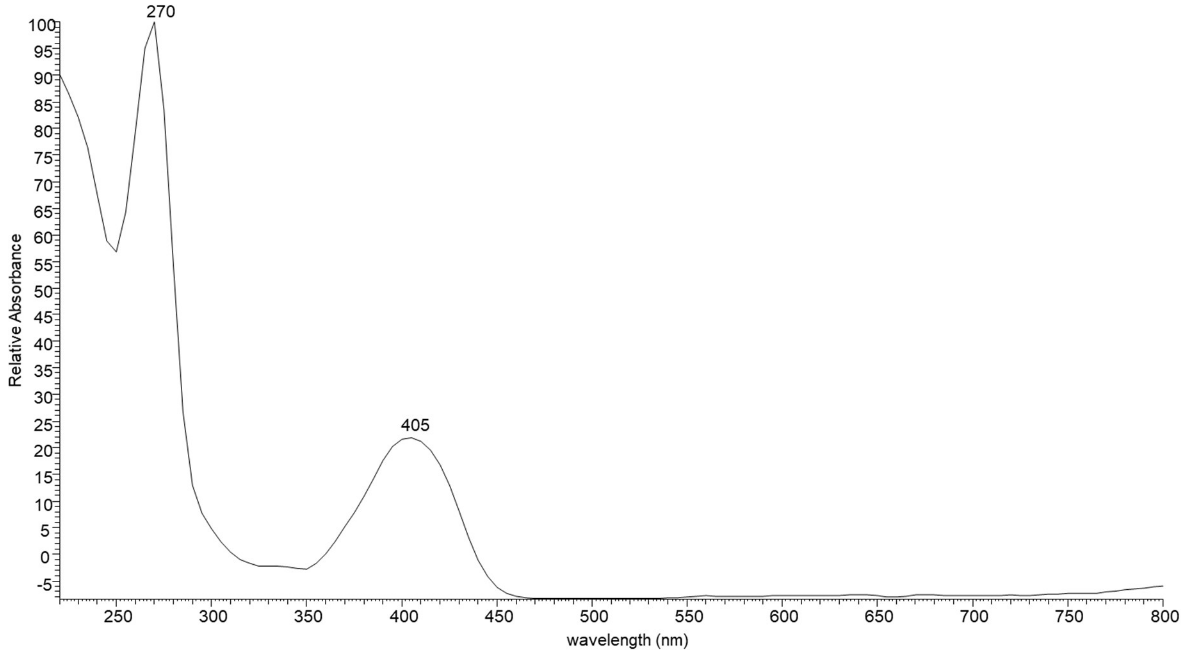

Figure S19. UV-Vis spectrum of gutingimycin A (**7**)

HR-ESI-MS of gutingimycin A (7)

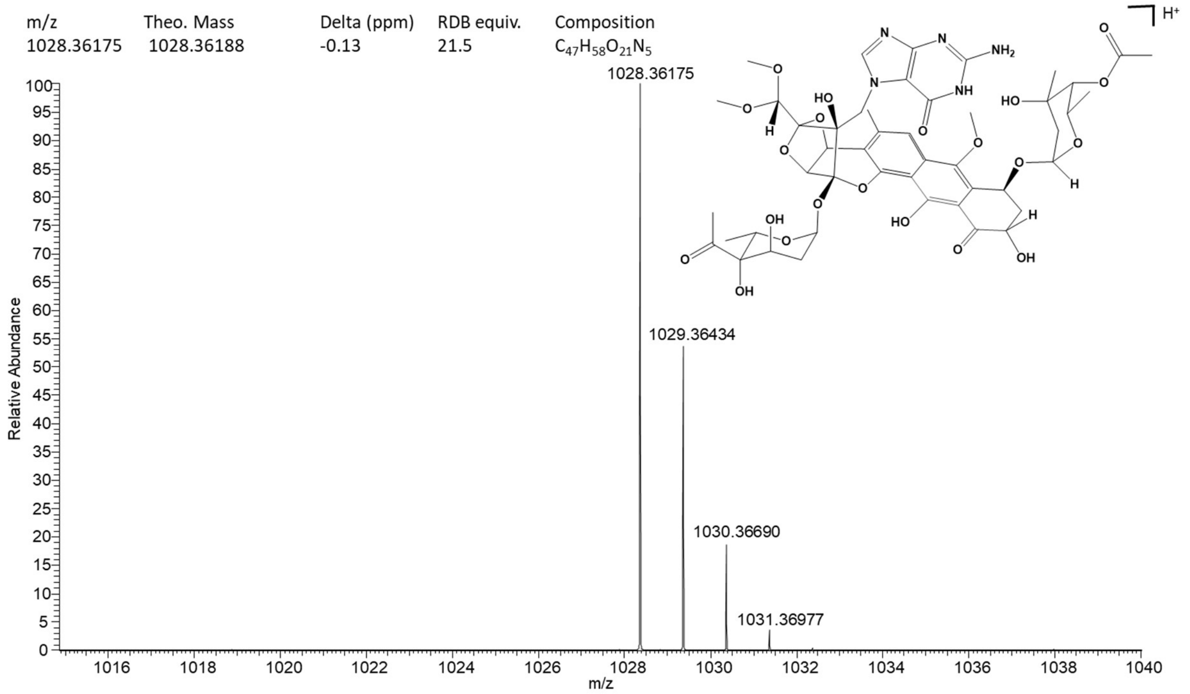

Figure S20. HR-ESI-MS of gutingimycin A (**7**). Retention time 24.16 min, Macherey-Nagel RP8 column (250 × 2 mm, 5 µm), LC-MS program 1.

ESI-MS/MS of gutingimycin A (7)

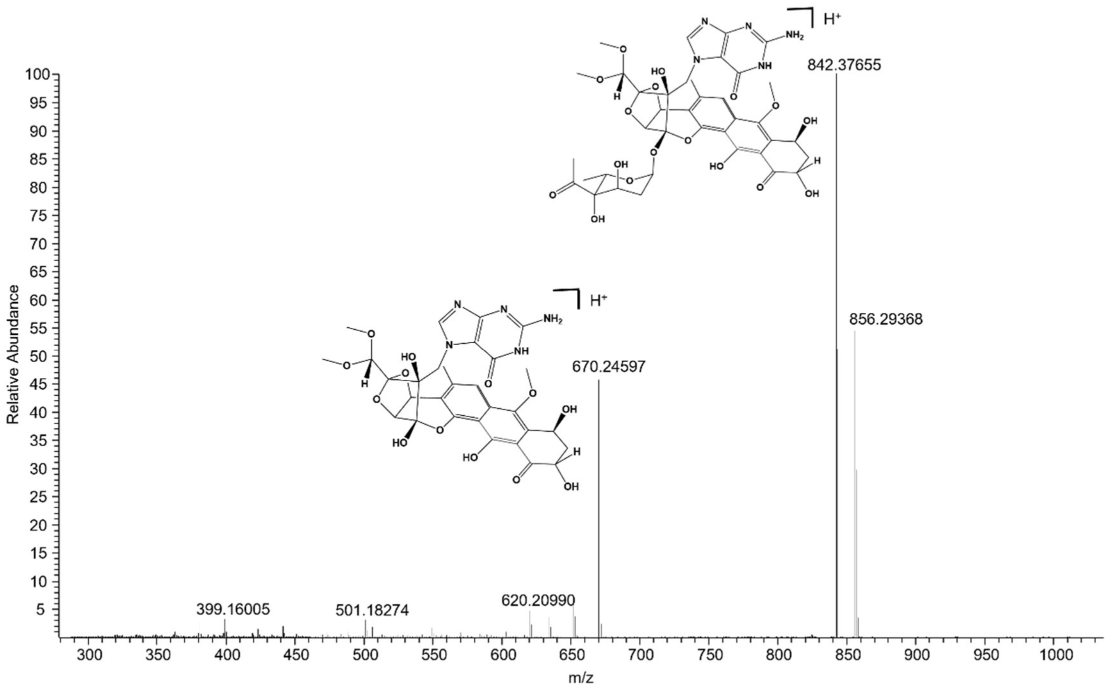

Figure S21. HR-ESI-MS/MS fragmentation of gutingimycin A (**7**). MS/MS of the quasimolecular ion *m/z* 1028 of **7**.

^{1}H NMR spectrum of gutingimycin A (7)

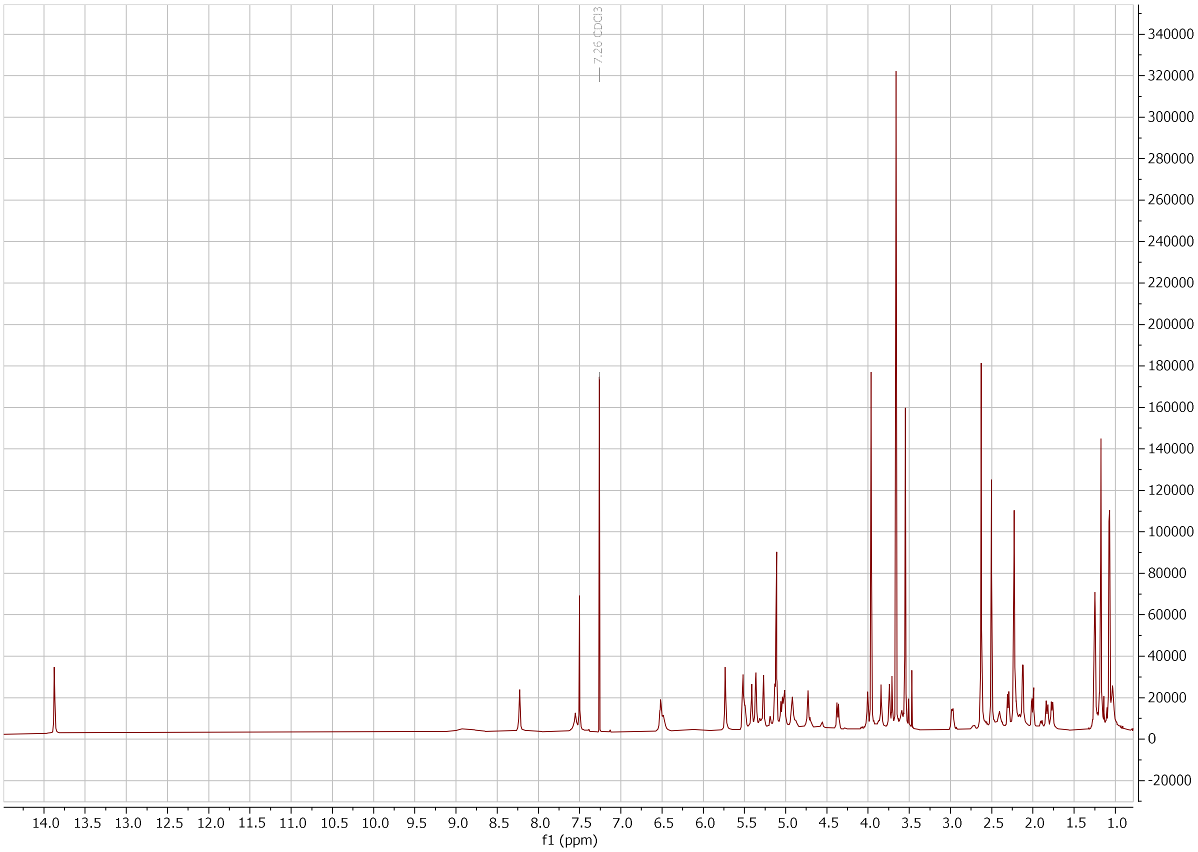

Figure S22. ^{1}H NMR spectrum of gutingimycin A (**7**) (800 MHz, $CDCl_3$).

^{13}C NMR spectrum of gutingimycin A (7)

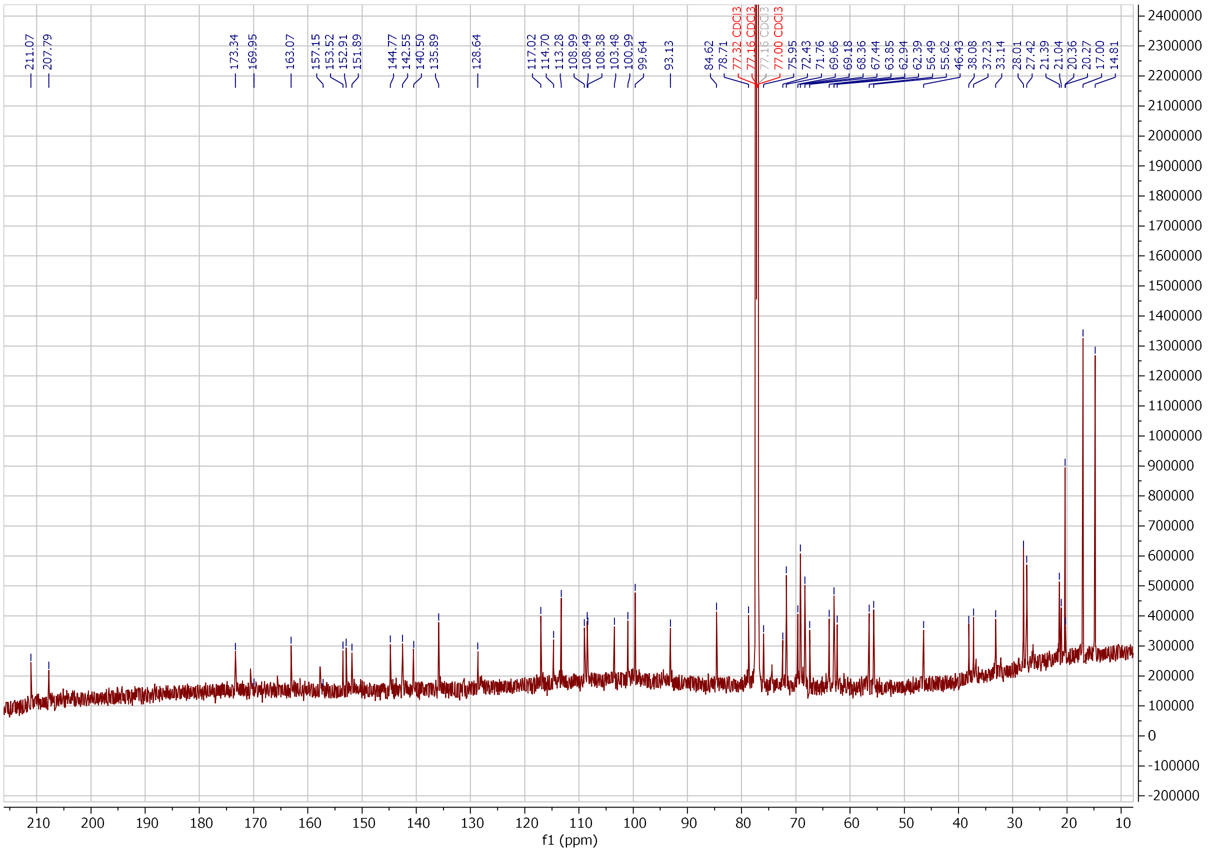

Figure S23. ^{13}C NMR spectrum of gutingimycin A (**7**) (201 MHz, $CDCl_3$).

^{1}H-^{1}H-COSY NMR spectrum of gutingimycin A (7)

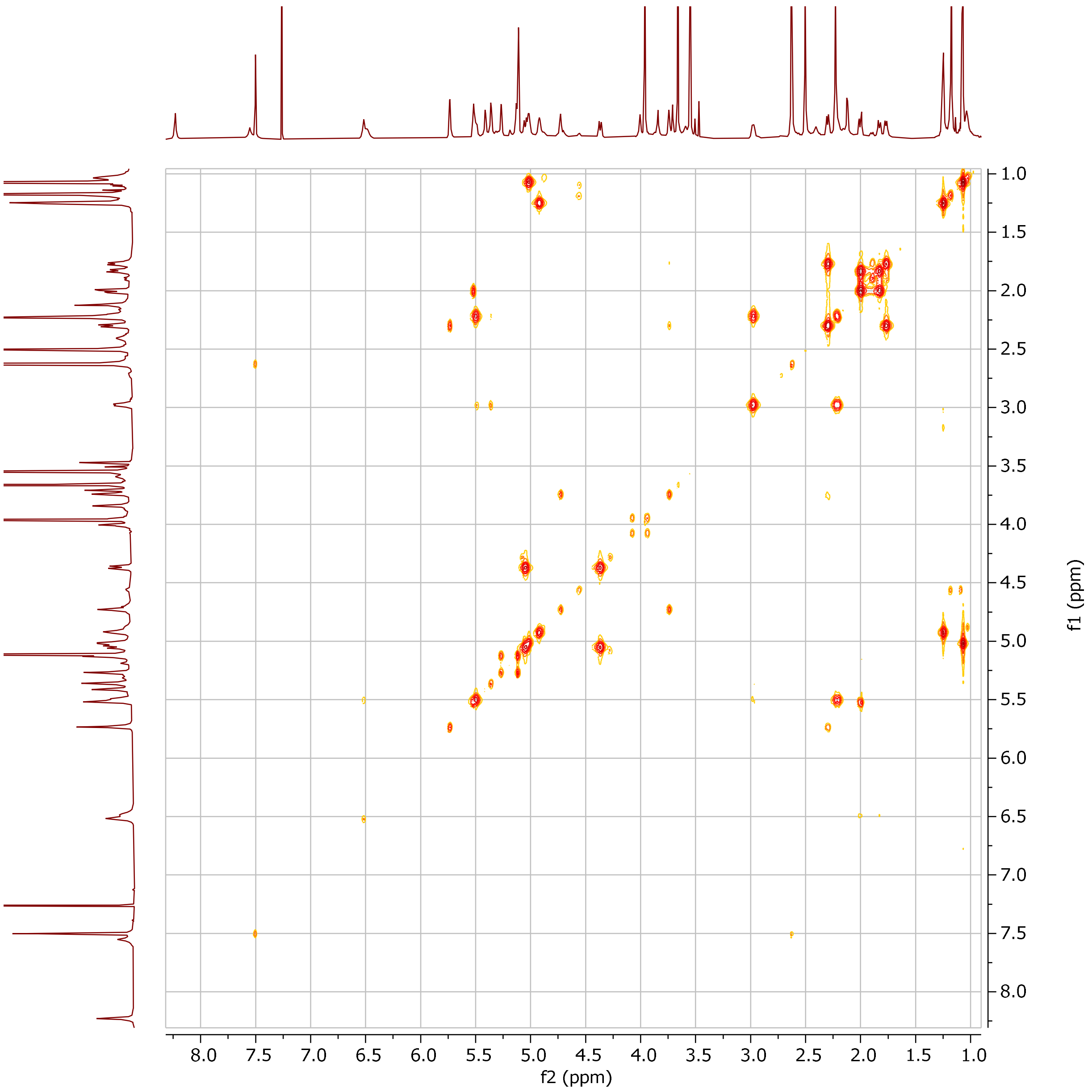

Figure S24. ^{1}H-^{1}H-COSY NMR spectrum of gutingimycin A (**7**) (800 MHz, $CDCl_3$).

¹H-¹³C-HSQC NMR spectrum of gutingimycin A (7)

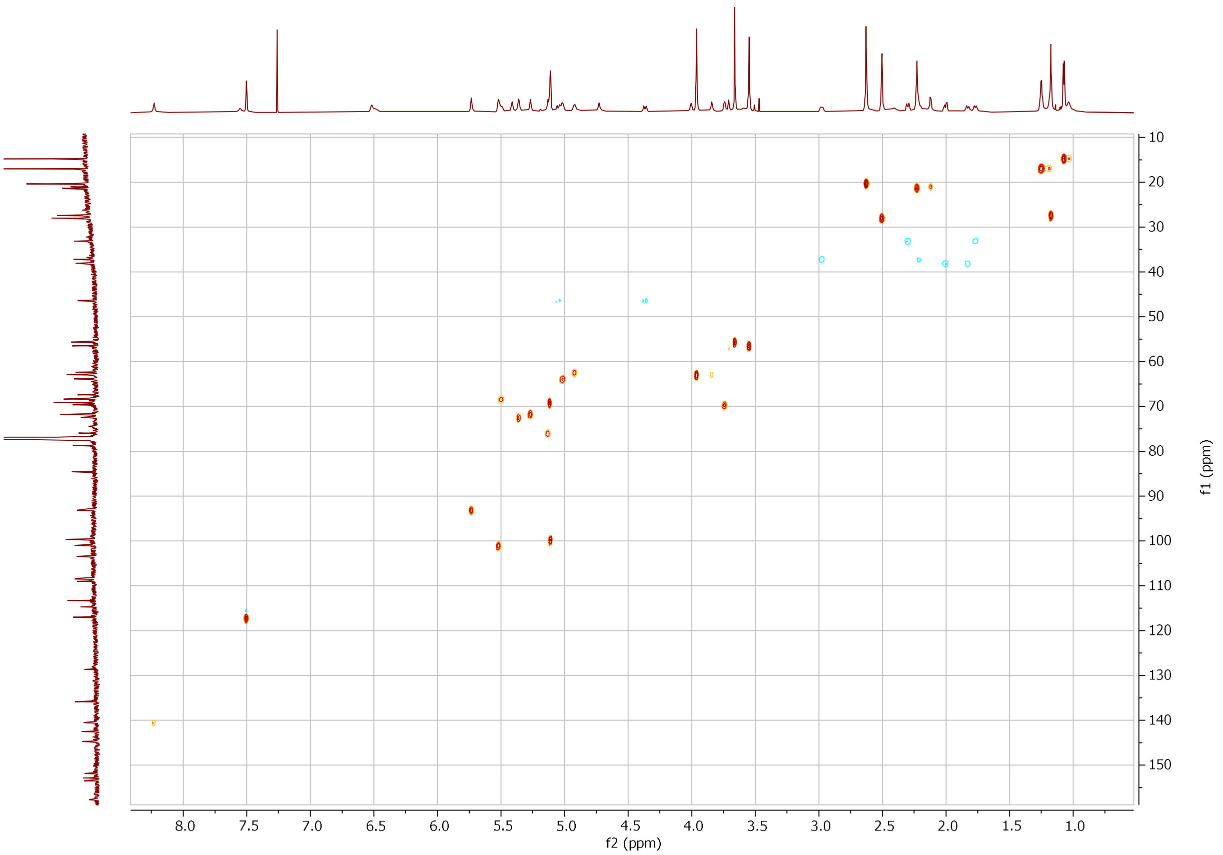

Figure S25. ¹H-¹³C-HSQC NMR spectrum of gutingimycin A (**7**) (800 MHz, $CDCl_3$).

^{1}H-^{13}C-HMBC NMR spectrum of gutingimycin A (7)

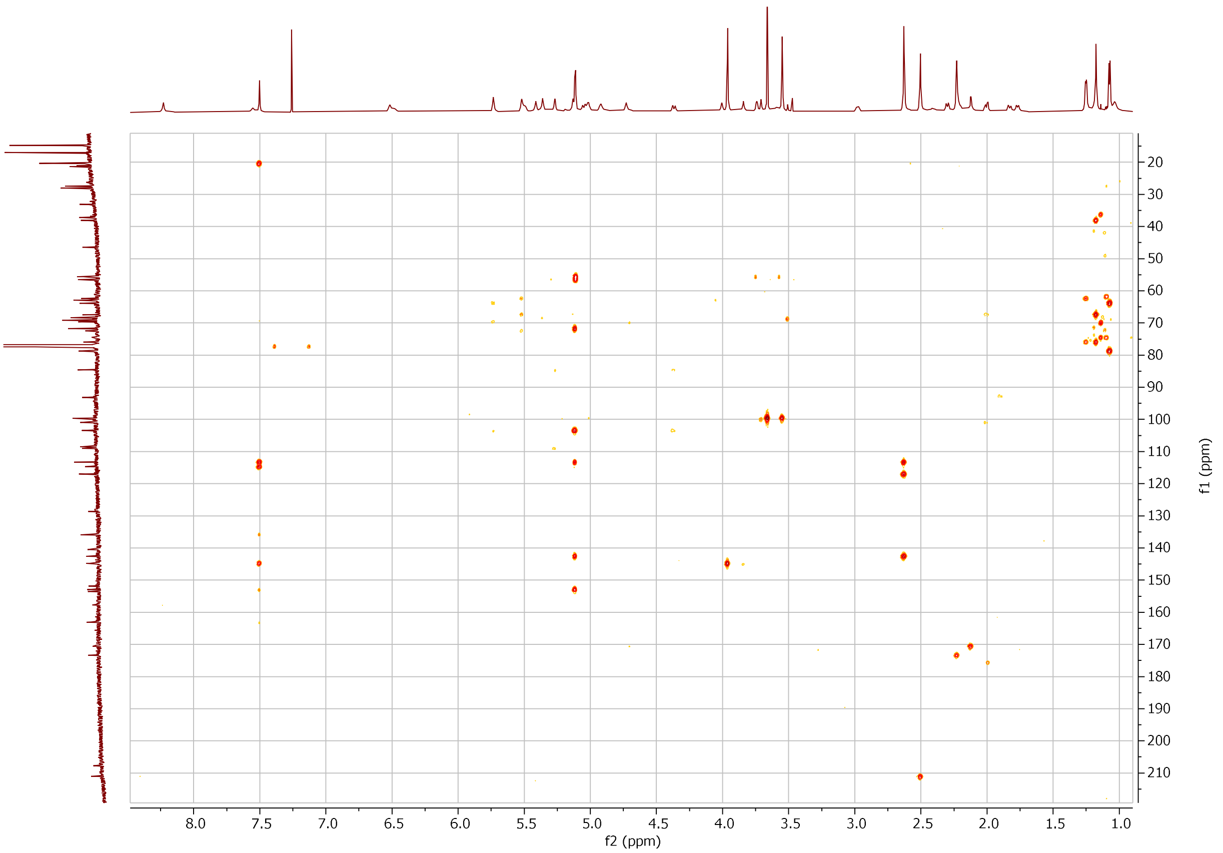

Figure S26. ^{1}H-^{13}C-HMBC NMR spectrum of gutingimycin A (**7**) (800 MHz, $CDCl_3$).

^{1}H-^{1}H-ROESY spectrum of gutingimycin A (7)

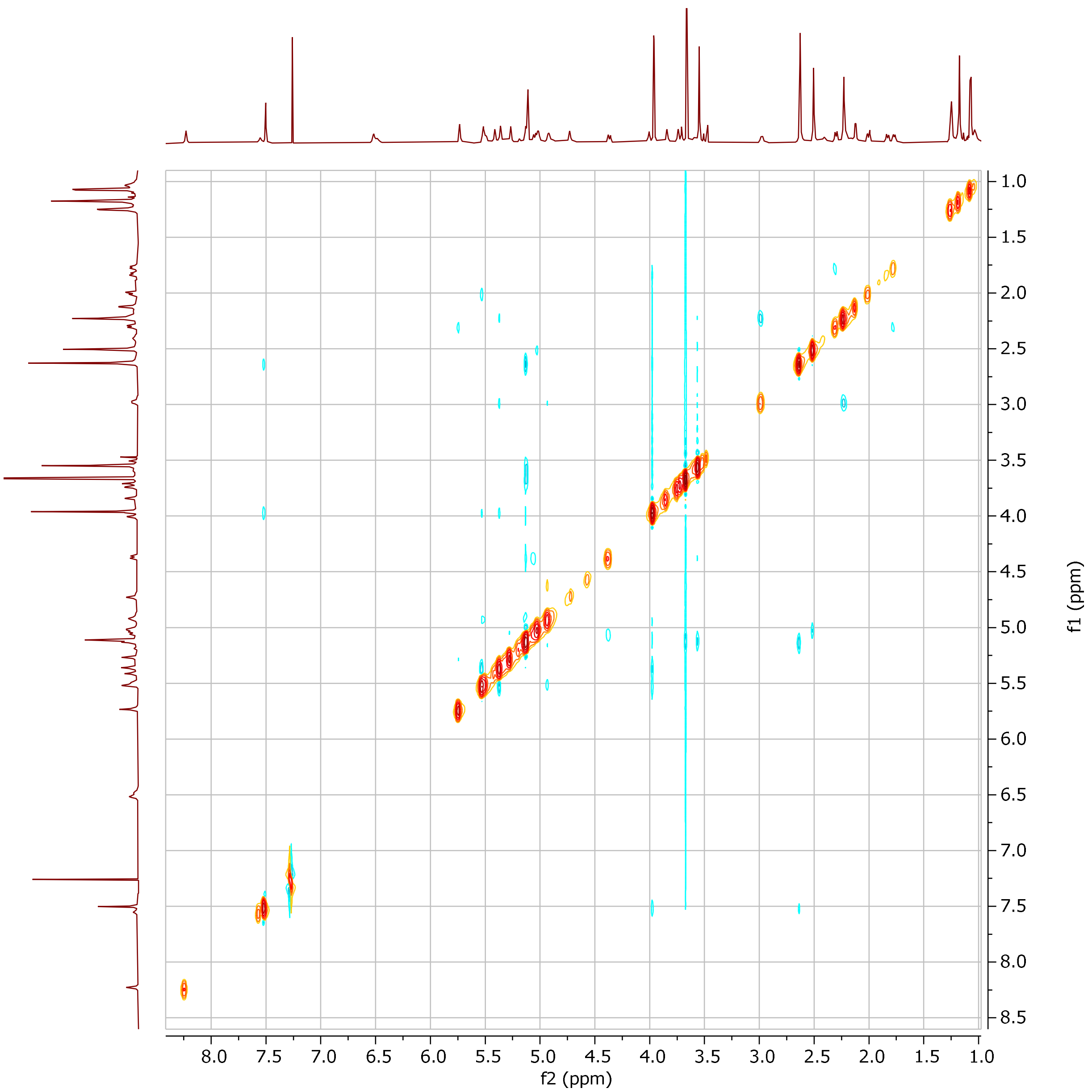

Figure S27. ^{1}H-^{1}H-ROESY NMR spectrum of gutingimycin A (7) (800 MHz, $CDCl_3$).

NMR data of gutingimycin A (7)

Table S2. NMR data of gutingimycin A (**7**) (800 MHz, $CDCl_3$).

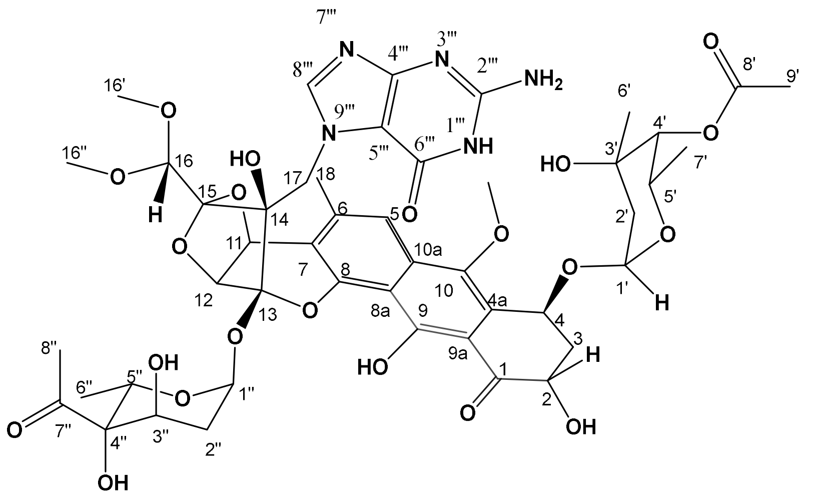

C atom number	δ ¹H/ppm (multiplicity, coupling constant *J*/Hz, integration)	δ ¹³C/ppm Measured literature value in brackets
1	-	207.79 (207.6)
2	5.48-5.51 (m,1H)	68.36 (68.2)
3	2.19-2.21 (m, 1H), 2.94-3.04 (m, 1H)	37.23 (37.0)
4	5.35-5.37 (m, 1H)	72.43 (72.2)
4a	-	128.64 (128.4)
5	7.50 (s, 1H))	117.02 (116.8)
6	-	144.55 (142.4)
7	-	113.28 (113.1)
8	-	152.91 (152.7)
8a	-	114.70 (114.5)
9	-	163.07 (162.9)
9a	-	108.99 (108.8)
10	-	144.77 (144.6)
10a	-	135.89 (135.7)
11	5.13 (1H)	69.18 (69.0)
12	5.27 (1H)	71.76 (71.6)
13	-	103.48 (103.3)
14	-	84.62 (84.4)
15	-	108.38 (108.2)
16	5.12 (s, 1H)	99.64 (99.5)
16′, 16′′	3.66 (s,3H)<br>3.55 (s, 3H)	55.62 (55.4)<br>56.49 (56.2)
17	4.37 (d, *J*=15.8, 1H)<br>5.05 (d, *J*=15.7,1H)	46.43 (46.2)
18	2.63 (s, 3H)	20.36 (20.2)
1′	5.50-5.53 (m, 1H)	100.99 (100.8)
2′	1.85 (d, J=14.9, 1H) 2.23 (dd, *J*=14.9, *J*=5.4, 1H)	38.08 (37.8)
3′	-	67.44 (67.2)
4′	5.12-5.14 (m, 1H)	75.95 (75.8)
5′	4.90-4.94 (m,1H)	62.39 (62.2)
6′	1.18 (s,3H)	27.42 (27.2)
7′	1.25 (d, *J*=6.5, 3H)	17.00 (16.8)
8′	-	173.34 (173.2)
9′	2.23 (s, 3H)	21.39 (21.2)
1′′	5.72-5.75 (m, 1H)	93.13 (92.9)
2′′	1.77 (d, *J*=13.6, 1H)<br>2.28-2.32 (m, 1H)	33.14 (32.9)

C atom number	δ ^{1}H/ppm (multiplicity, coupling constant *J*/Hz, integration)	δ ^{13}C/ppm Measured literature value in brackets
3′′	3.72-3.77 (m, 1 H)	69.66 (69.5)
4′′	-	78.71 (78.5)
5′′	4.99-5.03 (m, 1H)	63.85 (63.7)
6′′	1.07 (d, *J*=6.5, 3H)	14.81(14.6)
7′′	-	211.07 (210.9)
8′′	2.50 (s, 3H)	28.01 (27.8)
2′′′	-	151.89 (151.7)
4′′′	-	153.52 (153.0)
5′′′	-	108.49 (108.3)
6′′′	-	157.15 (157.7)
8′′′	8.23 (s, 1H)	140.50 (140.4)

Trioxacarcin 692 (2)

UV-Vis spectrum of trioxacarcin 692 (2)

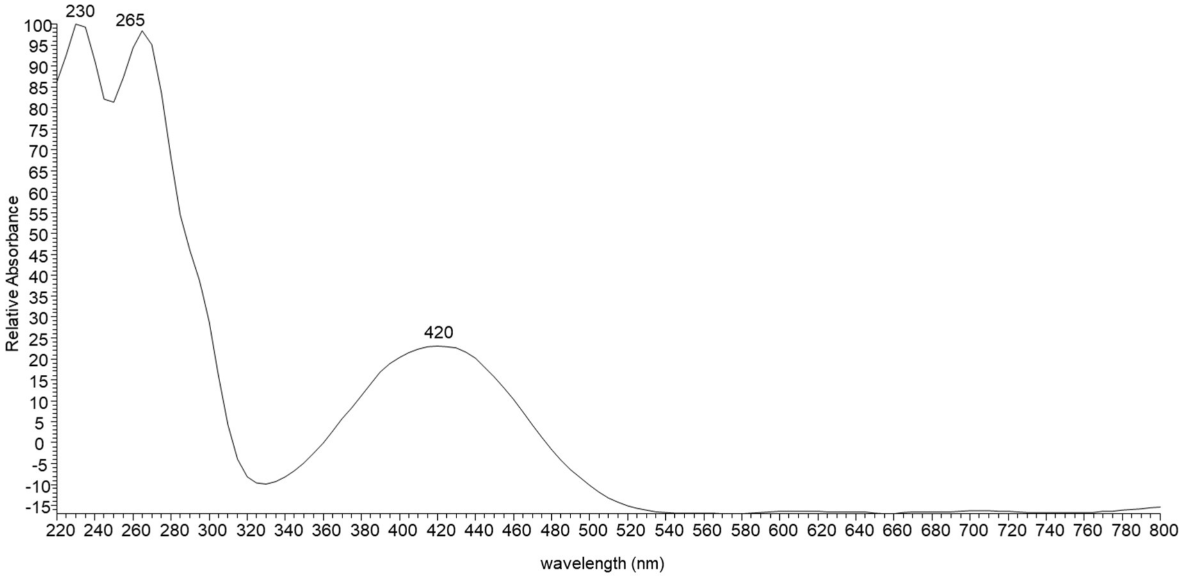

Figure S28. UV-Vis spectrum of trioxacarcin 692 (**2**).

HR-ESI-MS of trioxacarcin 692 (2)

m/z	Theo. Mass	Δppm	RDB equiv.	Composition
697.17407	697.17446	0.164	15.5	$C_{32}H_{34}O_{16}Na$
692.21887	692.21906	0.360	14.5	$C_{32}H_{38}O_{16}N$
713.14813	713.14839	0.402	15.5	$C_{32}H_{34}O_{16}K$

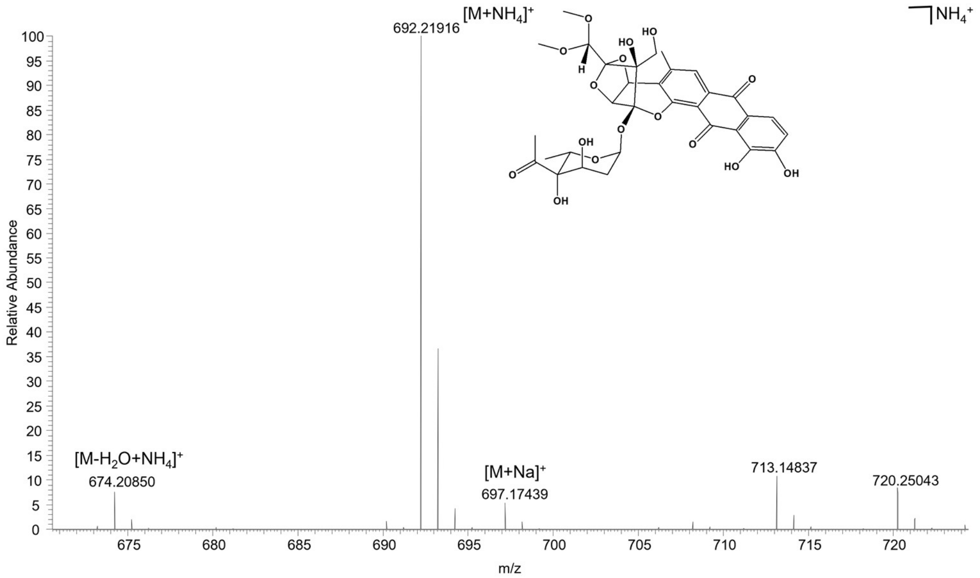

Figure S29. HR-ESI-MS of trioxacarcin 692 (**2**). Retention time: 27.5 min, Macherey-Nagel RP8 column (250 × 2 mm, 5 μm), LC-MS program 1.

ESI-MS/MS of trioxacarcin 692 (2)

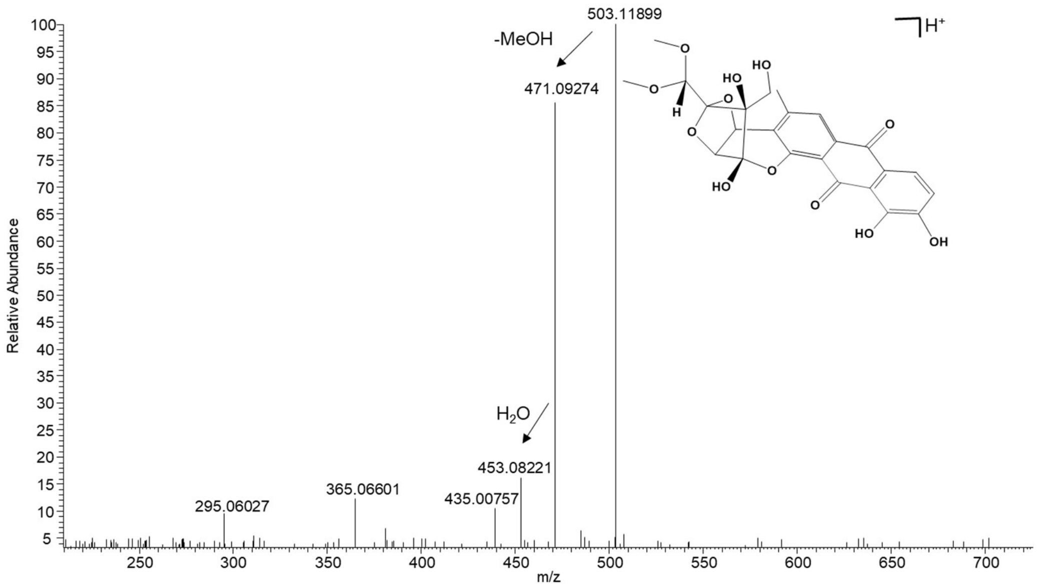

Figure S30. MS/MS fragmentation of trioxacarcin 692 (**2**). MS/MS of the quasimolecular ion *m/z* 692 of **2**.

^{1}H NMR spectrum of trioxacarcin 692 (2)

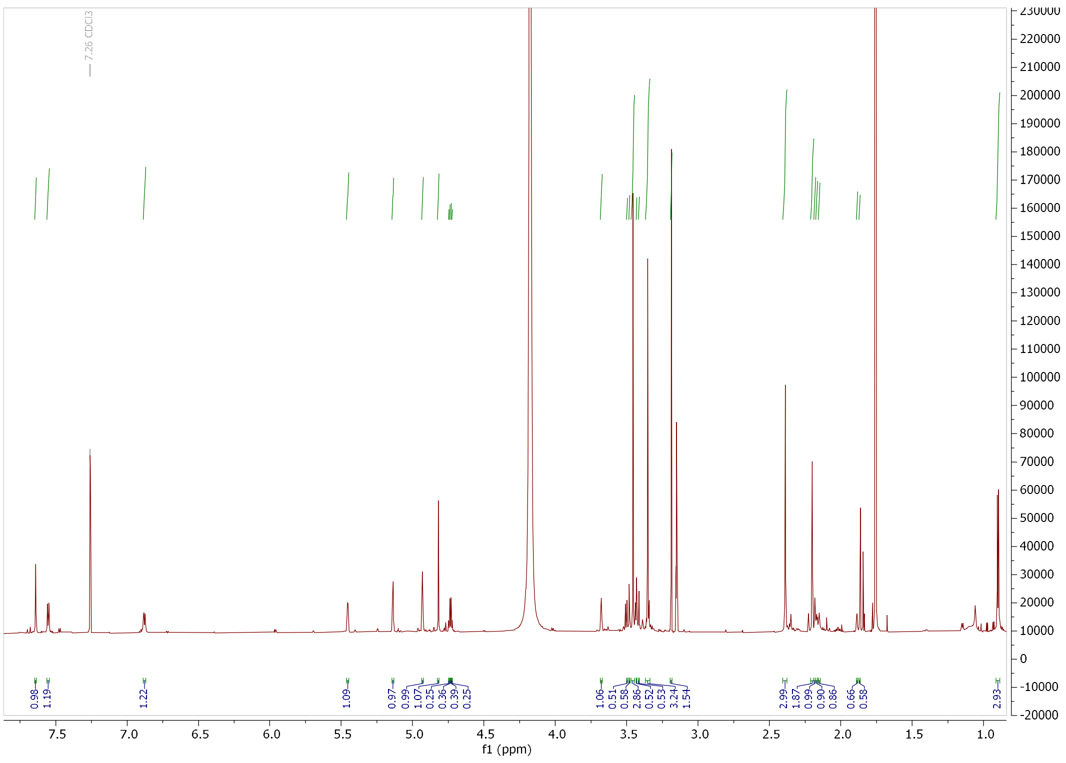

Figure S31. ^{1}H NMR spectrum (800 MHz, $CD_3OD/CDCl_3$) of trioxacarcin 692 (**2**).

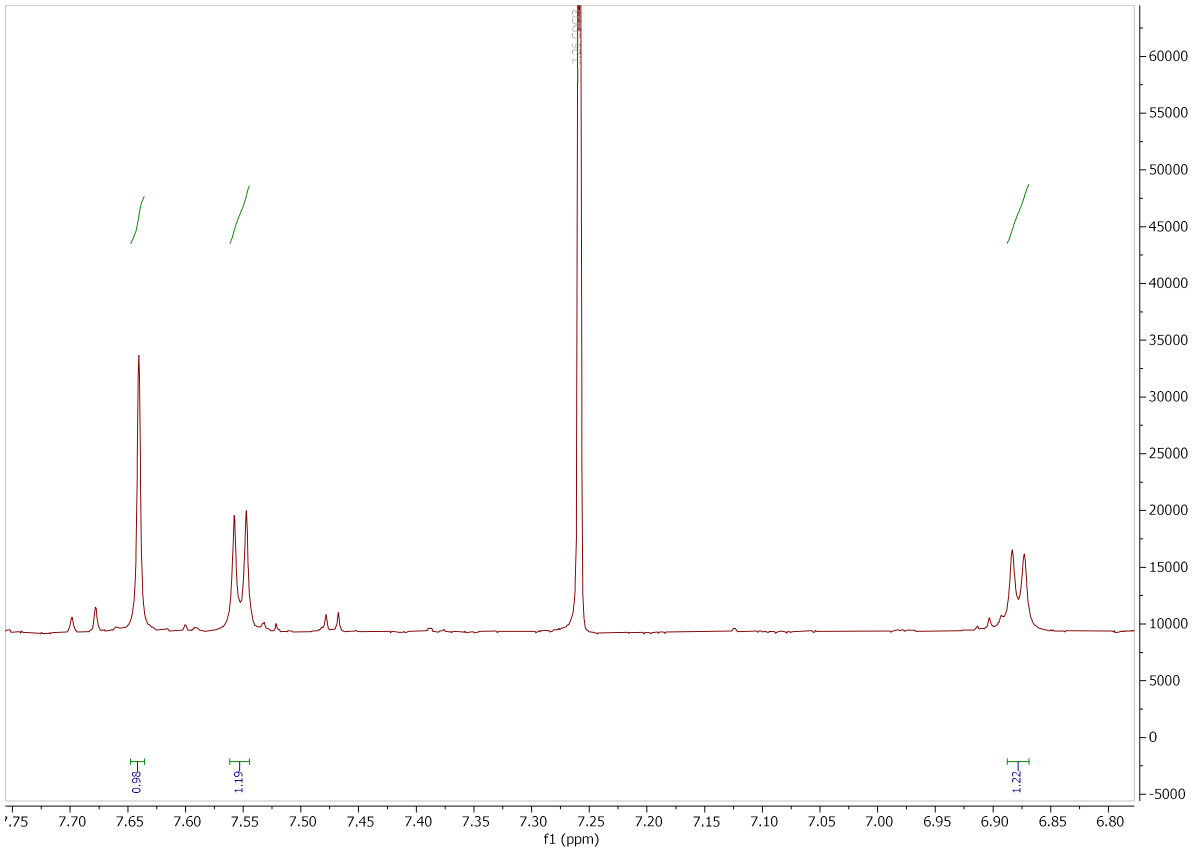

Figure S32. ^{1}H NMR spectrum (800 MHz, $CD_3OD/CDCl_3$) of trioxacarcin 692 (**2**), aromatic region.

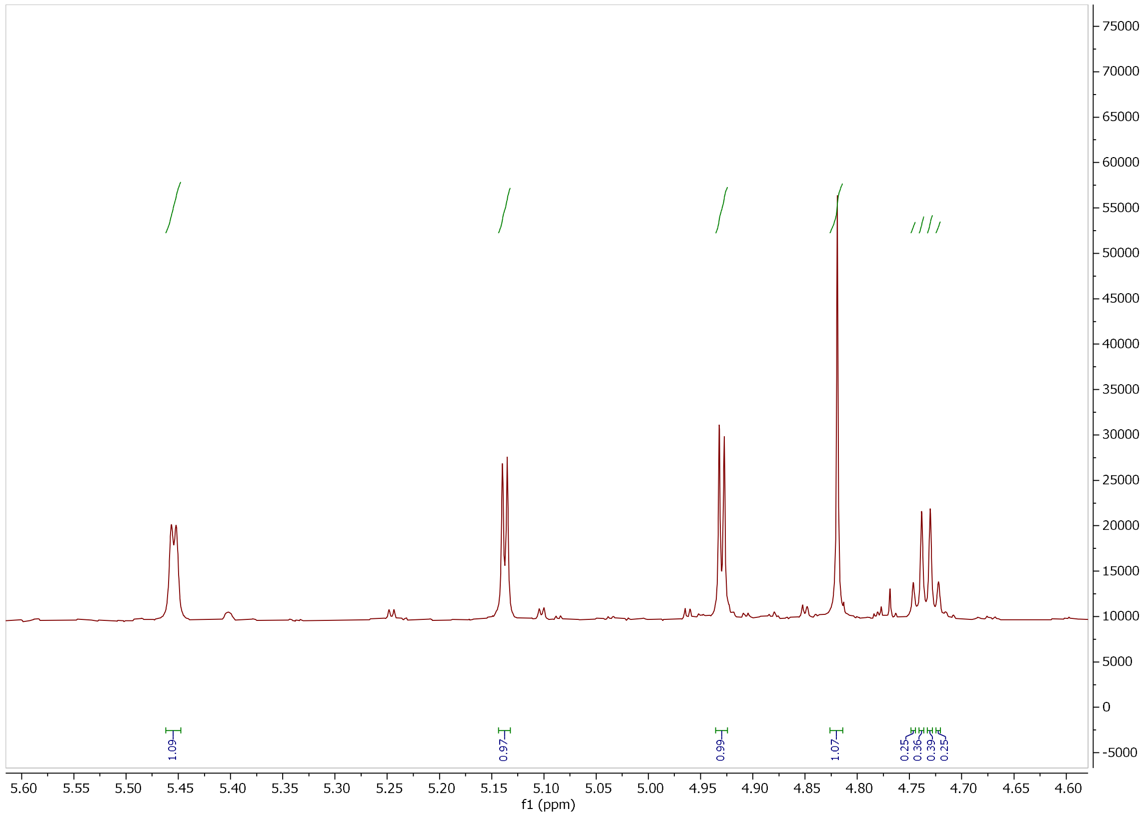

Figure S33. ^{1}H NMR spectrum (800 MHz, $CD_3OD/CDCl_3$) of trioxacarcin 692 (**2**), 4.5 –5.6 ppm.

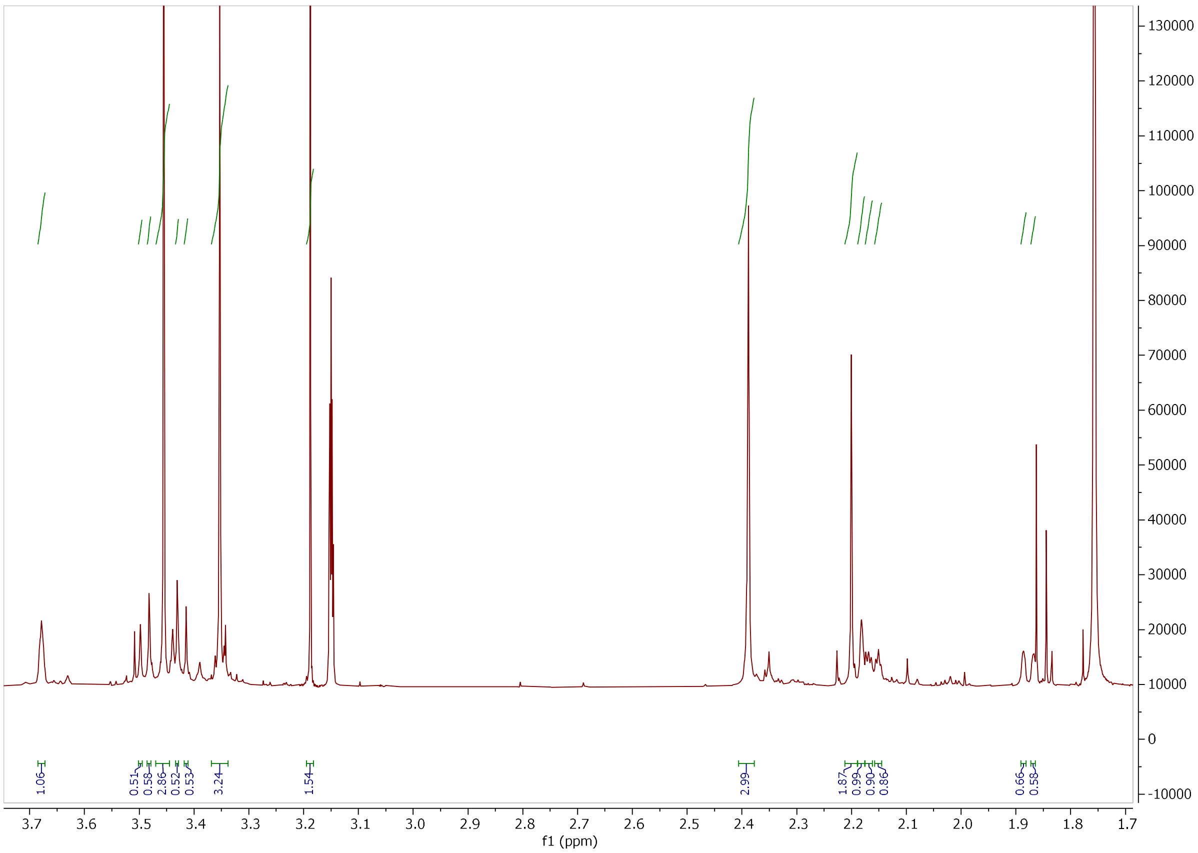

Figure S34. ^{1}H NMR spectrum (800 MHz, $CD_3OD/CDCl_3$) of trioxacarcin 692 (**2**), 1.7 –3.8 ppm.

^{1}H-^{1}H-COSY NMR spectrum of trioxacarcin 692 (2)

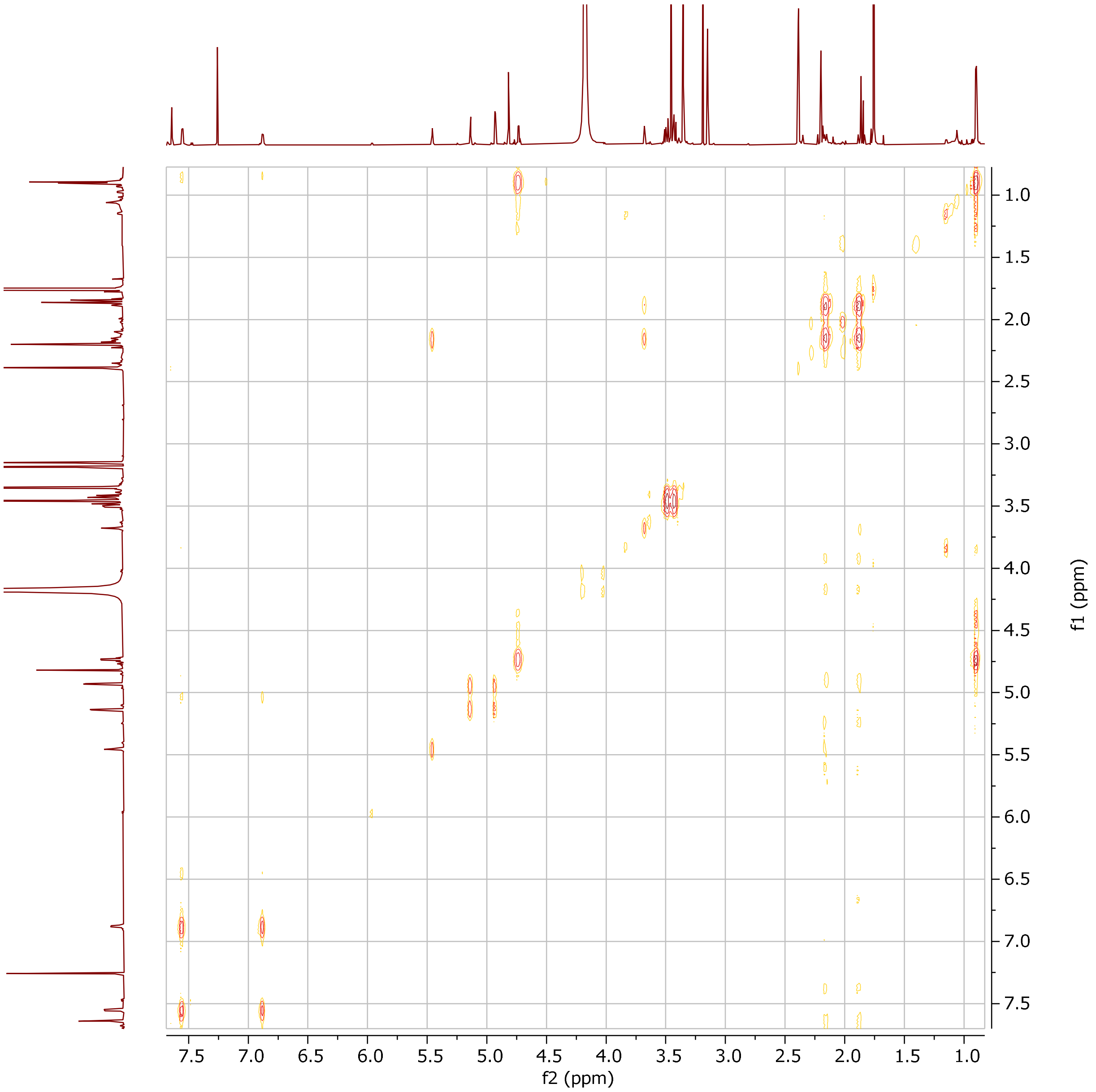

Figure S35. ^{1}H-^{1}H COSY NMR spectrum (800 MHz, $CD_3OD/CDCl_3$) of trioxacarcin 692 (**2**).

^{1}H-^{13}C-HSQC NMR spectrum of trioxacarcin 692 (2)

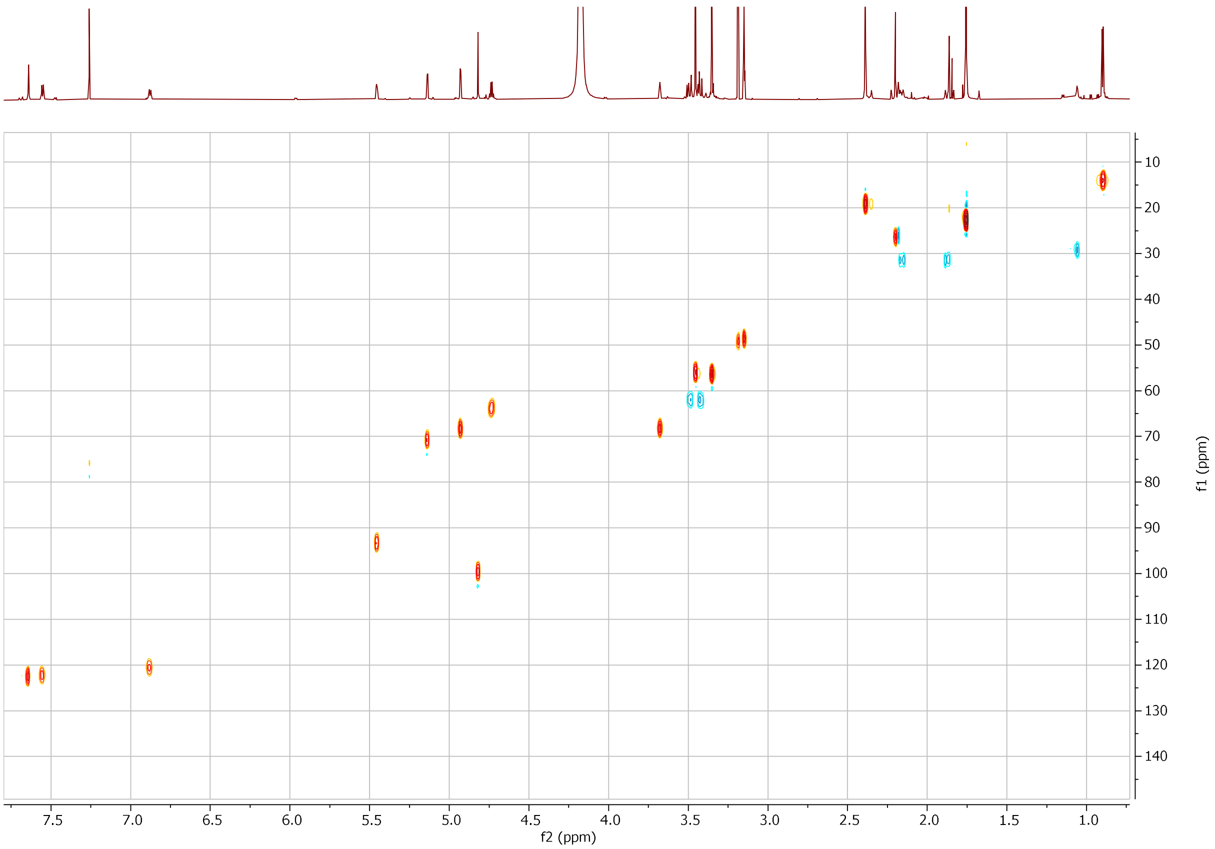

Figure S36. ^{1}H-^{13}C-HSQC NMR spectrum (800 MHz, $CD_3OD/CDCl_3$) of trioxacarcin 692 (**2**).

^{1}H-^{13}C-HMBC NMR spectrum of trioxacarcin 692 (2)

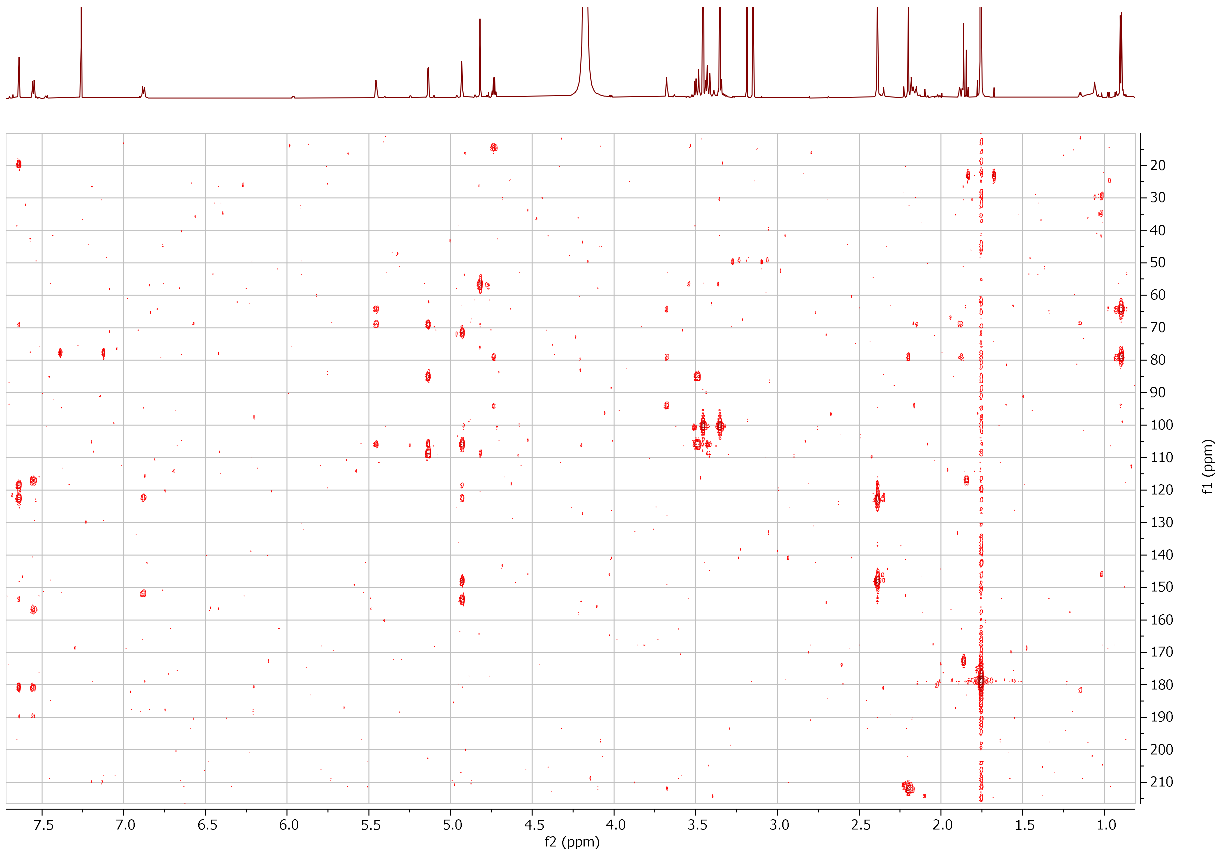

Figure S37. ^{1}H-^{13}C HMBC NMR spectrum (800 MHz, $CD_3OD/CDCl_3$) of trioxacarcin 692 (**2**).

Key ^{1}H-^{1}H COSY and ^{1}H-^{13}C HMBC correlations of trioxacarcin 692 (2)

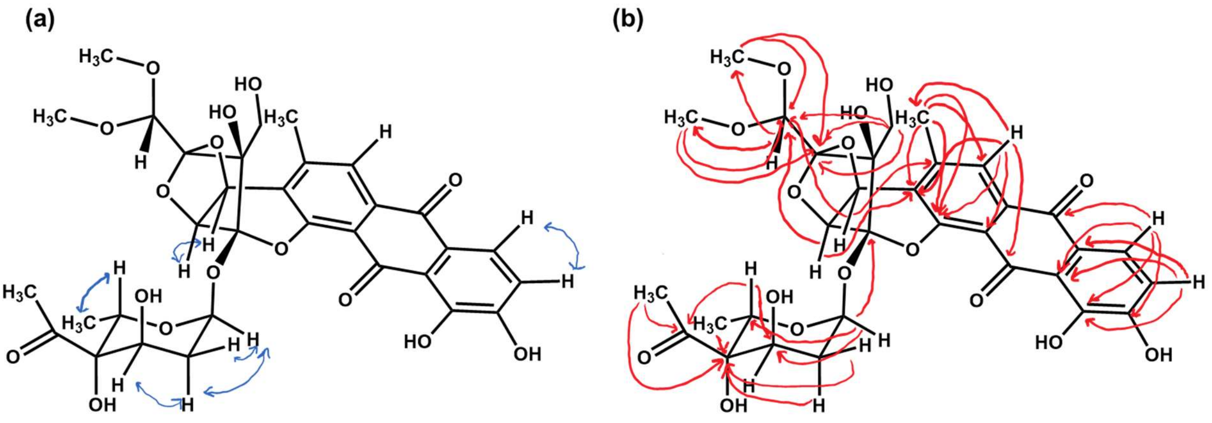

Figure S38. Key (**a**) ^{1}H-^{1}H COSY and (**b**) ^{1}H-^{13}C HMBC correlations of trioxacarcin 692 (**2**).

NMR data of trioxacarcin 692 (2)

1.6 mg from 12 l, bright yellow amorphous powder

Table S3. NMR data of trioxacarcin 692 (**2**) (800 MHz, $CD_3OD/CDCl_3$).

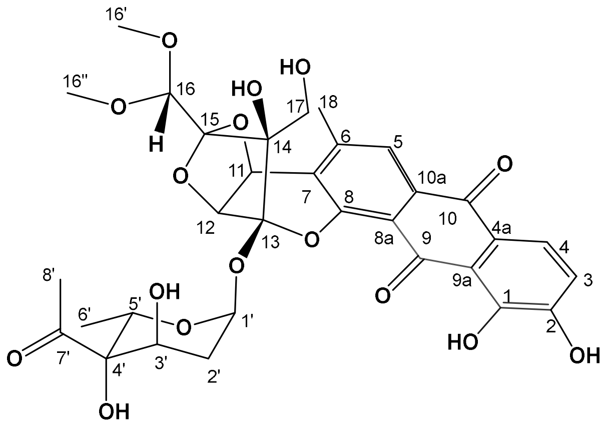

C atom number	δ ¹H/ppm (multiplicity, coupling constant *J*/Hz, integration)	δ ¹³C/ppm
1	-	151.73
2	-	157.18
3	6.94 (d, *J*=8.2, 1H)	120.60
4	7.49 (d, *J*=8.2, 1H)	122.32
4a	-	122.47
5	7.69 (s, 1H)	122.55
6	-	148.03
7	-	147.66
8	-	153.43
8a	-	118.40
9	-	189.47
9a	-	116.88
10	-	180.90
10a	-	118.30
11	4.93 (d, *J*=3.5, 1H)	68.58
12	5.14 (d, *J*=3.5, 1H)	71.29
13	-	105.51
14	-	85.01
15	-	104.56
16	4.82 (s, 1H)	99.95
16′	3.35 (s, 3H)	56.68
16′′	3.46 (s, 3H)	56.36
17	3.42 (d, *J*=12.9, 1H), 3.49 (d, *J*=12.9, 1H)	62.25
18	2.39 (s, 3H)	19.48
1′	5.45 (d, *J*=3.6, 1H)	93.68
2′	1.86-1.89 (m, 1H), 2.14-2.18 (m, 1H)	31.76
3′	3.67-3.69 (m, 1H)	68.52
4′	-	78.76
5′	4.73 (q, *J*=6.5, 1H)	64.14
6′	0.90 (d, *J*=6.5, 3H)	14.35
7′	-	212.75
8′	2.20, 2.18 enol (s, 3H)	26.51

Production of trioxacarcin 780 (1) in modified R5A medium

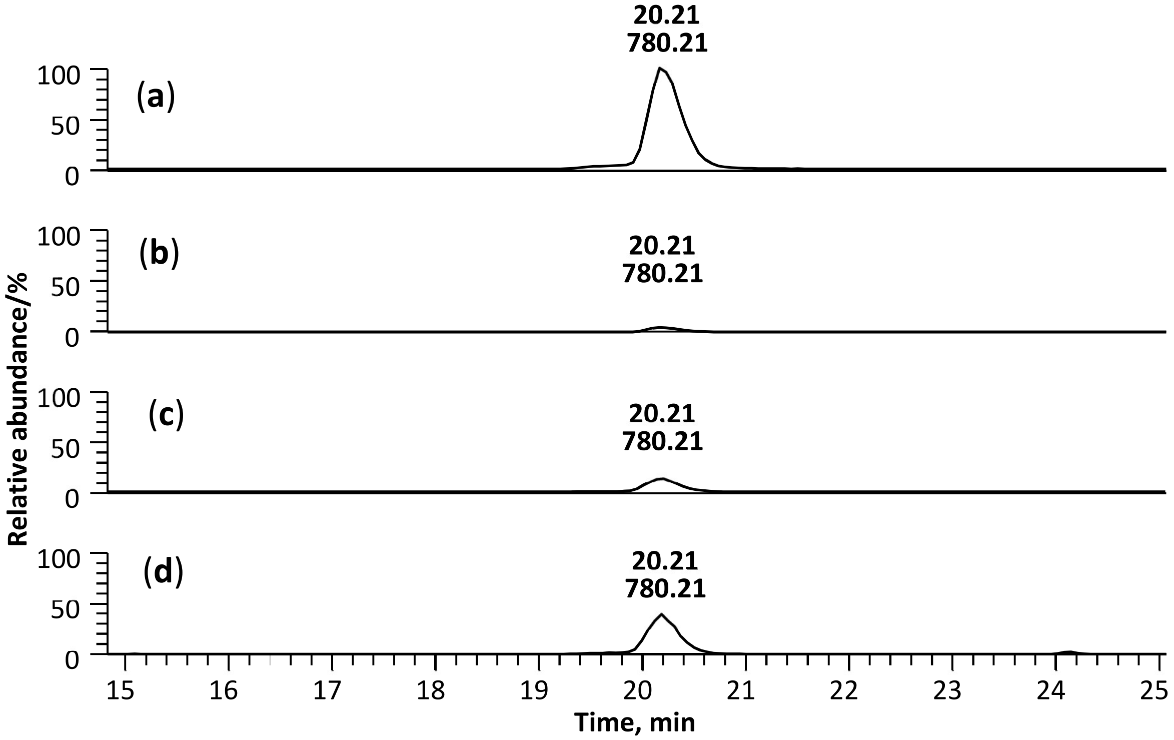

Figure S39. Detection of trioxacarcin 780 (**1**) in the processed supernatant of 21 d *Streptomyces* sp. 127Q culture. (**a**) Unmodified/regular R5A medium; (**b**) modified R5A medium containing 5 g/l of casamino acids, 0 g/l yeast extract; (**c**) modified R5A medium containing 5 g/l of casamino acids, 0 g/l yeast extract, supplemented with 2 mM nicotinic acid; (**d**) modified R5A medium containing 5 g/l of casamino acids, 0 g/l yeast extract, supplemented with 2 mM tryptophan. LC-MS program 1, Macherey-Nagel RP8 column (250 × 2 mm, 5 μm).

LC-MS chromatogram of *Streptomyces* sp. 127Q crude extract: formation of trioxacarcin and gutingimycin compounds

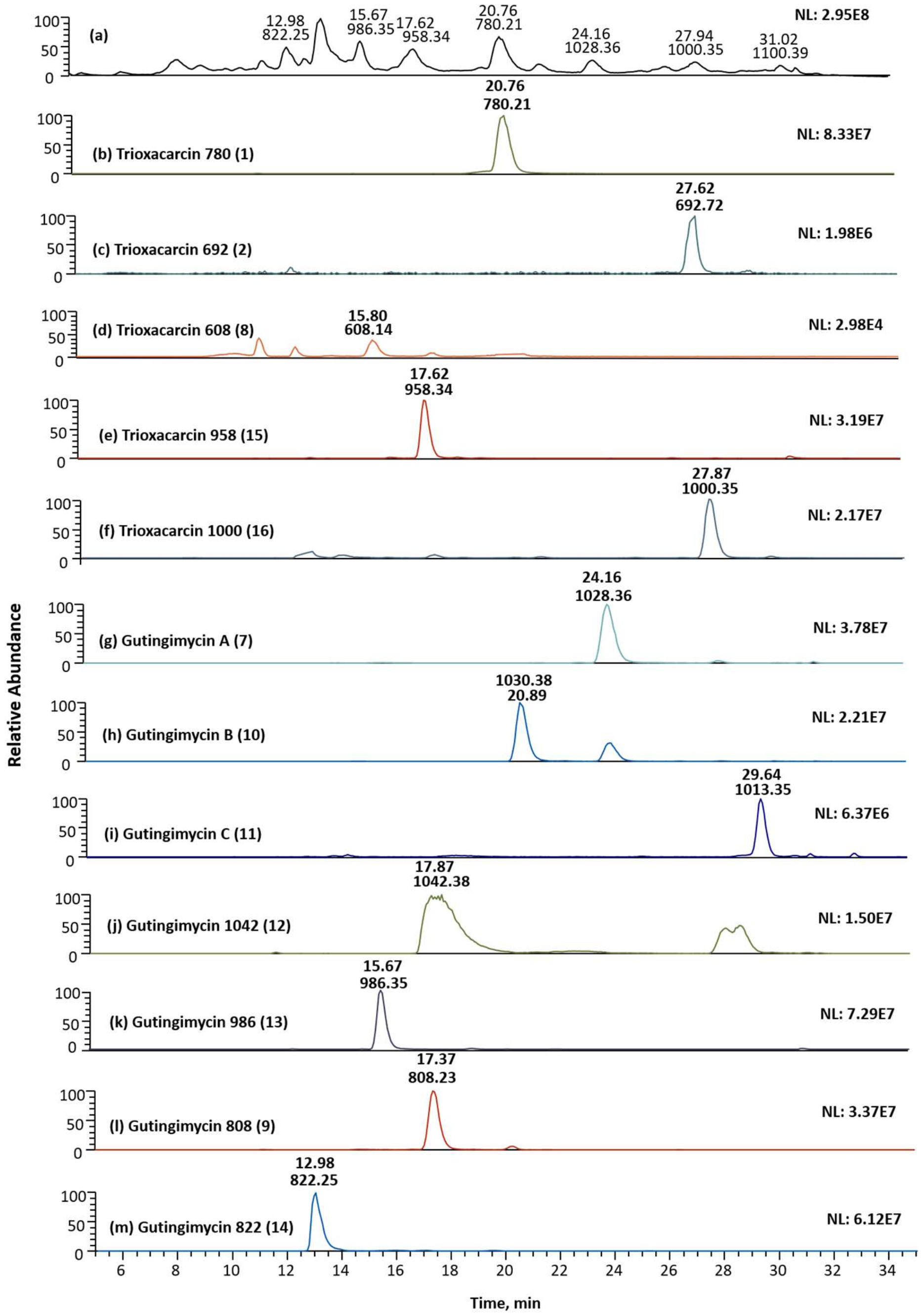

Figure S40. Relative production of trioxacarcin and gutingimycin compounds by *Streptomyces* sp. 127Q. (**a**) Total ion chromatogram of crude extract obtained after HP20 chromatography, prepared from a 4 l fermentation scale of 21 d old culture. A volume of 10 µl (corresponding to ca. 4.35 µg/µl) was injected into LC-MS. Extracted ion chromatograms (EICs) are shown for selected metabolites: (**b**) EIC of trioxacarcin 780 (**1**); (**c**) EIC of trioxacarcin 692 (**2**); (**d**) EIC of trioxacarcin 608 (**8**); (**e**) EIC of trioxacarcin 958 (**15**); (**f**) EIC of trioxacarcin 1000 (**16**); (**g**) EIC of gutingimycin A (**7**); (**h**) EIC of gutingimycin B (**10**); (**i**) EIC of gutingimycin C (**11**); (**j**) EIC of gutingimycin 1042 (**12**); (**k**) EIC of gutingimycin 986 (**13**); (**l**) EIC of gutingimycin 808 (**9**); (**m**) EIC of gutingimycin 822 (**14**).

Molecular networking analysis of trioxacarcin and gutingimycin compounds of *Streptomyces* sp. 127Q

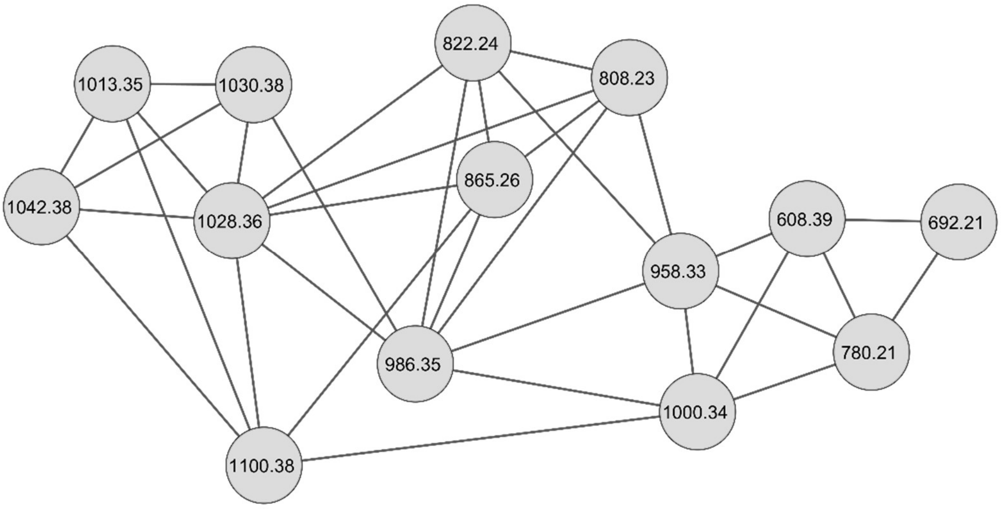

Figure S41. Molecular networking of trioxacarcins and gutingimycins detected in Diaion HP20 eluate, prepared from 4 l of 21 d old *Streptomyces* sp. 127Q culture. The molecular network was generated using Global Natural Products Social (GNPS) molecular networking analysis of the secondary metabolites (GNPS; http://gnps.ucsd.edu).

Gutingimycin 808 (9)

UV-Vis spectrum of gutingimycin 808 (9)

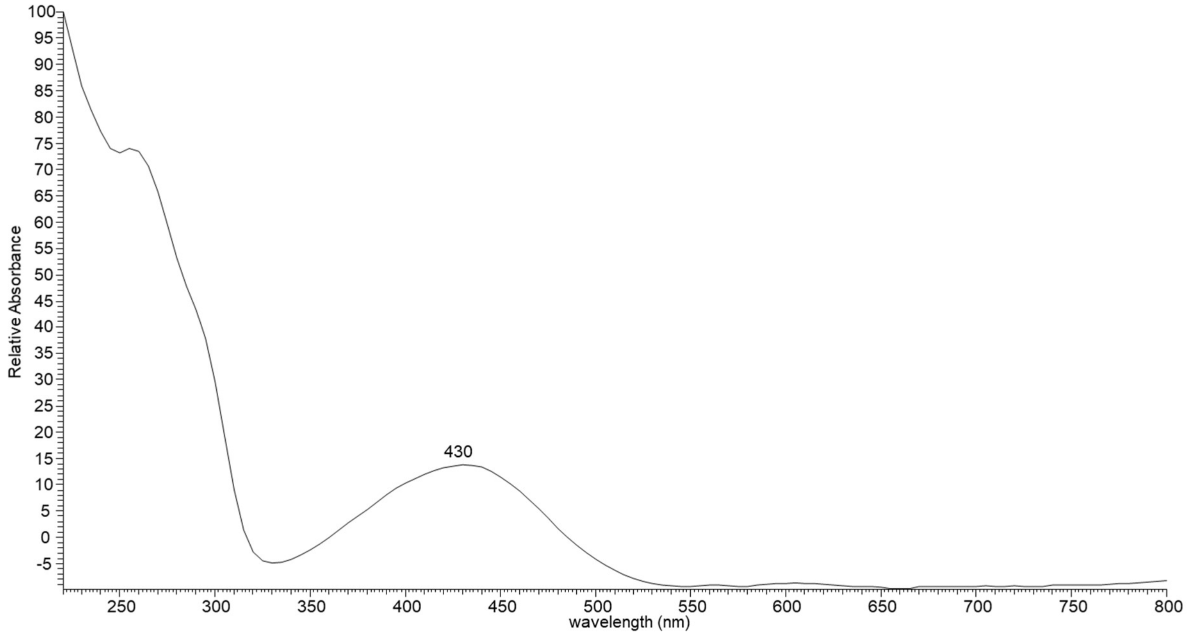

Figure S42. UV-Vis spectrum of gutingimycin 808 (**9**).

HR-ESI-MS of gutingimycin 808 (9)

m/z	Theo. Mass	Δppm	RDB equiv.	Composition
808.23064	808.23081	-0.21	21.5	$C_{37}H_{38}O_{16}N_5$

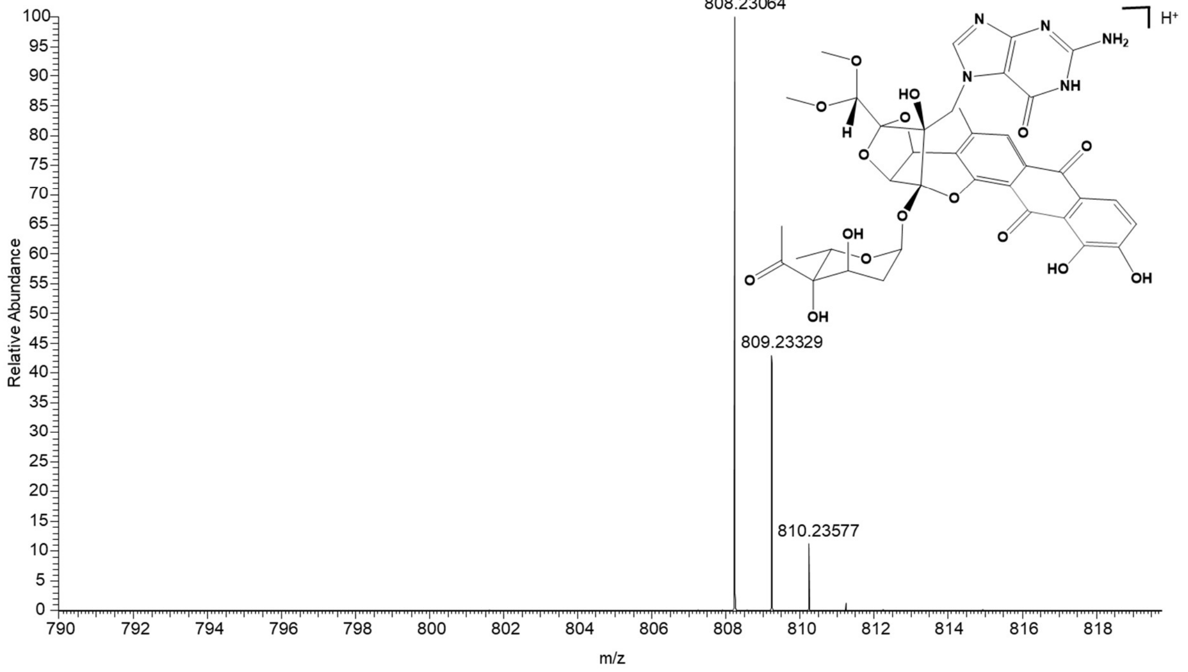

Figure S43. HR-ESI-MS of trioxacarcin 808 (**9**). Retention time 17.3 min, Macherey-Nagel RP8 column (250 × 2 mm, 5 µm), LC-MS program 1.

ESI-MS/MS of gutingimycin 808 (9)

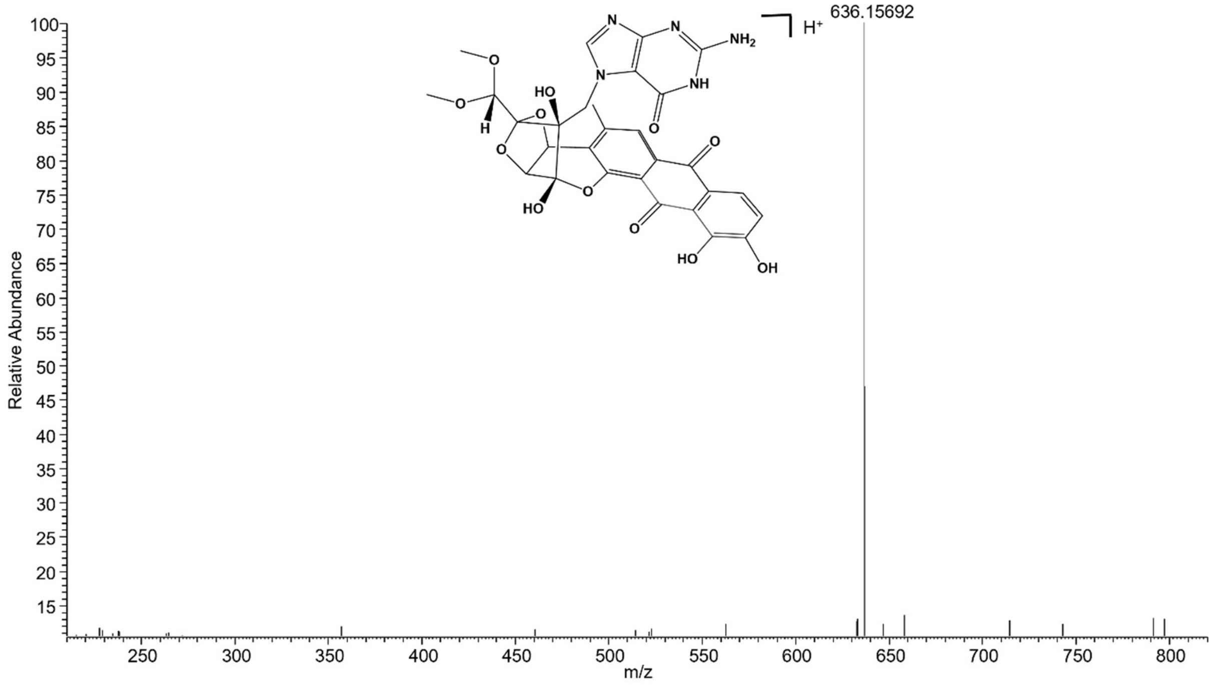

Figure S44. HR-ESI-MS/MS fragmentation of gutingimycin 808 (**9**). MS/MS of the quasimolecular ion *m*/*z* 808 of **9**.

Gutingimycin B (10)

UV-Vis spectrum of gutingimycin B (10)

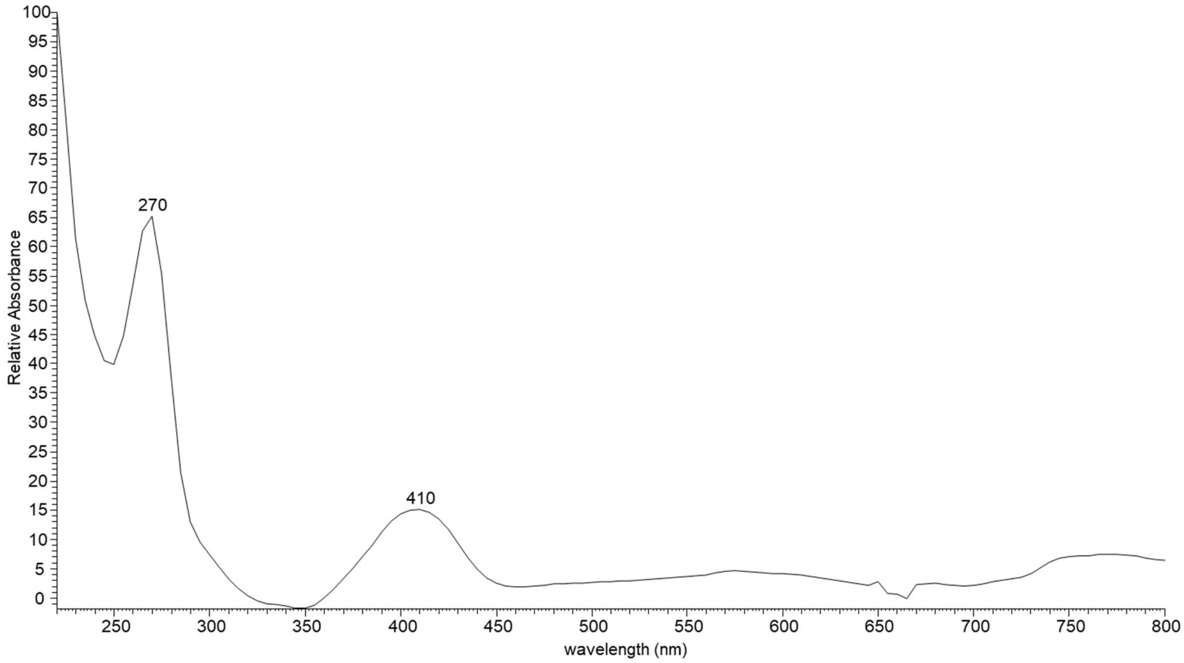

Figure S45. UV-Vis spectrum of gutingimycin B (**10**).

HR-ESI-MS of gutingimycin B (10)

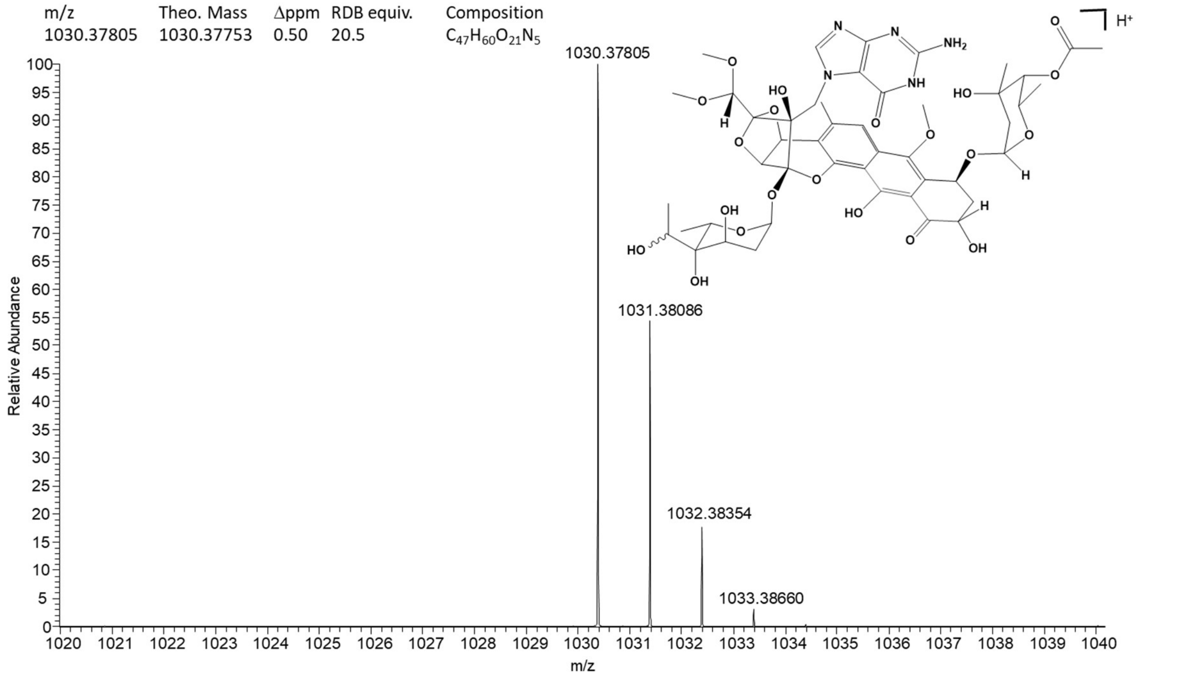

Figure S46. HR-ESI-MS of gutingimycin B (**10**). Retention time 20.9 min, Macherey-Nagel RP8 column (250 × 2 mm, 5 µm), LC-MS program 1.

ESI-MS/MS of gutingimycin B (10)

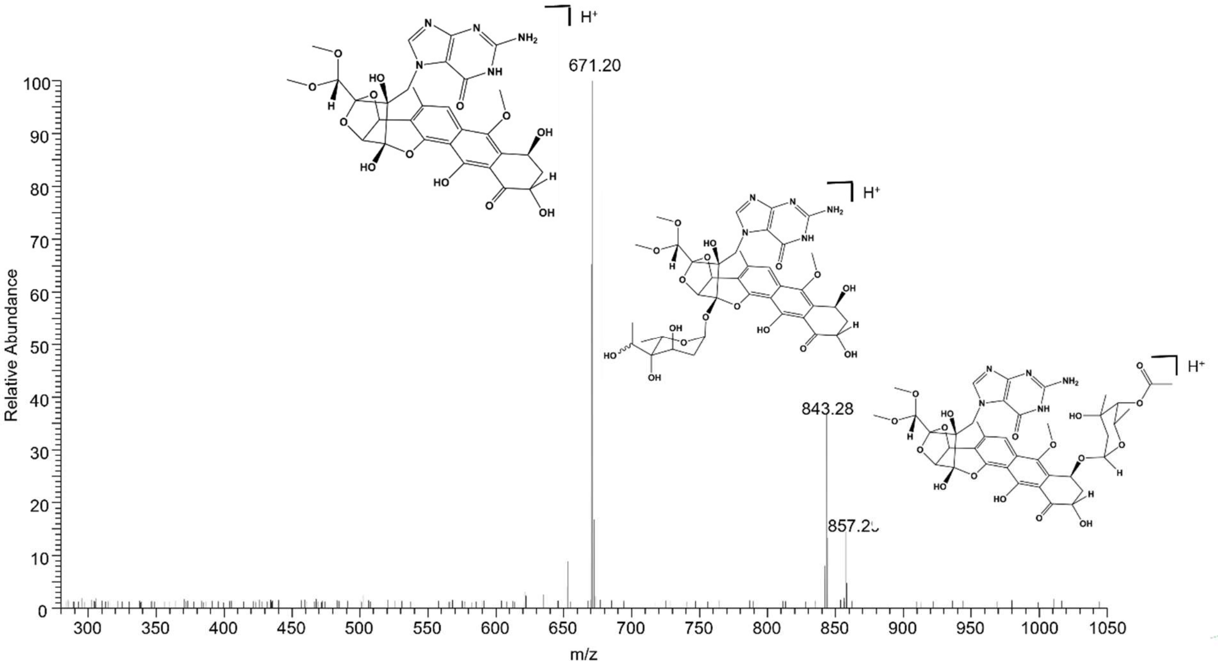

Figure S47. HR-ESI-MS/MS fragmentation of gutingimycin B (**10**). MS/MS of the quasimolecular ion *m*/*z* 1030 of **10**.

Gutingimycin C (11)

UV-Vis spectrum of gutingimycin C (11)

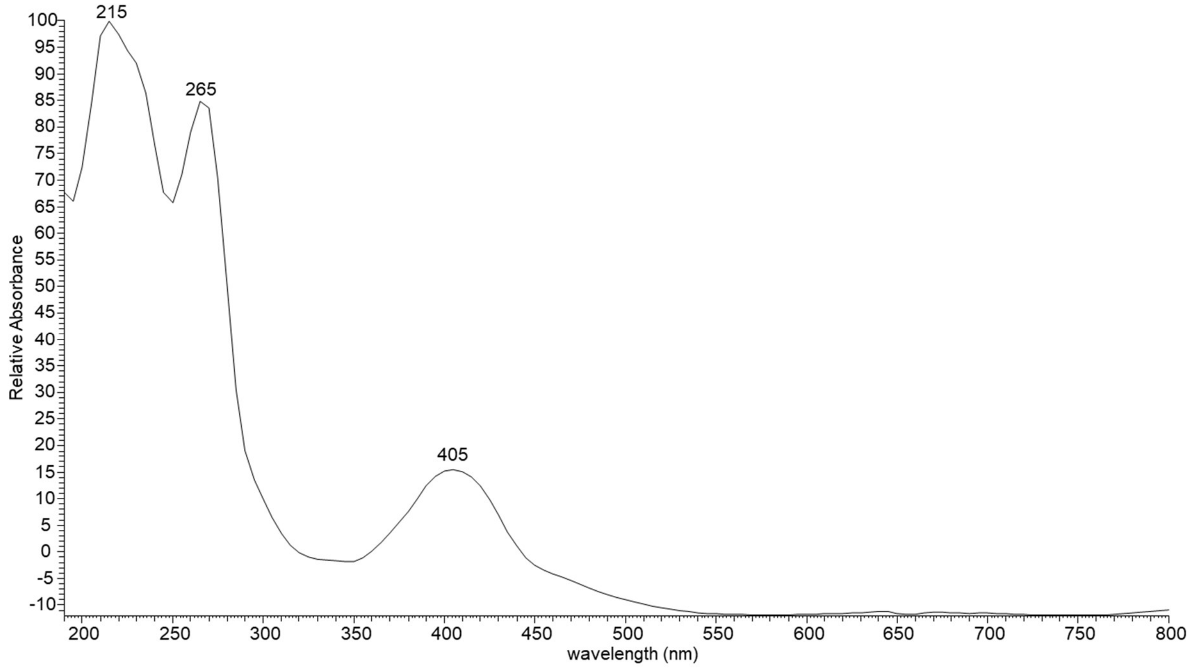

Figure S48. UV-Vis spectrum of gutingimycin C (**11**).

HR-ESI-MS of gutingimycin C (11)

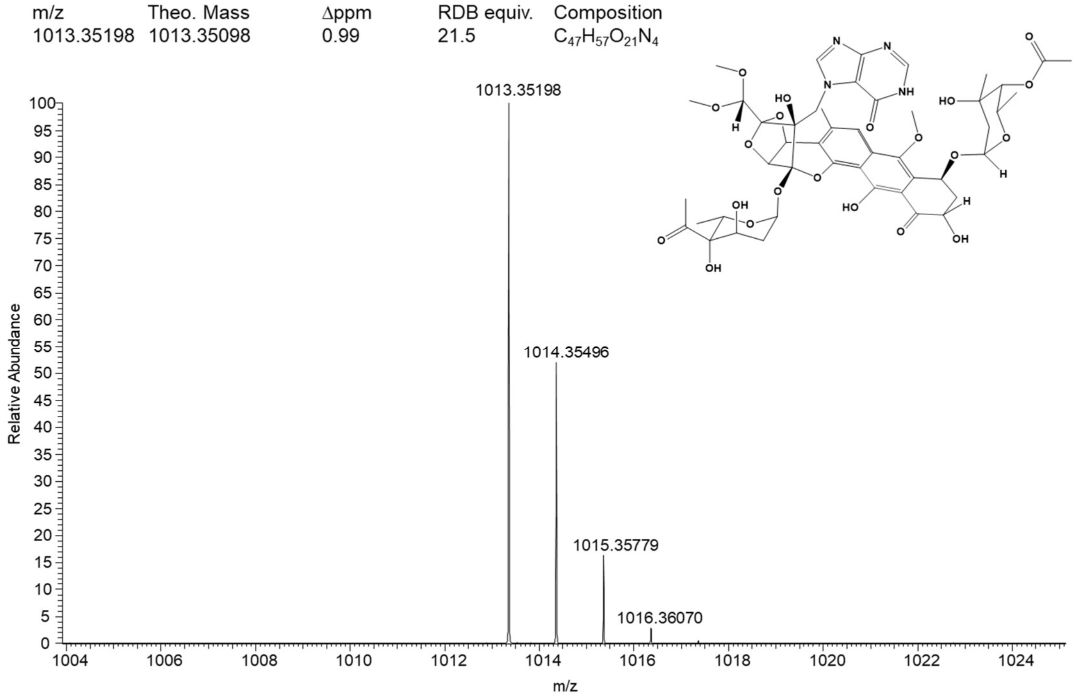

Figure S49. HR-ESI-MS of gutingimycin C (**11**). Retention time 29.6 min, Macherey-Nagel RP8 column (250 × 2 mm, 5 µm), LC-MS program 1.

ESI-MS/MS of gutingimycin C (11)

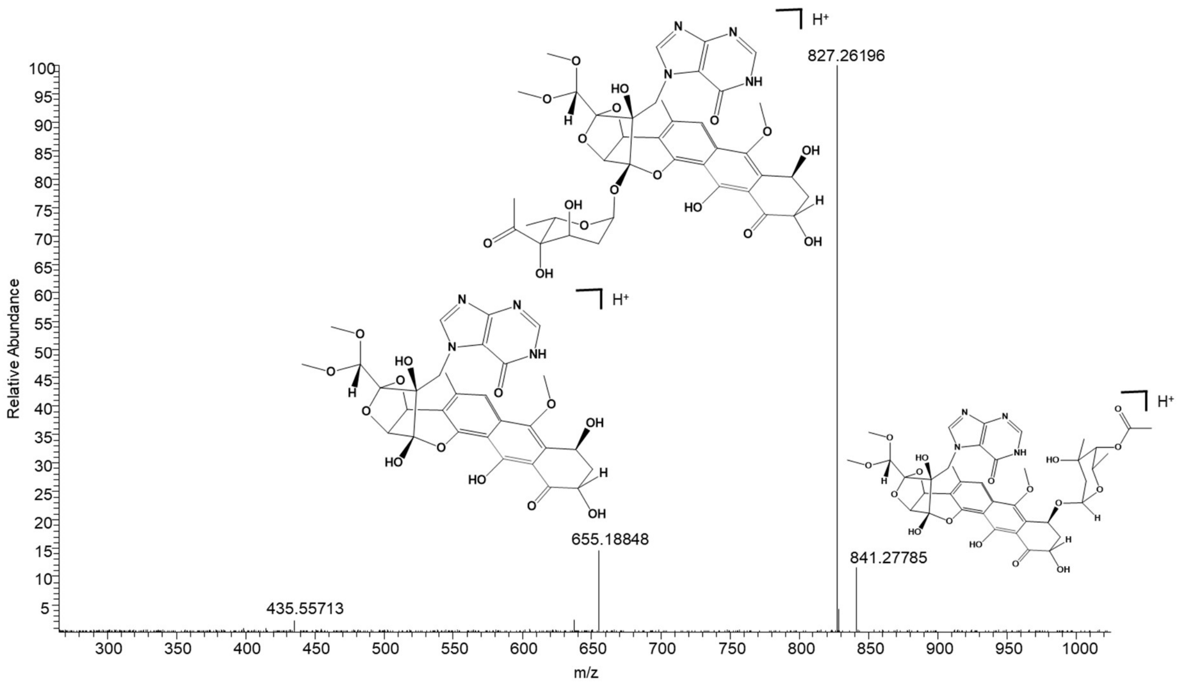

Figure S50. HR-ESI-MS/MS fragmentation of gutingimycin C (**11**). MS/MS of the quasimolecular ion *m*/*z* 1013 of **11**.

Gutingimycin 1042 (12)

UV-Vis spectrum of gutingimycin 1042 (12)

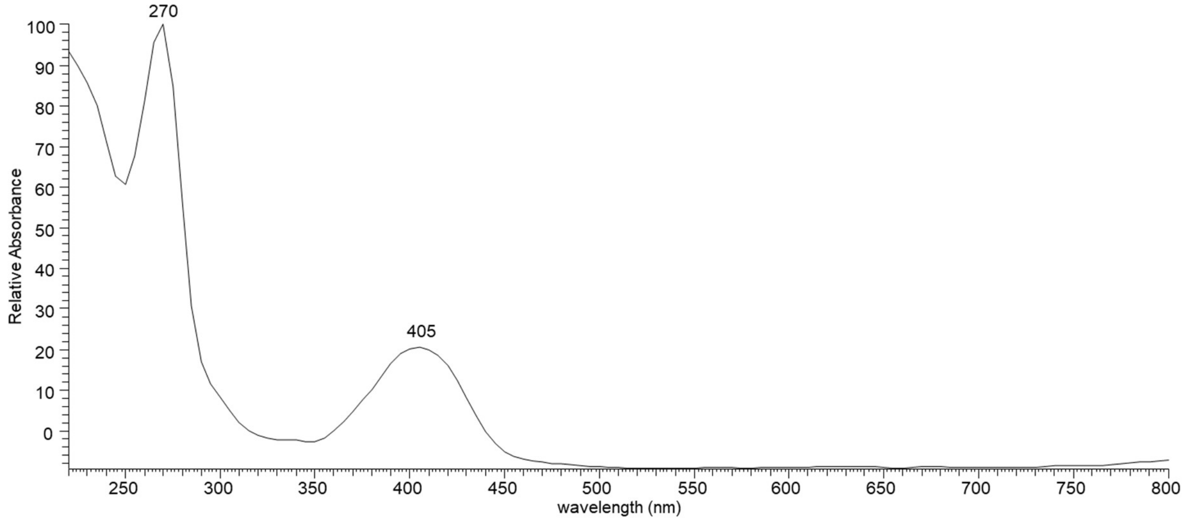

Figure S51. UV-Vis spectrum of gutingimycin 1042 (**12**).

HR-ESI-MS of gutingimycin 1042 (12)

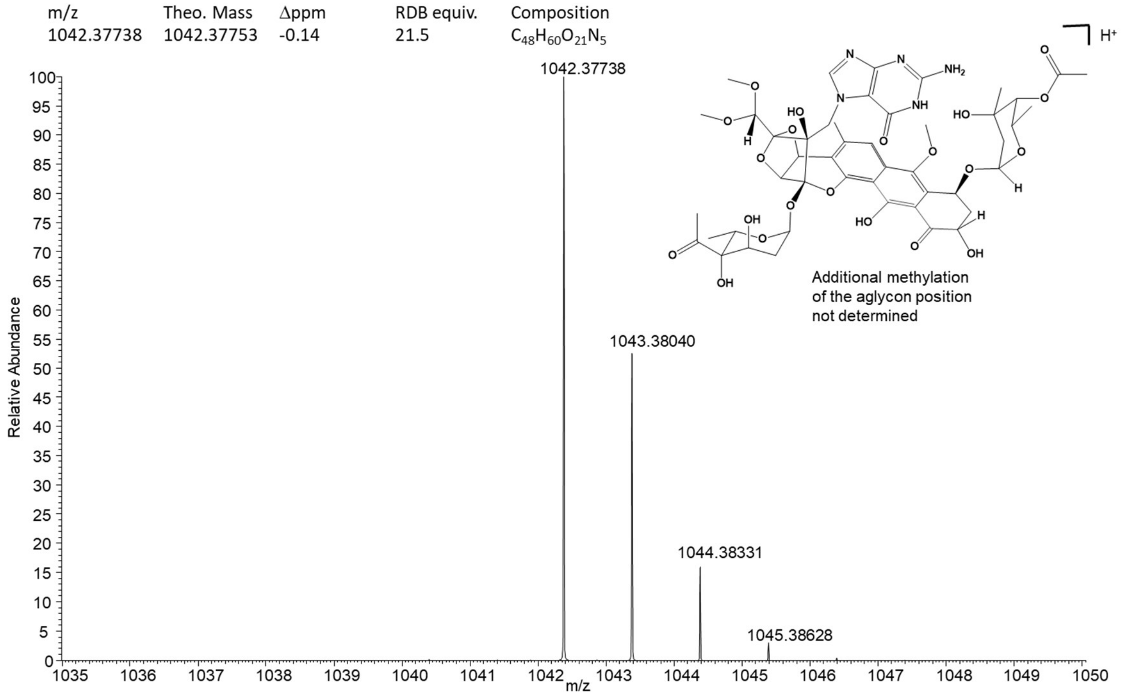

Figure S52. HR-ESI-MS of gutingimycin 1042 (**12**). Retention time 28.7 min, Macherey-Nagel RP8 column (250 × 2 mm, 5 µm), LC-MS program 1.

ESI-MS/MS of gutingimycin 1042 (12)

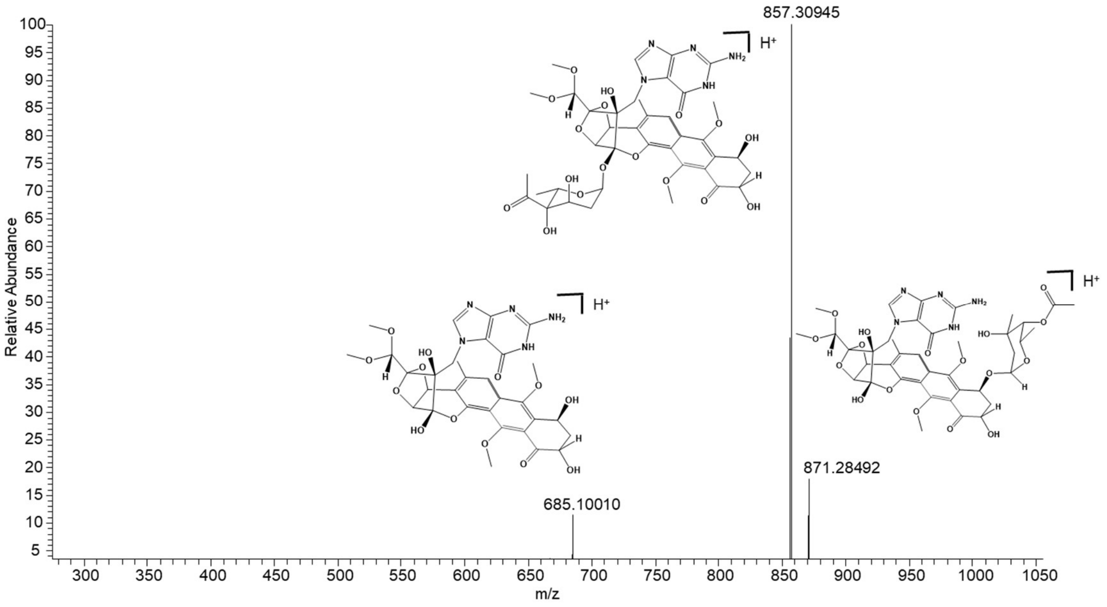

Figure S53. HR-ESI-MS/MS fragmentation of gutingimycin 1042 (**12**). MS/MS of the quasimolecular ion *m*/*z* 1042 of **12**. The position of the additional methyl group in the aglycon was not determined.

Gutingimycin 986 (13)

UV-Vis spectrum of gutingimycin 986 (13)

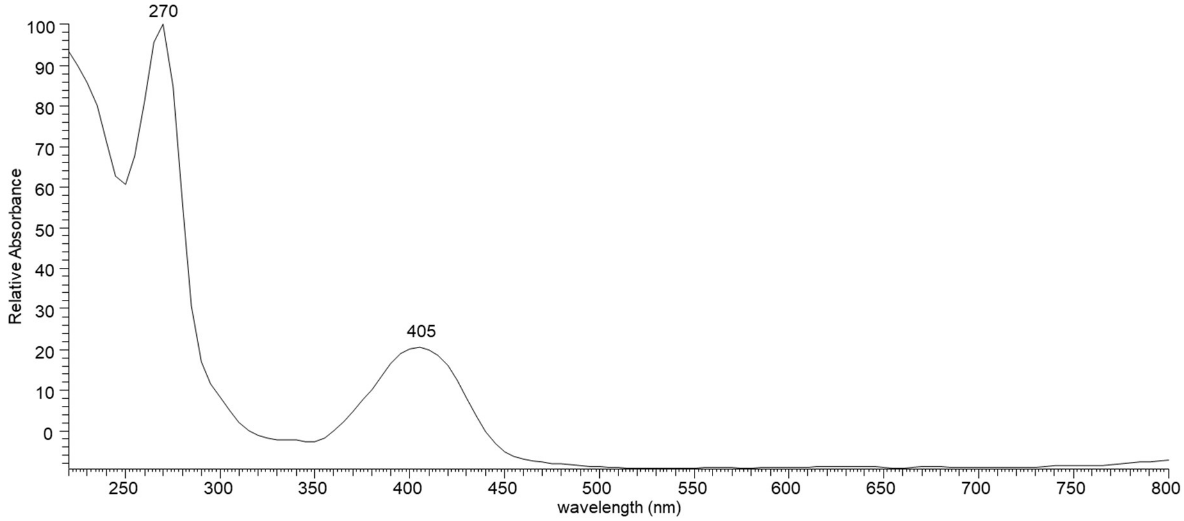

Figure S54. UV-Vis spectrum of gutingimycin 986 (**13**).

HR-ESI-MS of gutingimycin 986 (13)

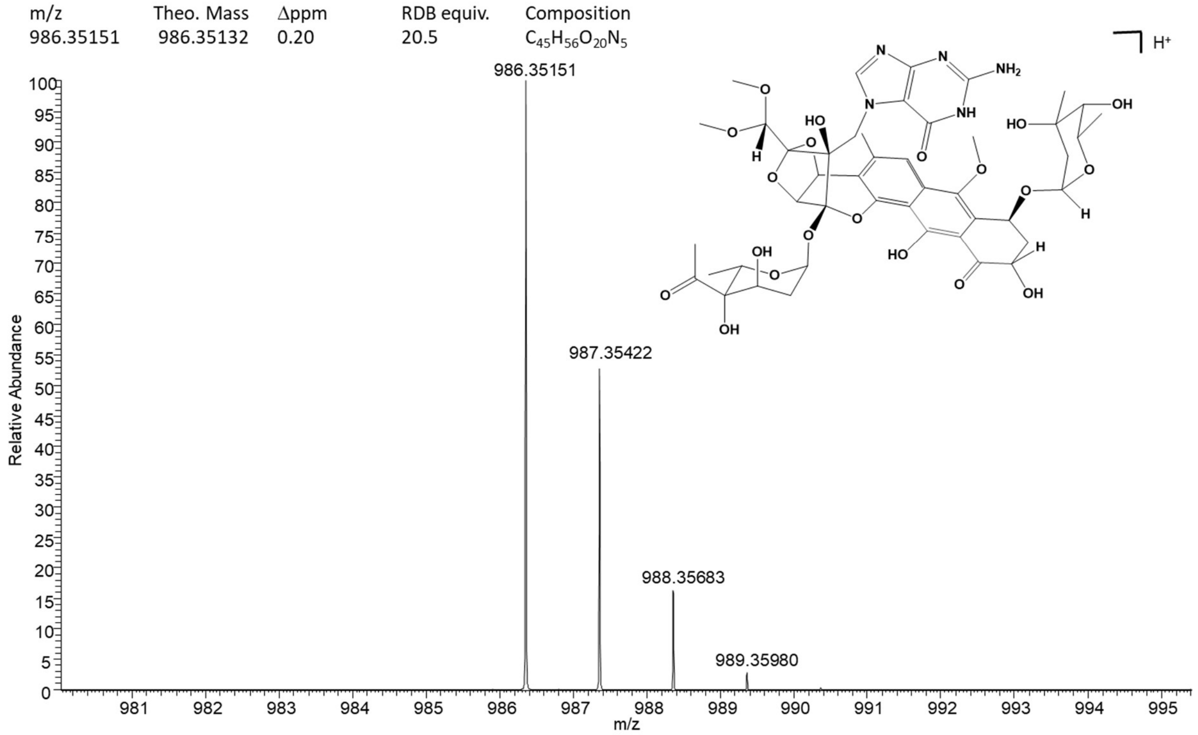

Figure S55. HR-ESI-MS of trioxacarcin 986 (**13**). Retention time 15.6 min, Macherey-Nagel RP8 column (250 × 2 mm, 5 µm), LC-MS program 1.

ESI-MS/MS of gutingimycin 986 (13)

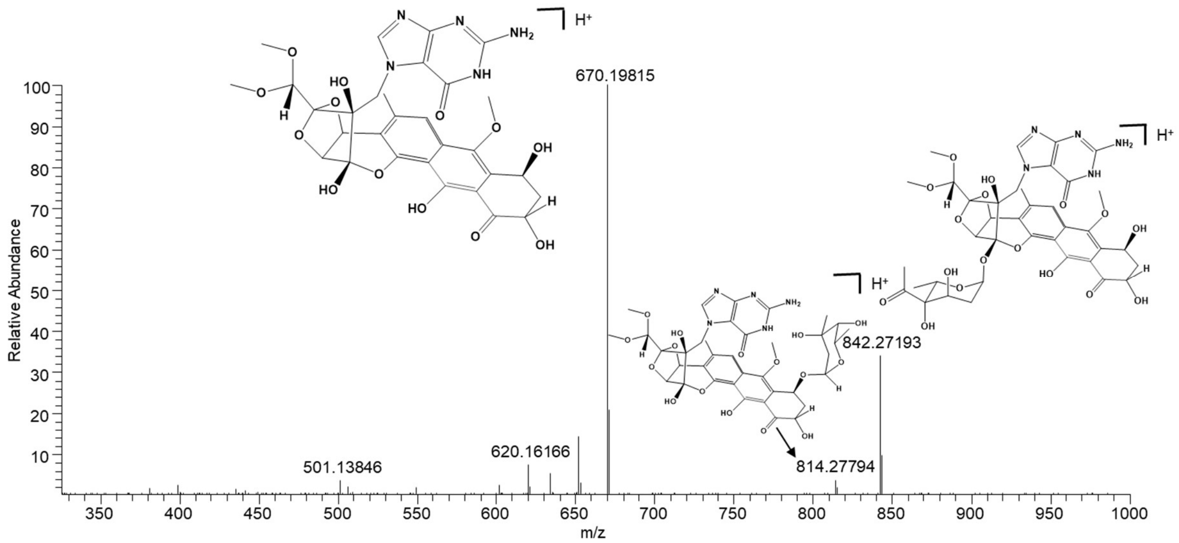

Figure S56. HR-ESI-MS/MS fragmentation of gutingimycin 986 (**13**). MS/MS of the quasimolecular ion *m*/*z* 986 of **13**.

Gutingimycin 822 (14)

UV-Vis spectrum of gutingimycin 822 (14)

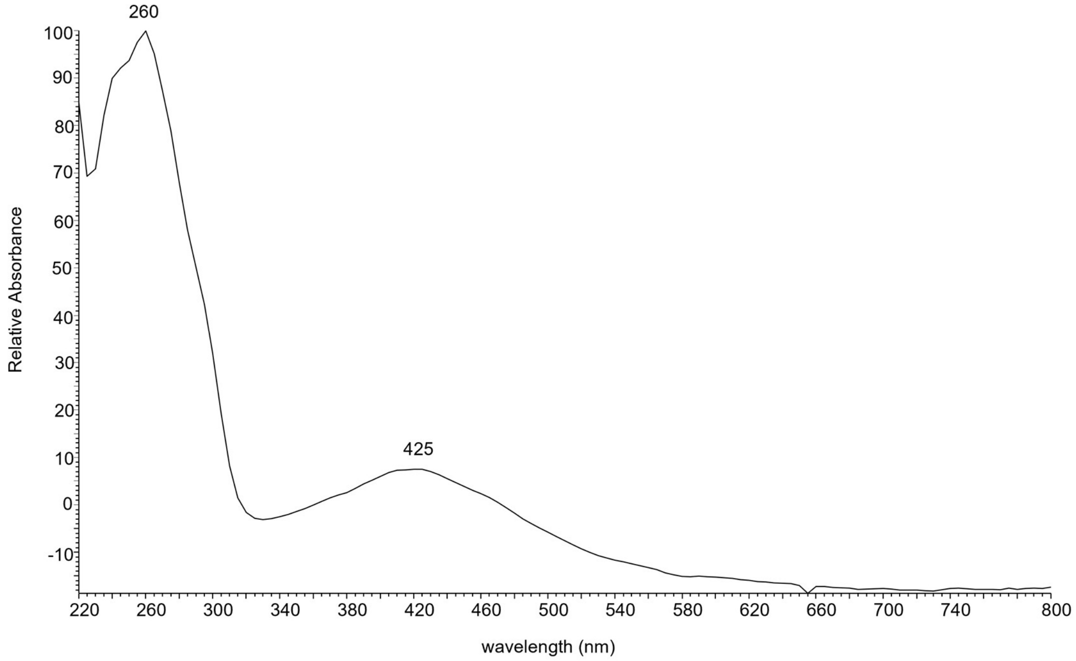

Figure S57. UV-Vis spectrum of gutingimycin 822 (**14**).

HR-ESI-MS of gutingimycin 822 (14)

m/z	Theo. Mass	Δppm	RDB equiv.	Composition
822.24561	822.24701	-1.029	21.5	$C_{38}H_{40}O_{16}N_5$

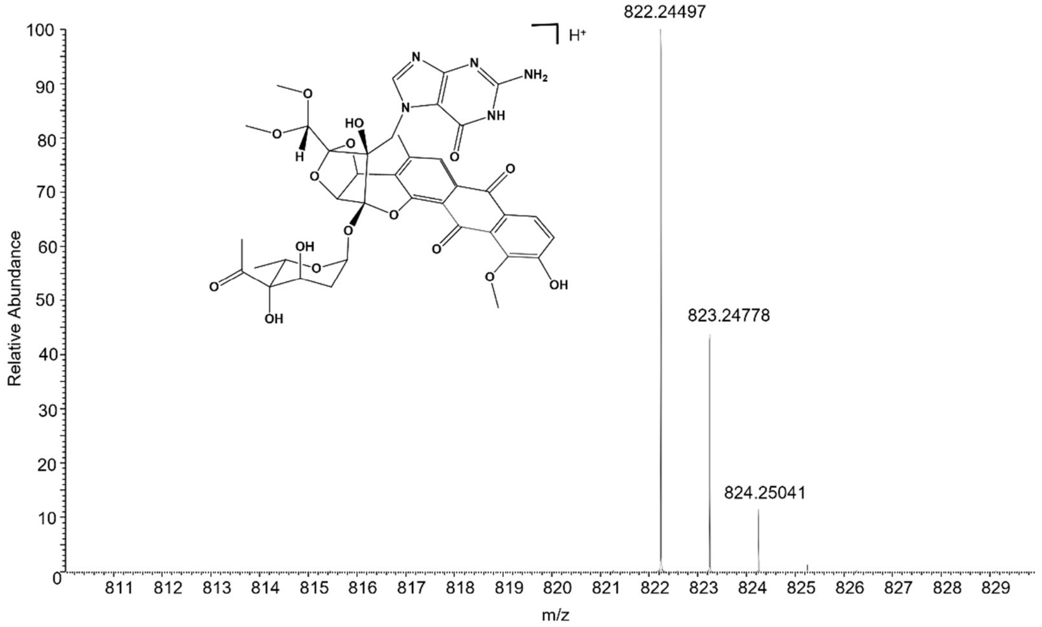

Figure S58. HR-ESI-MS of trioxacarcin 822 (**14**). Retention time 12.9 min, Macherey-Nagel RP8 column (250 × 2 mm, 5 µm), LC-MS program 1.

ESI-MS/MS of gutingimycin 822 (14)

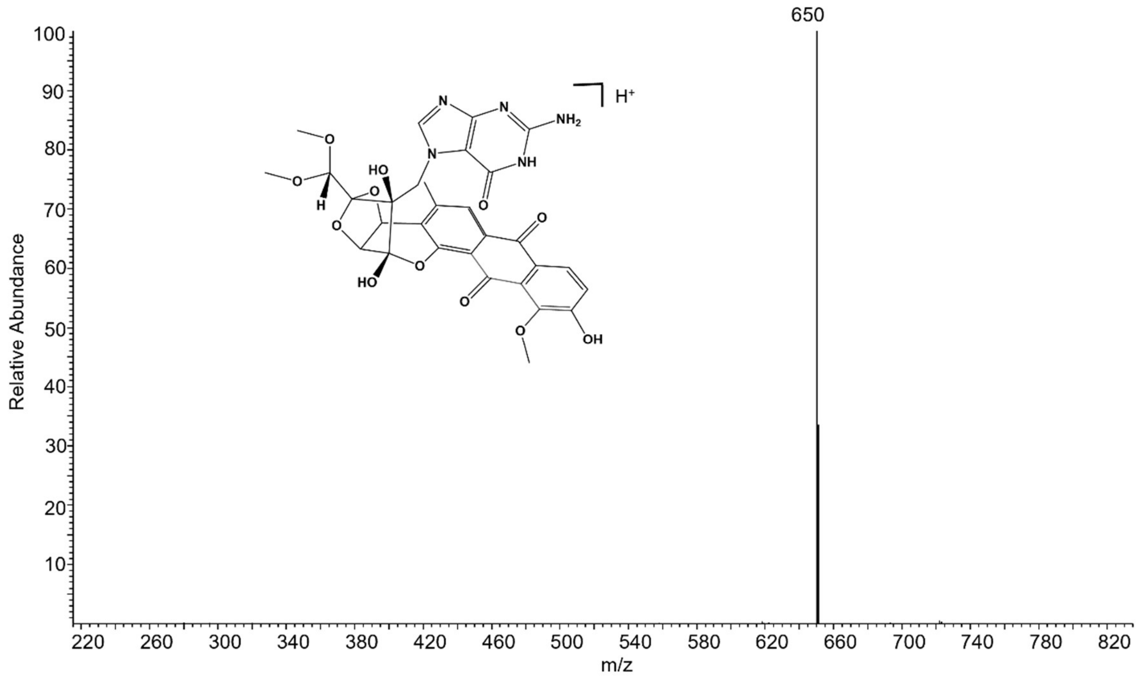

Figure S59. HR-ESI-MS/MS fragmentation of gutingimycin 822 (**14**). MS/MS of the quasimolecular ion *m*/*z* 822 of **14**. The position of the additional methyl group in the aglycon was not determined.

Trioxacarcin 958 (15)

UV-Vis spectrum of trioxacarcin 958 (15)

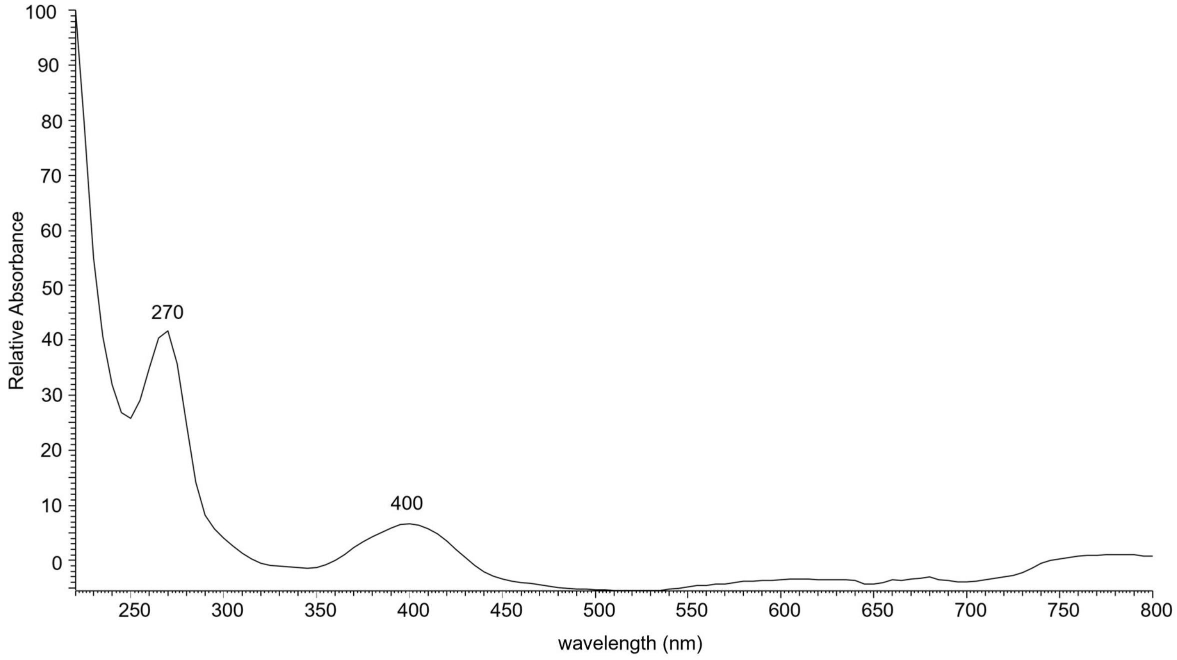

Figure S60. UV-Vis spectrum of trioxacarcin 958 (**15**).

HR-ESI-MS of trioxacarcin 958 (15)

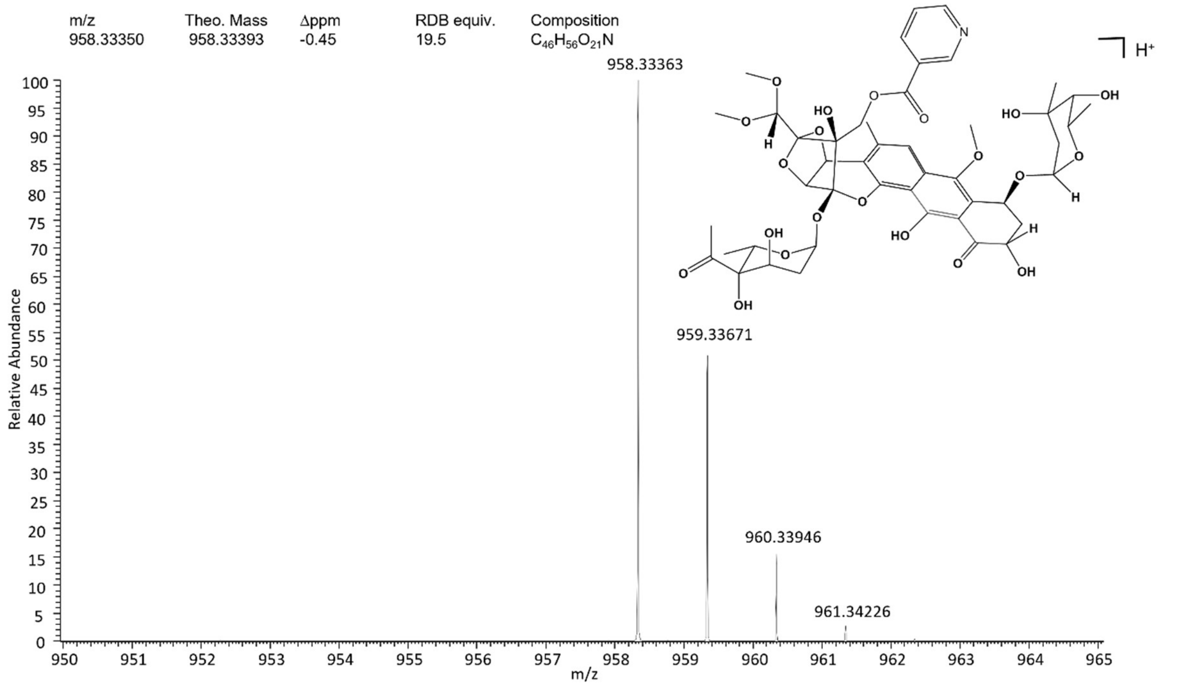

Figure S61. HR-ESI-MS of trioxacarcin 958 (**15**). Retention time 17.6 min, Macherey-Nagel RP8 column (250 × 2 mm, 5 µm), LC-MS program 1.

ESI-MS/MS of trioxacarcin 958 (15)

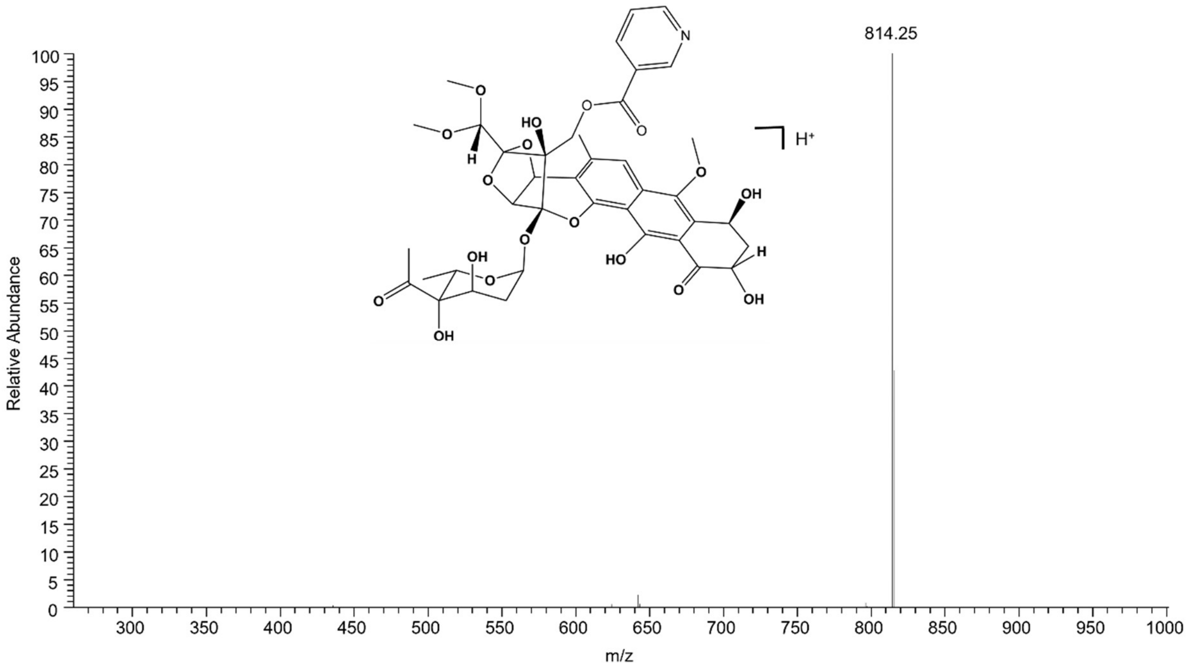

Figure S62. HR-ESI-MS/MS fragmentation of trioxacarcin 958 (**15**). MS/MS of the quasimolecular ion *m*/*z* 958 of **15**.

Trioxacarcin 1000 (16)

UV-Vis spectrum of trioxacarcin 1000 (16)

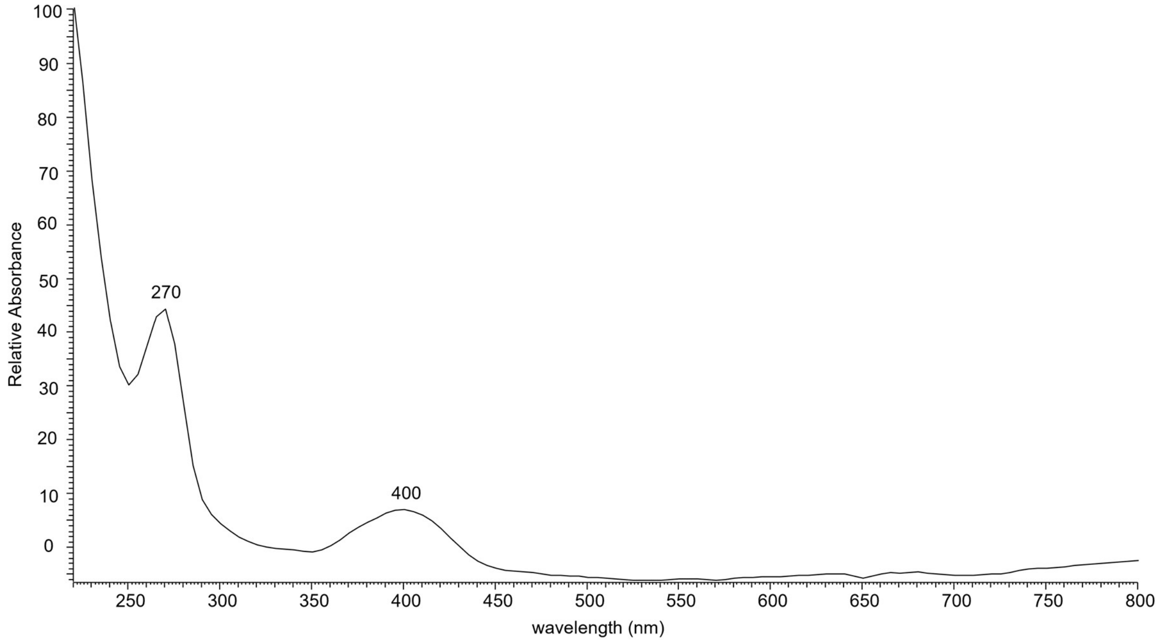

Figure S63. UV-Vis spectrum of trioxacarcin 1000 (**16**).

HR-ESI-MS of trioxacarcin 1000 (16)

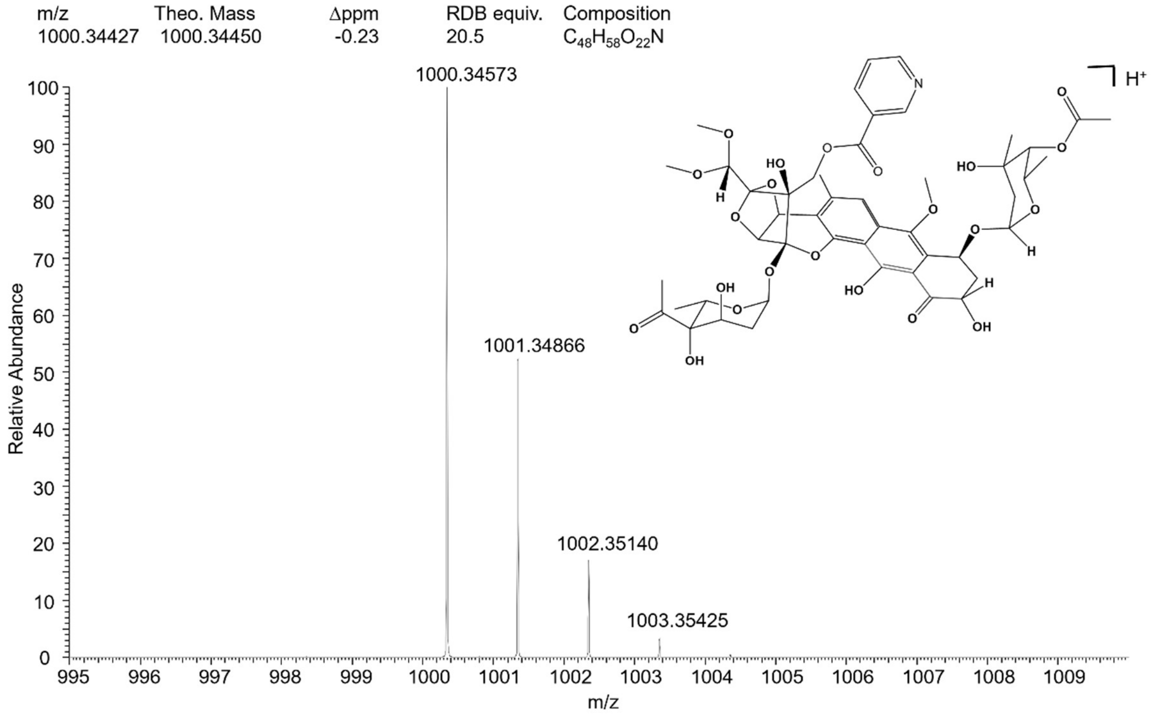

Figure S64. HR-ESI-MS of trioxacarcin 1000 (**16**). Retention time 27.8 min, Macherey-Nagel RP8 column (250 × 2 mm, 5 μm), LC-MS program 1.

ESI-MS/MS of trioxacarcin 1000 (16)

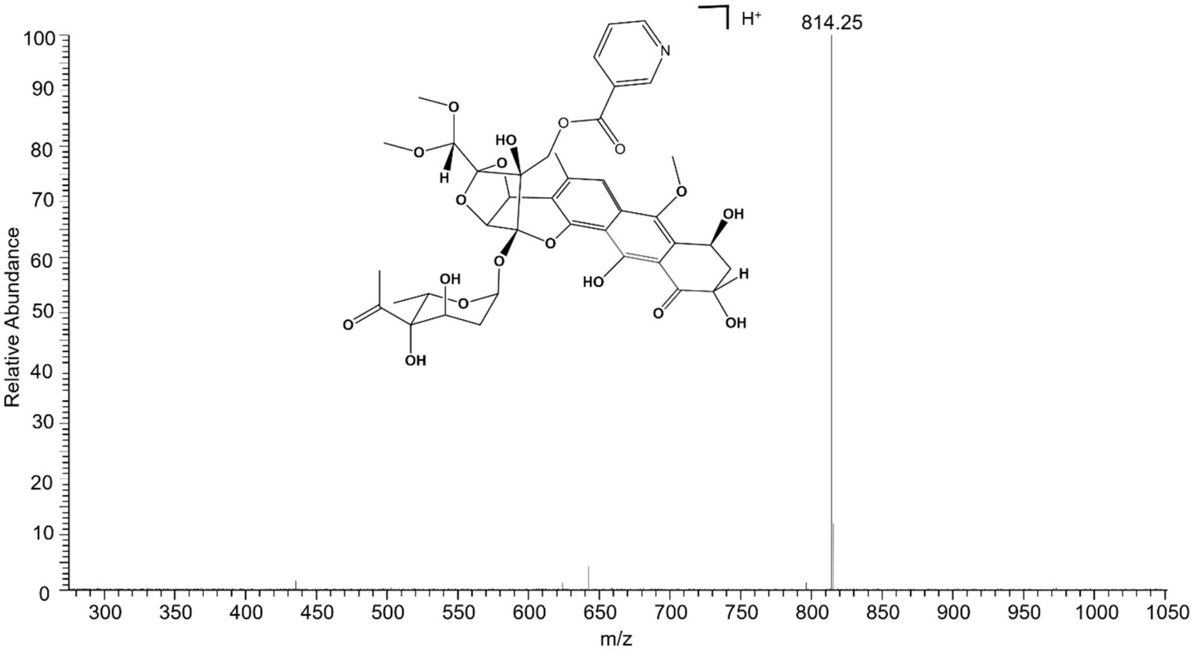

Figure S65. HR-ESI-MS/MS fragmentation of trioxacarcin 1000 (**16**). MS/MS of the quasimolecular ion *m*/*z* 1000 of **16**.

Production of trioxacarcin 780 (1) and trioxacarcin 692 (2) in different production media

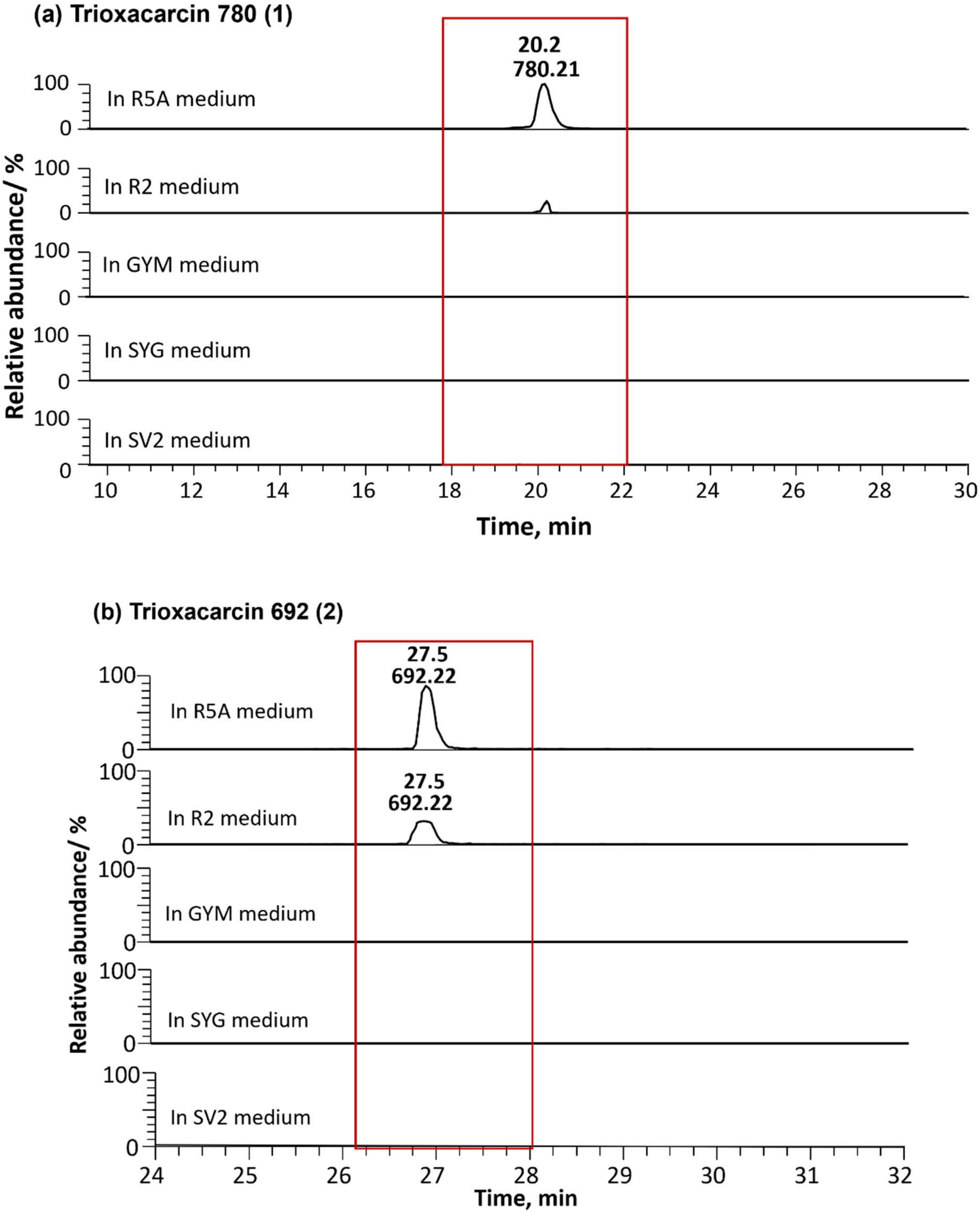

Figure S66. (**a**) Detection of trioxacarcin 780 (**1**), and (**b**) trioxacarcin 692 (**2**), in the processed supernatant of a 21-day old *Streptomyces* sp. 127Q culture. Strain 127Q was grown in R5A, R2 GYM, SYG, and SV2 media. LC-MS program 1, Macherey-Nagel RP8 column (250 × 2 mm, 5 μm).

Time course of trioxacarcins and gutingimycins formation

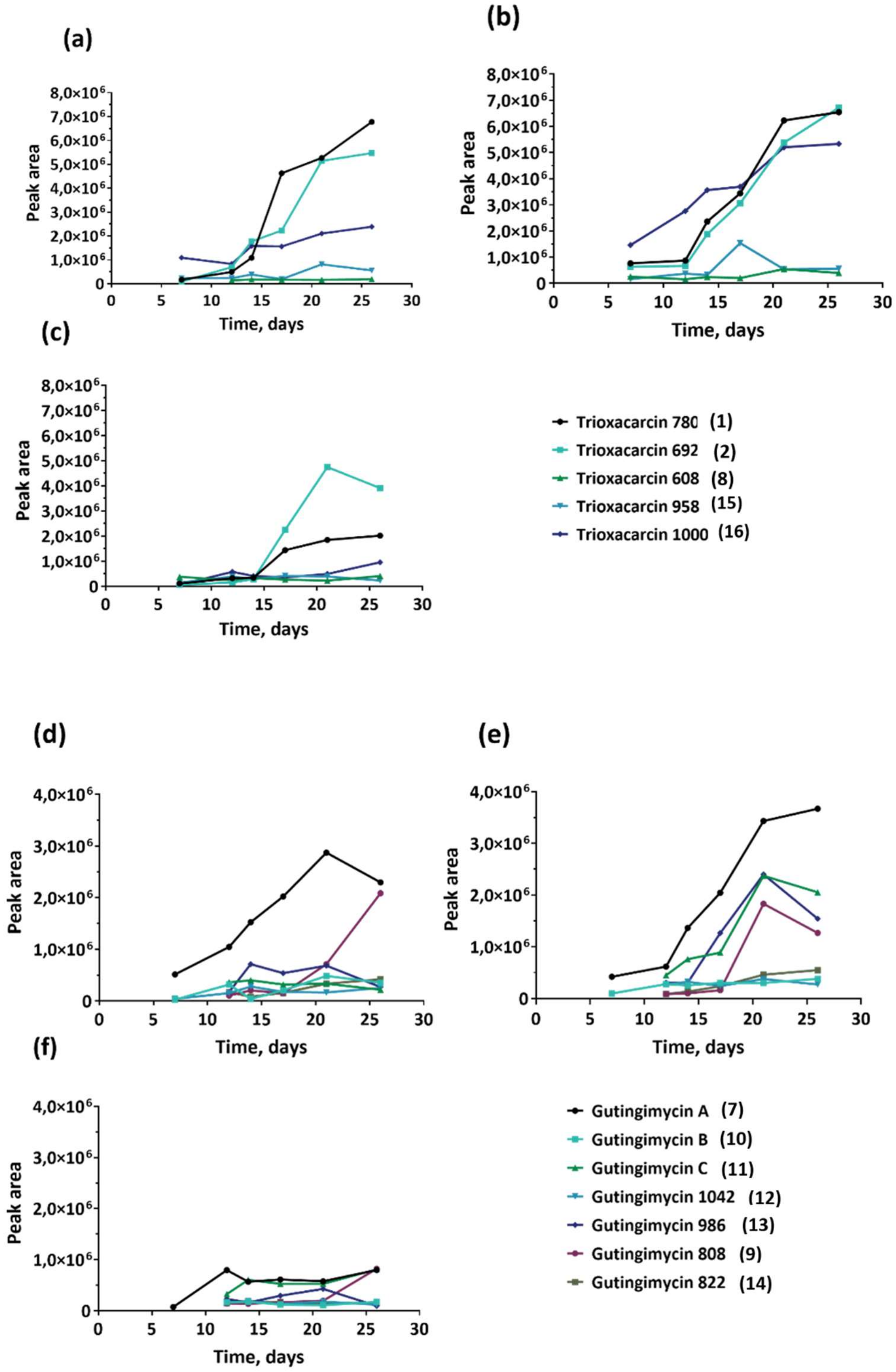

Figure S67. Time course of trioxacarcins and gutingimycins formation in R5A medium inoculated with precultures of different ages. The relative production of trioxacarcins (**a**-**c**) and gutingimycins (**d**-**f**) was monitored over a 26-day cultivation period by comparing the quasimolecular ion peak areas. Samples of 1.5 ml were collected every 3-5 d and extracted with ethyl acetate prior to LC-MS analysis. Panels (**a**) and (**d**) correspond to samples inoculated with 2-d-old pre-cultures, panels (**b**) and (**e**) to samples inoculated with to 3-d-old pre-cultures,

and panels (**c**) and (**f**) to samples inoculated with 4-d-old pre-cultures. The time course experiment reflects qualitative differences in the trioxacarcins-to-gutingimycins ratio at different time points, and the qualitative trend of trioxacarcins-to-gutingimycins formation. No internal standard was used due to the lack of a suitable standard. Moreover, ethyl acetate extracts were analysed, which gives a clean sample for LC-MS analysis compared to the very sugar-rich supernatant. This could cause that some of the trioxacarcins/gutingimycins are under- or overrepresented. Consequently, the graphs should be interpreted with caution. Clear differences in the product ratio can be observed between samples inoculated with precultures of different ages.

Trioxacarcin biosynthetic gene cluster in *Streptomyces* sp. 127Q

Table S4. Comparison of the genes in the trioxacarcin biosynthetic gene clusters of *Streptomyces* sp. 127Q (accession nr.: PZ363022) and *Streptomyces bottropensis* DO-45 (KP410250). The table lists genes identified in *Streptomyces* sp. 127Q, the predicted protein lengths (amino acids) in *Streptomyces* sp. 127Q versus *S. bottropensis* DO-45, BLAST percentage identity (% ID), and proposed functions. In either of the microorganisms, missing genes are highlighted in bold.

Gene	AA length	Protein homolog, accession no.	BLAST % ID	Proposed function
-	**-/94**	***txn*Rg1**, AKT74254	-	Regulator
GM001781	331/330	*Txn*B1, AKT74255	84	dTDP-glucose 4,6-dehydratase
GM001782	343/290	*Txn*B2, AKT74256	71	dTDP-glucose synthase
GM001783	316/327	*Txn*B3, AKT74257	79	Pyruvate dehydrogenase-α
GM001784	328/345	*Txn*B4, AKT74258	86	Pyruvate dehydrogenase-β
GM001785	**62/-**	-	-	DUF6059 family protein
GM001786	546/561	*Txn*A4, AKT74259	65	Methylacyltransferase (MAT)
GM001787	84/90	*Txn*A3, AKT74260	80	Acyl carrier protein ACP
GM001788	423/406	*Txn*A2, AKT74261	83	Chain length factor CLF (KS_{β})
GM001789	420/423	*Txn*A1, AKT74262	89	Ketosynthase (KS_{α})
GM001790	263/263	*Txn*Rg2, AKT74263	86	SARP-family regulator
GM001791	290/394	*Txn*Rg3, AKT74264	54	2-Component kinase
GM001792	203/203	*Txn*Rg4, AKT74265	88	2-Component regulator
-	**-/579**	***Txn*P1**, AKT74266	-	ATP-dependent CoA synthetase
GM001793	323/318	*Txn*C1, AKT74267	74	Aromatase
GM001794	344/344	*Txn*A5, AKT74268	86	Ketosynthase type III (KS-III)
GM001795	444/543	*Txn*P2, AKT74269	47	2-Isopropylmalate synthase
GM001796	411/417	*Txn*P3, AKT74270	74	Dehydratase or isomerase
GM001797	260/260	*Txn*P4, AKT74271	82	Short-chain dehydrogenase
GM001798	500/500	*Txn*Rr1, AKT74272	62	Transporter
GM001799	344/345	*Txn*O1, AKT74273	81	Dehydrogenase
GM001800,	126/126	*Txn*U1, AKT74274	75	Unknown function
GM001801	405/401	*Txn*O2, AKT74275	86	Cytochrome P450
GM001802	370/366	*Txn*U2, AKT74276	74	Unknown function
**-**	**-/411**	***Txn*O3**, AKT74277	-	Ferredoxin reductase
GM001803	107/107	*Txn*O4, AKT74278	89	Ferredoxin
GM001804	494/494	*Txn*H1, AKT74279	80	Hydrolase
GM001805	408/409	*Txn*O5, AKT74280	89	Cytochrome P450
GM001806	373/373	*Txn*H2, AKT74281	85	Epoxide hydrolase
GM001807	325/328	*Txn*B5, AKT74282	69	dNDP-hexose-4-ketoreductase
GM001808	215/213	*Txn*B6, AKT74283	74	3,5-Epimerase
GM001809	413/413	*Txn*M1, AKT74284	86	dNDP-hexose 3-C-Methyltrans-ferase
GM001810	480/488	*Txn*B7, AKT74285	76	dNDP-hexose-2,3-dehydratase
GM001811	321/321	*Txn*B8, AKT74286	81	Transporter

Gene	AA length	Protein homolog, accession no.	BLAST % ID	Proposed function
GM001812	480/500	*Txn*Rr2, AKT74287	80	Regulator
GM001813	330/339	*Txn*Rg5, AKT74288	84	Regulator
GM001814	406/406	*Txn*O6, AKT74289	89	Cytochrome P450
GM001815	176/175	*Txn*O7, AKT74290	88	Cyclase or hydroxylase
GM001816	371/371	*Txn*O8, AKT74291	80	Hydroxylase
GM001817	154/154	*Txn*O9, AKT74292	89	Cyclase or hydroxylase
GM001818	261/261	*Txn*C2, AKT74293	86	Ketoreductase
GM001819	178/178	*Txn*O10, AKT74294	78	Flavin reductase
GM001820	306/304	*Txn*C3, AKT74295	79	2,3-Cyclase
-	**-/383**	***Txn*B9**, AKT74296	-	Glucosyltransferase
GM001821	174/148	*Txn*O11, AKT74297	75	Cyclase or hydroxylase
GM001822	122/121	*Txn*U3, AKT74298	76	Unknown function
GM001823	425/424	*Txn*B10, AKT74299	77	Glycosyltransferase
GM001824	241/240	*Txn*C4, AKT74302	86	Ketoreductase
GM001825	340/340	*Txn*M2, AKT74301	83	*O*-Methyltransferase
GM001826	372/388	*Txn*U4, AKT74302	80	Unknown function
GM001827	394/397	*Txn*B11, AKT74303	73	*O*-Acyltransferase
GM001828	427/427	*Txn*B12, AKT74304	82	Glycosyltransferase
GM001829	340/339	*Txn*M3, AKT74305	88	*O*-Methyltransferase
GM001830	408/407	*Txn*O12, AKT74306	89	Cytochrome P450
GM001831	340/340	*Txn*M4, AKT74307	86	*O*-Methyltransferase
GM001832	176/182	*Txn*U5, AKT74308	75	Unknown function
	**-/286**	***Txn*Rg6**, AKT74309	-	Regulator

Comparison of trioxacarcin biosynthetic gene clusters

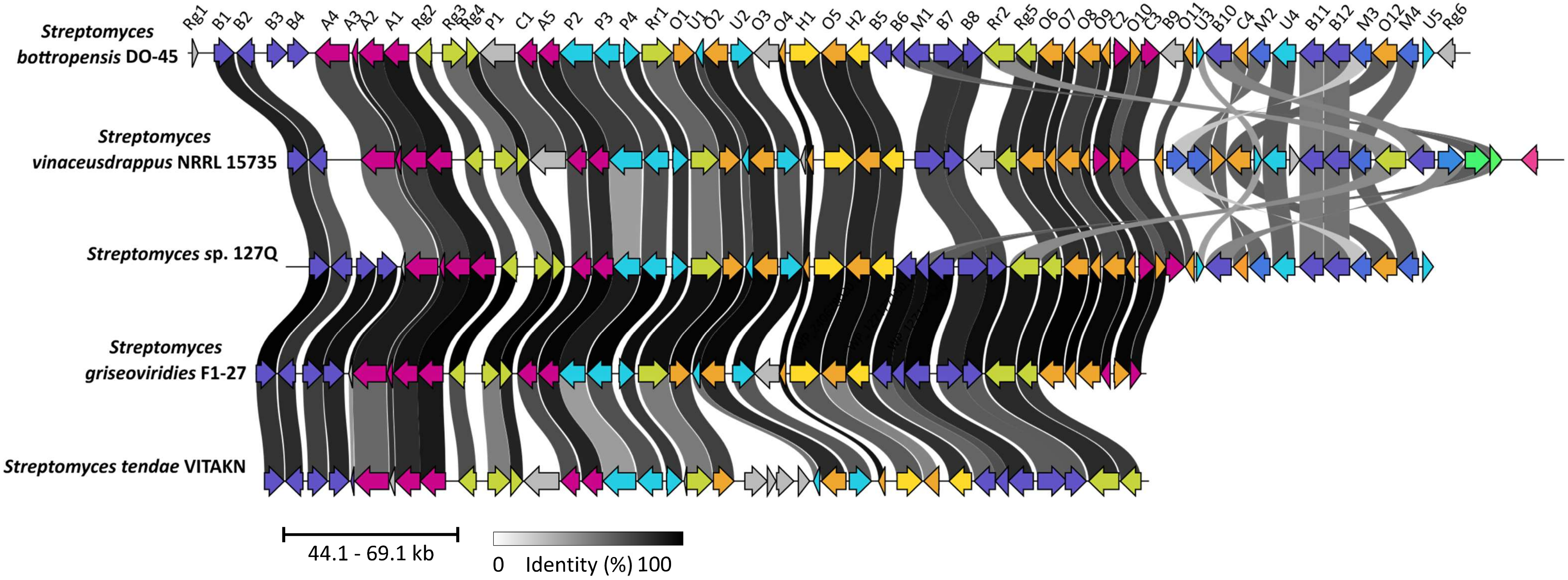

Figure S68. Alignment of the trioxacarcin biosynthetic gene clusters from three trioxacarcin producers and two putative trioxacarcin producers. The alignment was generated using Clinker v0.0.28: *Streptomyces bottropensis* DO-45 (accession nr.: KP410250), *Streptomyces vinaceusdrappus* NRPL 15735 (accession nr.: CP104697), *Streptomyces* sp. 127Q (accession nr.: PZ363022), *Streptomyces griseoviridies* D1-27 (accession nr.: CP034687), and *Streptomyces tendae* VITAKN (accession nr.: JAAIFS000000000). Color coding indicates genes for the polyketide synthases and its associated enzymes (pink), oxidation-reduction enzymes (orange), deoxysugar biosynthesis enzymes (purple), epoxide hydrolases (yellow), regulators and resistance mechanism proteins (green), DNA-alkylating enzymes and unknown-function enzymes (blue). Genes present only in a subset of the gene clusters are highlighted in grey.

Preliminary screening of the antimicrobial activity of trioxacarcin 780 (1) against human and bee pathogens

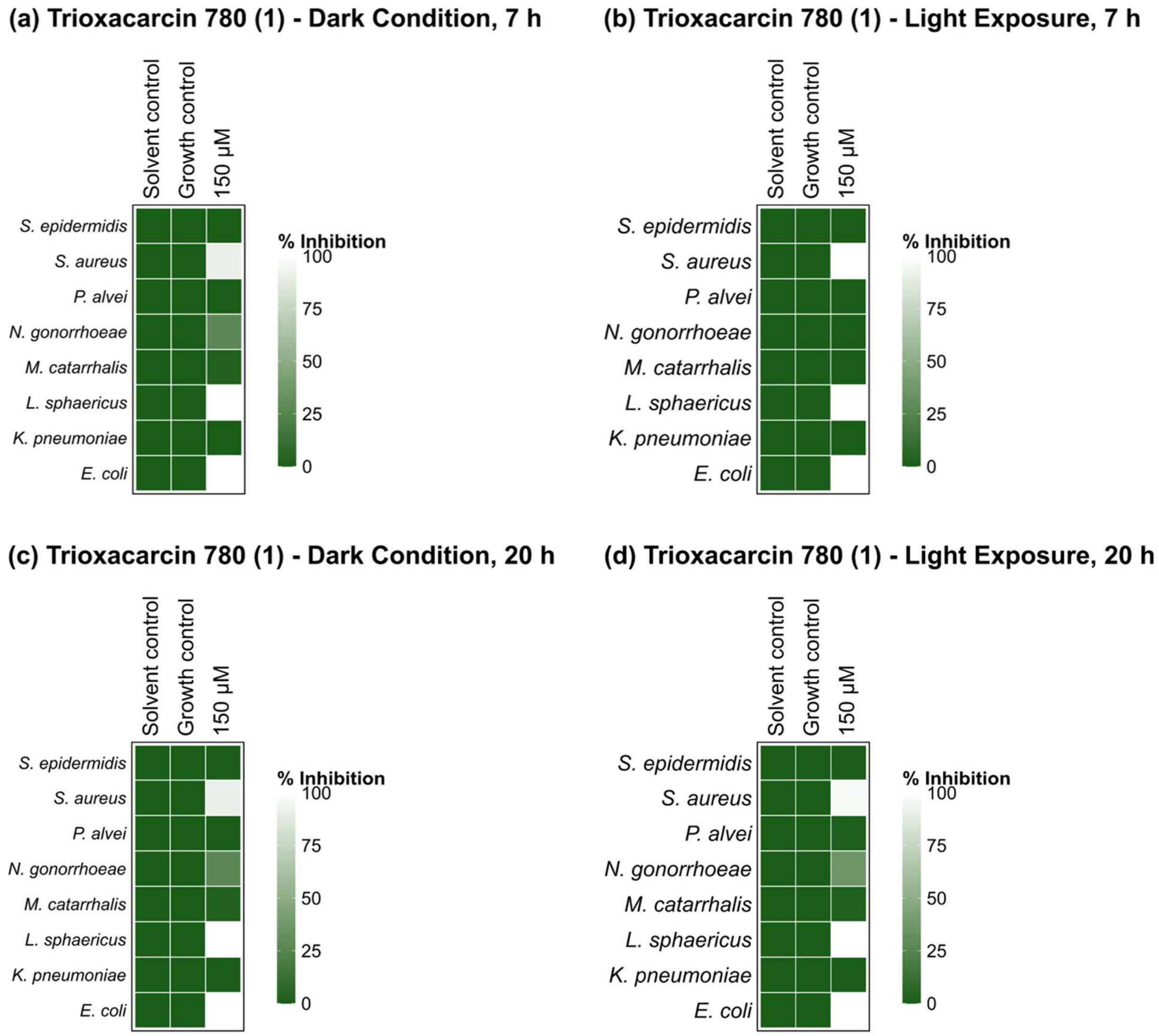

Figure S69. Preliminary screening of the antibacterial activity of trioxacarcin 780 (**1**) against the human pathogens *E. coli*, *S. aureus*, *N. gonorrhoeae*, *M. catarrhalis*, *K. pneumoniae*, and *S. epidermidis*, as well as the bee pathogens *L. sphaericus* and *P. alvei*. Bacterial growth was measured after 7 h and 20 h in a 96-well plate. The antibiotic activity of **1** was determined under dark condition [(**a**), (**c**)] after 7h and 20 h, respectively; and after light exposure [(**b**), (**d**)] after 7 h and 20 h, respectively. For light exposure, a combined red/blue LED lamp (λ = 465 nm + 620 nm, 60 min) was used. Percentage growth inhibition was calculated relative to the solvent control (1% DMSO). Growth control corresponds to microorganisms growing in their respective media without the addition of DMSO or the test compound. The heatmap color scale represents the percentage of inhibition, ranging from 0 % (dark green, no inhibition) to 100% (white, complete inhibition). The results are based on a single replicate (n = 1).

Preliminary screening of the antimicrobial activity of trioxacarcin 692 (2) against human and bee pathogens

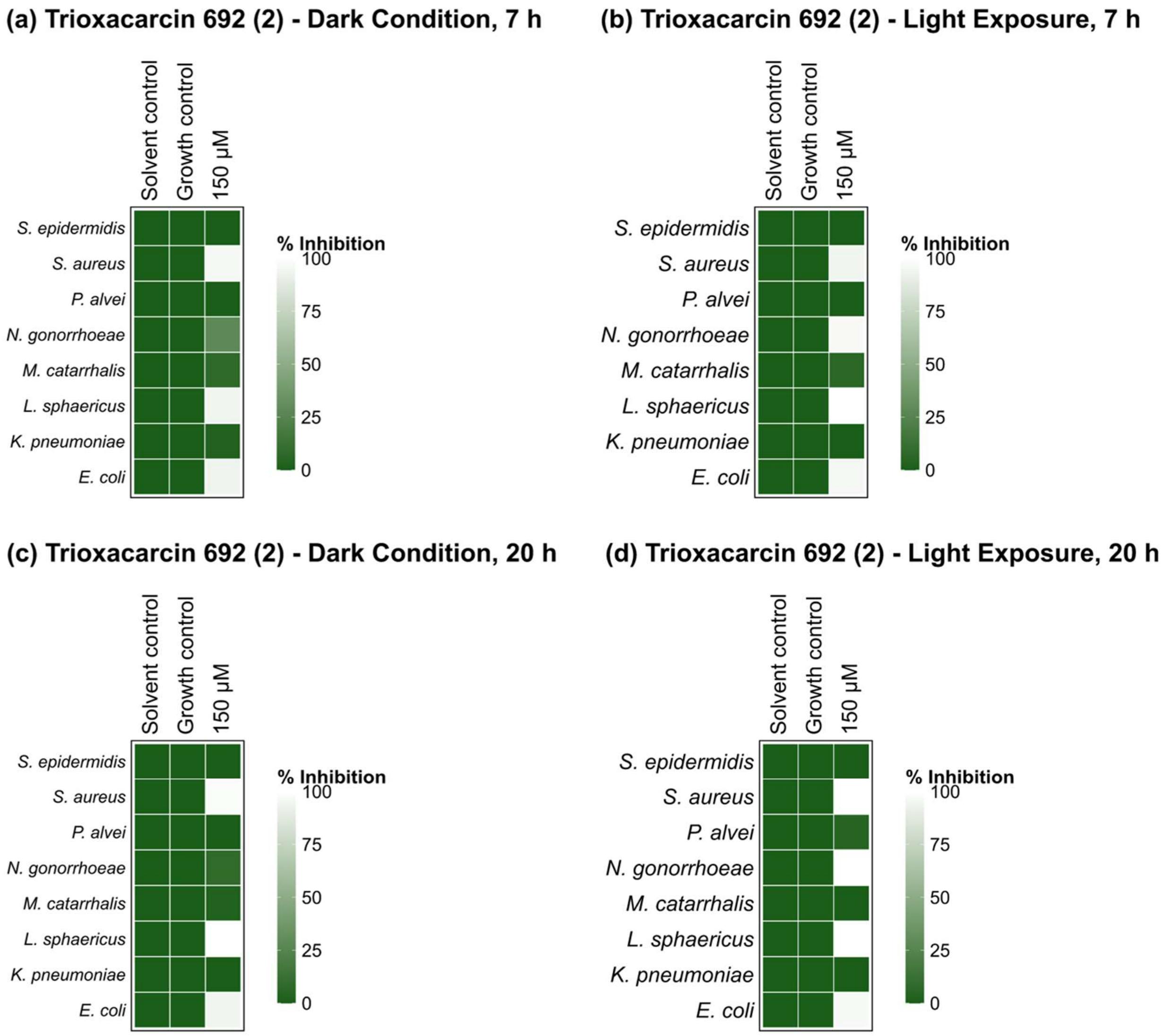

Figure S70. Preliminary screening of the antibacterial activity of trioxacarcin 692 (**2**) against the human pathogens *E. coli*, *S. aureus*, *N. gonorrhoeae*, *M. catarrhalis*, *K. pneumoniae*, and *S. epidermidis*, as well as the bee pathogens *L. sphaericus* and *P. alvei*. Bacterial growth was measured after 7 h and 20 h in a 96-well plate. The antibiotic activity of **2** was determined under dark condition [(**a**), (**c**)] after 7h and 20 h, respectively; and after light exposure [(**b**), (**d**)] after 7 h and 20 h, respectively. For light exposure, a combined red/blue LED lamp (λ = 465 nm + 620 nm, 60 min) was used. Percentage growth inhibition was calculated relative to the solvent control (1% DMSO). Growth control corresponds to microorganisms growing in their respective media without the addition of DMSO or the test compound. The heatmap color scale represents the percentage of inhibition, ranging from 0 % (dark green, no inhibition) to 100% (white, complete inhibition). The results are based on a single replicate (n = 1).

Antibacterial activity of trioxacarcin 780 (1) against *Escherichia coli*

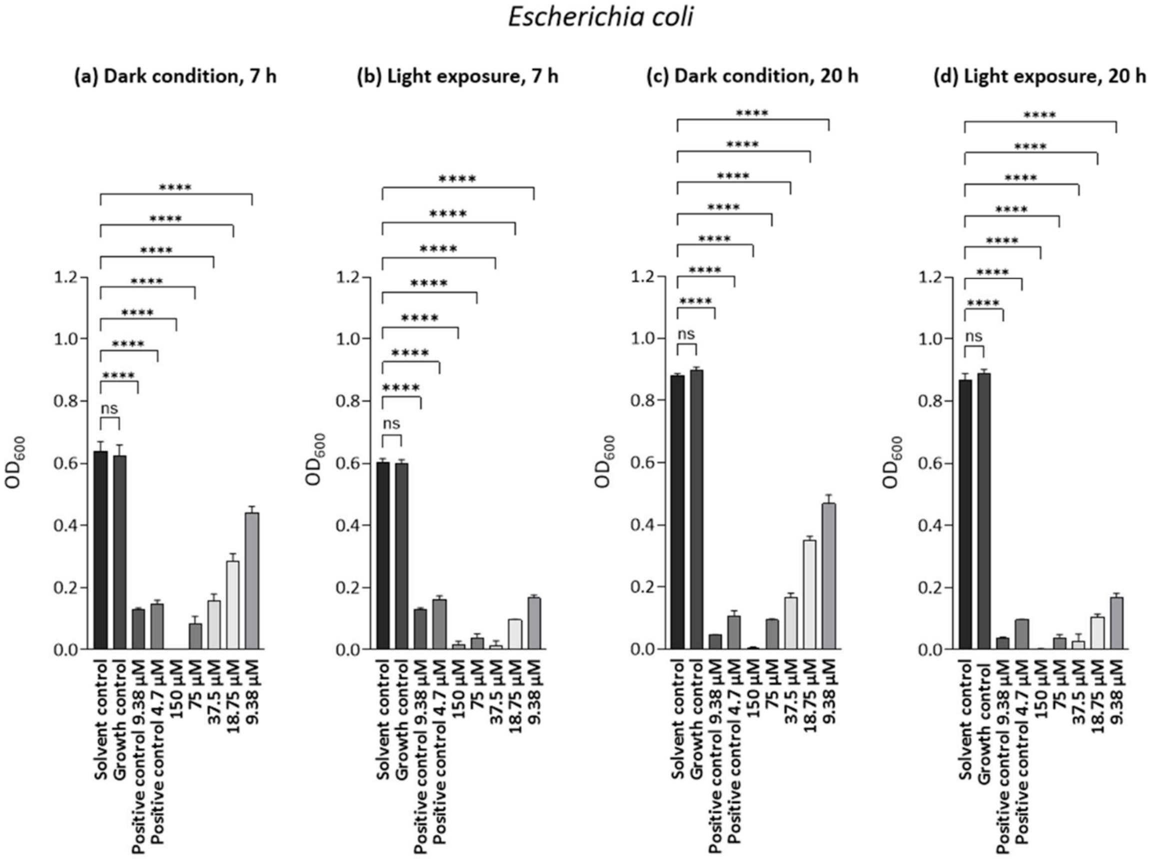

Figure S71. Antibacterial activity of trioxacarcin 780 (**1**) against *E. coli*. (**a**) Dark condition, 7 h; (**b**) Light exposure, 7 h; (**c**) Dark condition, 20 h; (**d**) Light exposure, 20 h. For light exposure a combined red/blue LED lamp (λ = 465 nm + 620 nm, 60 min) was used.

Solvent control: microorganism grown in respective medium with addition of DMSO (1% final concentration).

Growth control: microorganism grown in respective medium without addition of DMSO and without test compound.

Positive control: microorganism grown in respective medium with addition of ciprofloxacin (4.7 µM, 9.4 µM) in DMSO (1% final concentration).

Baseline value: medium and trioxacarcin 780 (**1**) in DMSO (1% final concentration) was incubated alongside in the 96 well plate and served for background subtraction.

In the light exposure condition, solvent controls, growth controls, positive controls, baseline values and test samples were equally subjected to irradiation.

Growth (OD_{600}) was measured after 7 h in a 96-well plate. Bars represent mean ± standard deviation (SD) of three independent replicates inoculated from the same preculture (n = 3). Statistical significance was determined by one-way ANOVA with Dunnett's post hoc test compared to 1% DMSO control. NS, not significant; $P < 0.05$ (*), $P < 0.01$ (**), $P < 0.001$ (***), $P < 0.0001$ (****).

The lowest concentration tested (9.4 µM) did not result in complete loss of the activity. Therefore, the MIC value is below the tested concentration range.

Antibacterial activity of trioxacarcin 780 (1) against *Staphylococcus aureus*

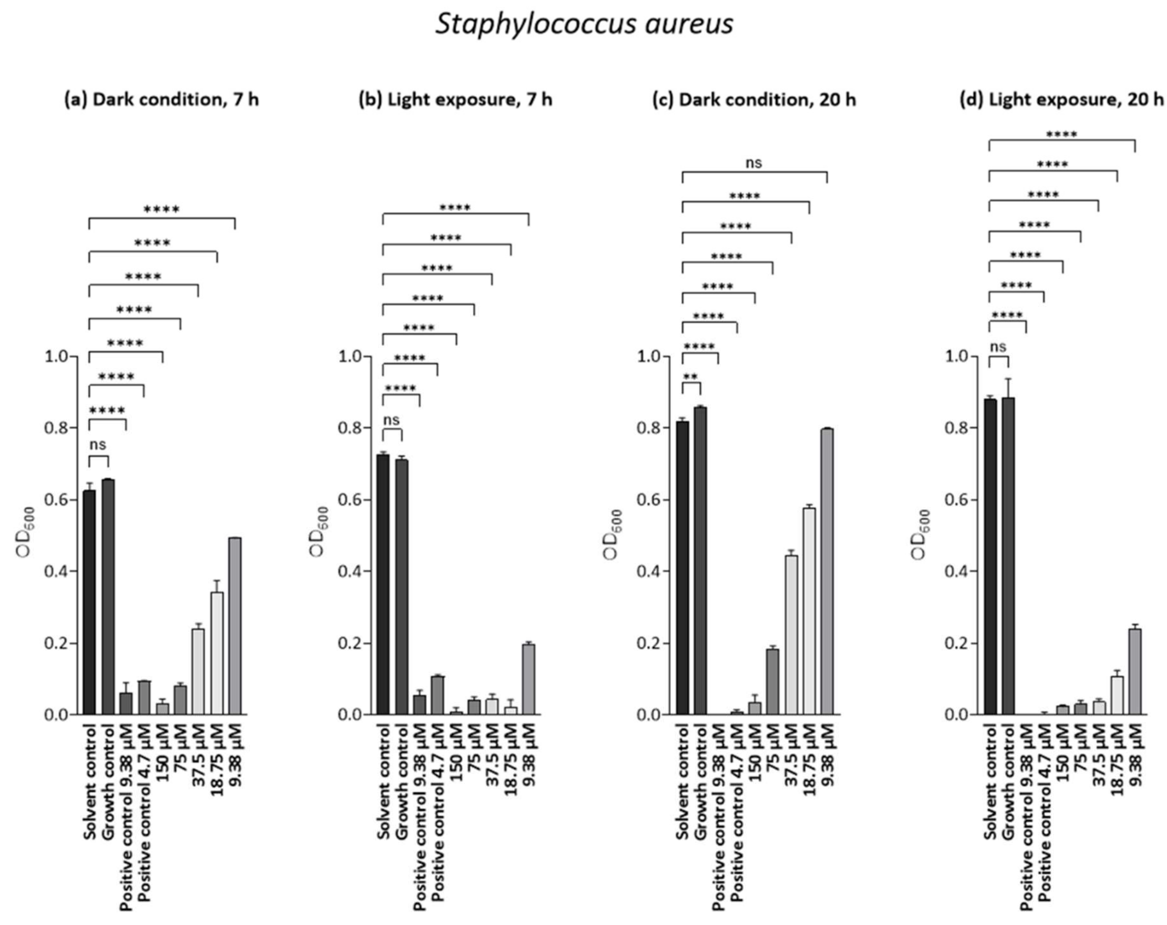

Figure S72. Antibacterial activity of trioxacarcin 780 (**1**) against *S. aureus*. (**a**) Dark condition, 7 h; (**b**) Light exposure, 7 h; (**c**) Dark condition, 20 h; (**d**) Light exposure, 20 h. For light exposure a combined red/blue LED lamp (λ = 465 nm + 620 nm, 60 min) was used.

Solvent control: microorganism grown in respective medium with addition of DMSO (1% final concentration).

Growth control: microorganism grown in respective medium without addition of DMSO and without test compound.

Positive control: microorganism grown in respective medium with addition of vancomycin (4.7 µM, 9.4 µM) in DMSO (1% final concentration).

Baseline value: medium and trioxacarcin 780 (**1**) in DMSO (1% final concentration) was incubated alongside in the 96 well plate and served for background subtraction.

In the light exposure condition, solvent controls, growth controls, positive controls, baseline values and test samples were equally subjected to irradiation.

Growth (OD_{600}) was measured after 7 h in a 96-well plate. Bars represent mean ± standard deviation (SD) of three independent replicates inoculated from the same preculture (n = 3). Statistical significance was determined by one-way ANOVA with Dunnett's post hoc test compared to 1% DMSO control. NS, not significant; $P < 0.05$ (*), $P < 0.01$ (**), $P < 0.001$ (***), $P < 0.0001$ (****).

The lowest concentration tested (9.4 µM) did not result in complete loss of the activity. Therefore, the MIC value is slightly below the tested concentration range.

Antibacterial activity of trioxacarcin 780 (1) against *Neisseria gonorrhoeae*

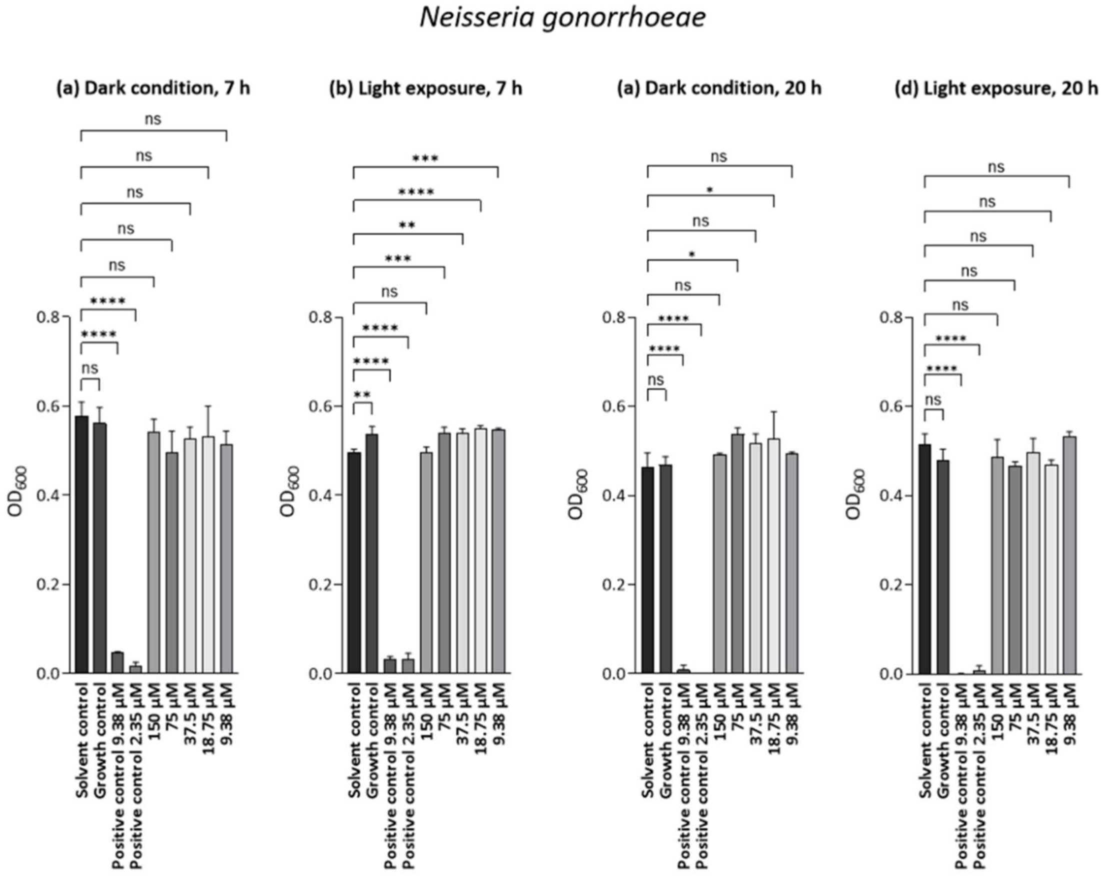

Figure S73. Antibacterial activity of trioxacarcin 780 (**1**) against *N. gonorrhoeae*. (**a**) Dark condition, 7 h; (**b**) Light exposure, 7 h; (**c**) Dark condition, 20 h; (**d**) Light exposure, 20 h. For light exposure a combined red/blue LED lamp (λ = 465 nm + 620 nm, 60 min) was used.

Solvent control: microorganism grown in respective medium with addition of DMSO (1% final concentration).

Growth control: microorganism grown in respective medium without addition of DMSO and without test compound.

Positive control: microorganism grown in respective medium with addition of ciprofloxacin (9.4 µM, 2.4 µM) in DMSO (1% final concentration).

Baseline value: medium and trioxacarcin 780 (**1**) in DMSO (1% final concentration) was incubated alongside in the 96 well plate and served for background subtraction.

In the light exposure condition, solvent controls, growth controls, positive controls, baseline values and test samples were equally subjected to irradiation.

Growth (OD_{600}) was measured after 7 h in a 96-well plate. Bars represent mean ± standard deviation (SD) of three independent replicates inoculated from the same preculture (n = 3). Statistical significance was determined by one-way ANOVA with Dunnett's post hoc test compared to 1% DMSO control. NS, not significant; $P < 0.05$ (*), $P < 0.01$ (**), $P < 0.001$ (***), $P < 0.0001$ (****).

Antibacterial activity of trioxacarcin 780 (1) against *Lysinibacillus sphaericus*

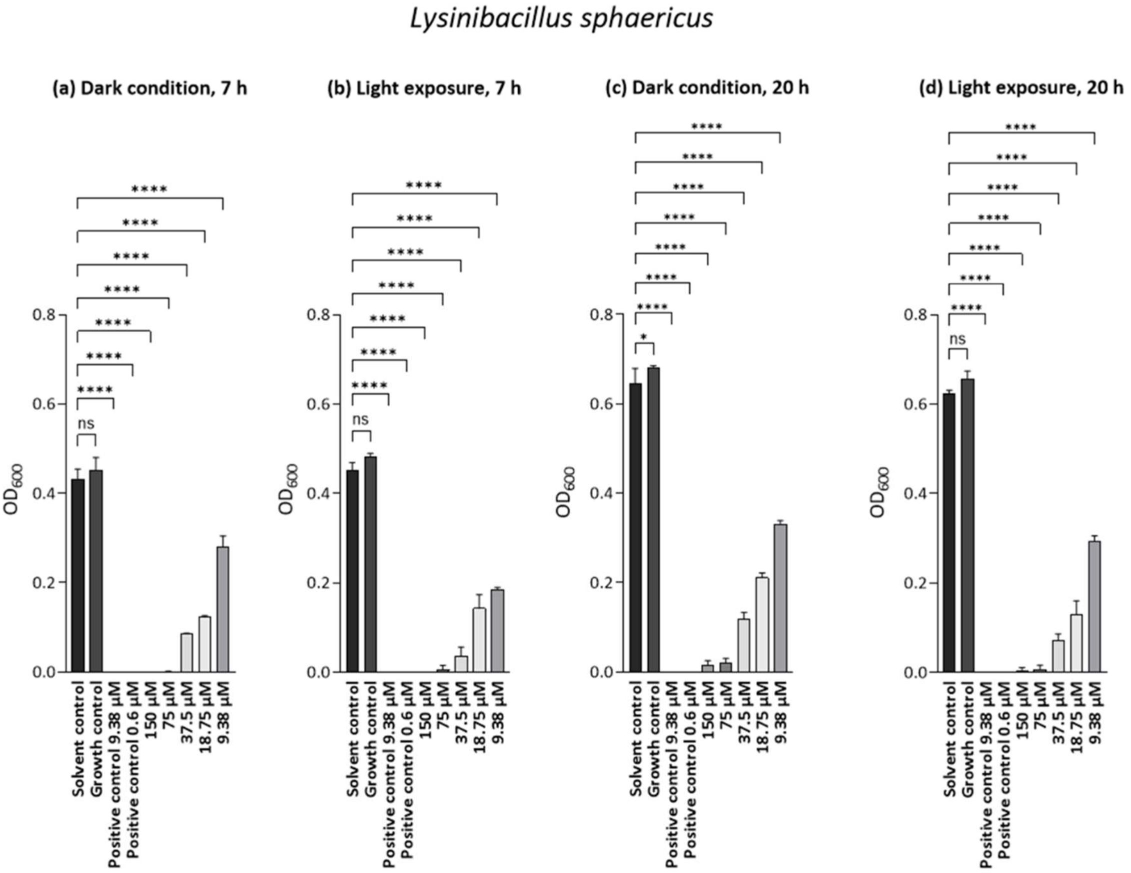

Figure S74. Antibacterial activity of trioxacarcin 780 (**1**) against *L. sphaericus*. (**a**) Dark condition, 7 h; (**b**) Light exposure, 7 h; (**c**) Dark condition, 20 h; (**d**) Light exposure, 20 h. For light exposure a combined red/blue LED lamp (*λ* = 465 nm + 620 nm, 60 min) was used.

Solvent control: microorganism grown in respective medium with addition of DMSO (1% final concentration).

Growth control: microorganism grown in respective medium without addition of DMSO and without test compound.

Positive control: microorganism grown in respective medium with addition of vancomycin (9.4 µM, 0.6 µM) in DMSO (1% final concentration).

Baseline value: medium and trioxacarcin 780 (**1**) in DMSO (1% final concentration) was incubated alongside in the 96 well plate and served for background subtraction.

In the light exposure condition, solvent controls, growth controls, positive controls, baseline values and test samples were equally subjected to irradiation.

Growth (OD_{600}) was measured after 7 h in a 96-well plate. Bars represent mean ± standard deviation (SD) of three independent replicates inoculated from the same preculture (n = 3). Statistical significance was determined by one-way ANOVA with Dunnett's post hoc test compared to 1% DMSO control. NS, not significant; $P < 0.05$ (*), $P < 0.01$ (**), $P < 0.001$ (***), $P < 0.0001$ (****). The lowest concentration tested (9.4 µM) did not result in complete loss of the activity. Therefore, the MIC value is slightly below the tested concentration range.

Antibacterial activity of trioxacarcin 692 (2) against *Escherichia coli*

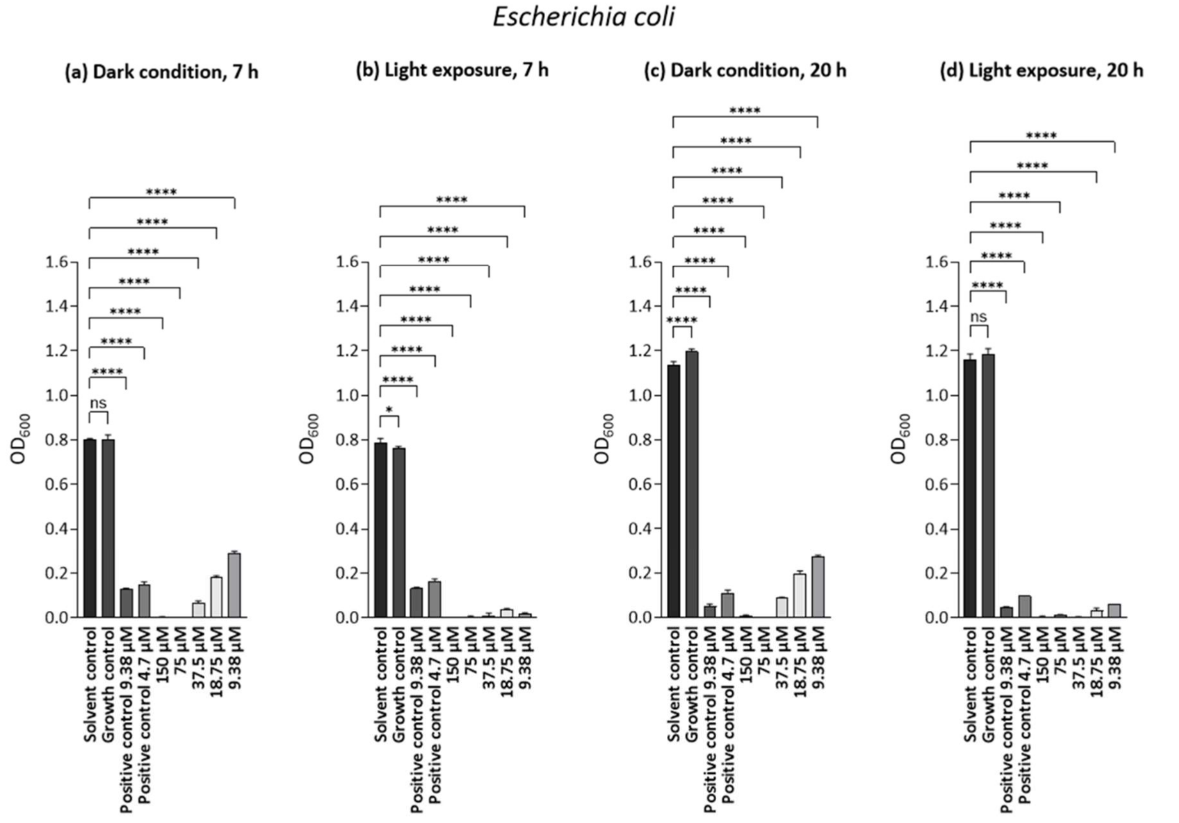

Figure S75. Antibacterial activity of trioxacarcin 692 (**2**) against *E. coli*. (**a**) Dark condition, 7h; (**b**) Light exposure, 7 h; (**c**) Dark condition, 20 h; (**d**) Light exposure, 20 h. For light exposure a combined red/blue LED lamp (λ = 465 nm + 620 nm, 60 min) was used.

Solvent control: microorganism grown in respective medium with addition of DMSO (1% final concentration).

Growth control: microorganism grown in respective medium without addition of DMSO and without test compound.

Positive control: microorganism grown in respective medium with addition of ciprofloxacin (9.4 µM, 4.7 µM) in DMSO (1% final concentration).

Baseline value: medium and trioxacarcin 692 (**2**) in DMSO (1% final concentration) was incubated alongside in the 96 well plate and served for background subtraction.

In the light exposure condition, solvent controls, growth controls, positive controls, baseline values and test samples were equally subjected to irradiation.

Growth (OD_{600}) was measured after 7 h and 20 h in a 96-well plate. Bars represent mean ± standard deviation (SD) of three independent replicates inoculated from the same preculture (n = 3). Statistical significance was determined by one-way ANOVA with Dunnett's post hoc test compared to 1% DMSO control. NS, not significant; P < 0.05 (*), P < 0.01 (**), P < 0.001 (***), P < 0.0001 (****). The lowest concentration tested (9.4 µM) did not result in complete loss of the activity. Therefore, the MIC value is below the tested concentration range.

Antibacterial activity of trioxacarcin 692 (2) against *Staphylococcus aureus*

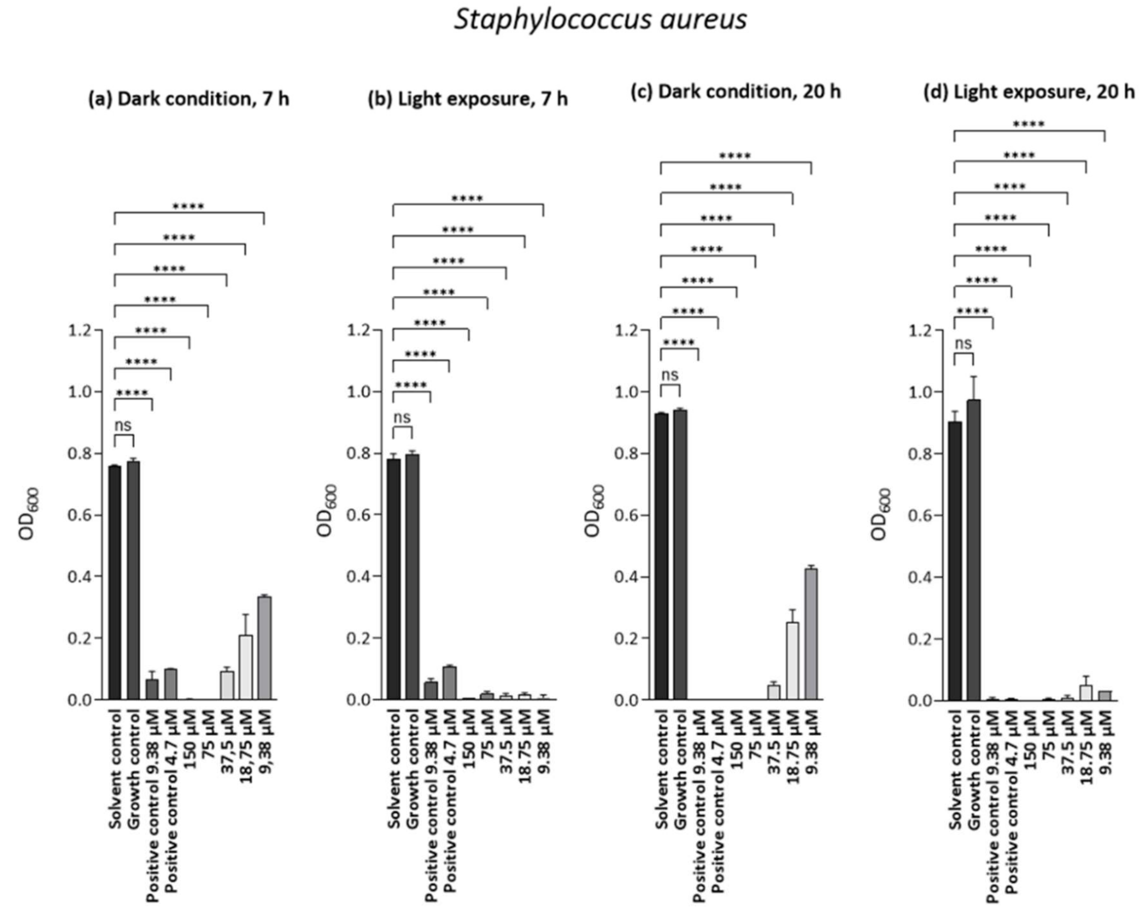

Figure S76. Antibacterial activity of trioxacarcin 692 (**2**) against *S. aureus*. (**a**) Dark condition, 7 h; (**b**) Light exposure, 7 h; (**c**) Dark condition, 20 h; (**d**) Light exposure, 20 h. For light exposure a combined red/blue LED lamp (λ = 465 nm + 620 nm, 60 min) was used.

Solvent control: microorganism grown in respective medium with addition of DMSO (1% final concentration).

Growth control: microorganism grown in respective medium without addition of DMSO and without test compound.

Positive control: microorganism grown in respective medium with addition of vancomycin (9.4 µM, 4.7 µM) in DMSO (1% final concentration).

Baseline value: medium and trioxacarcin 692 (**2**) in DMSO (1% final concentration) was incubated alongside in the 96 well plate and served for background subtraction.

In the light exposure condition, solvent controls, growth controls, positive controls, baseline values and test samples were equally subjected to irradiation.

Growth (OD_{600}) was measured after 7 h in a 96-well plate. Bars represent mean ± standard deviation (SD) of three independent replicates inoculated from the same preculture (n = 3). Statistical significance was determined by one-way ANOVA with Dunnett's post hoc test compared to 1% DMSO control. NS, not significant; $P < 0.05$ (*), $P < 0.01$ (**), $P < 0.001$ (***), $P < 0.0001$ (****). The lowest concentration tested (9.4 µM) did not result in complete loss of activity. Therefore, the MIC value is below the tested concentration range.

Antibacterial activity of trioxacarcin 692 (2) against *Neisseria gonorrhoeae*

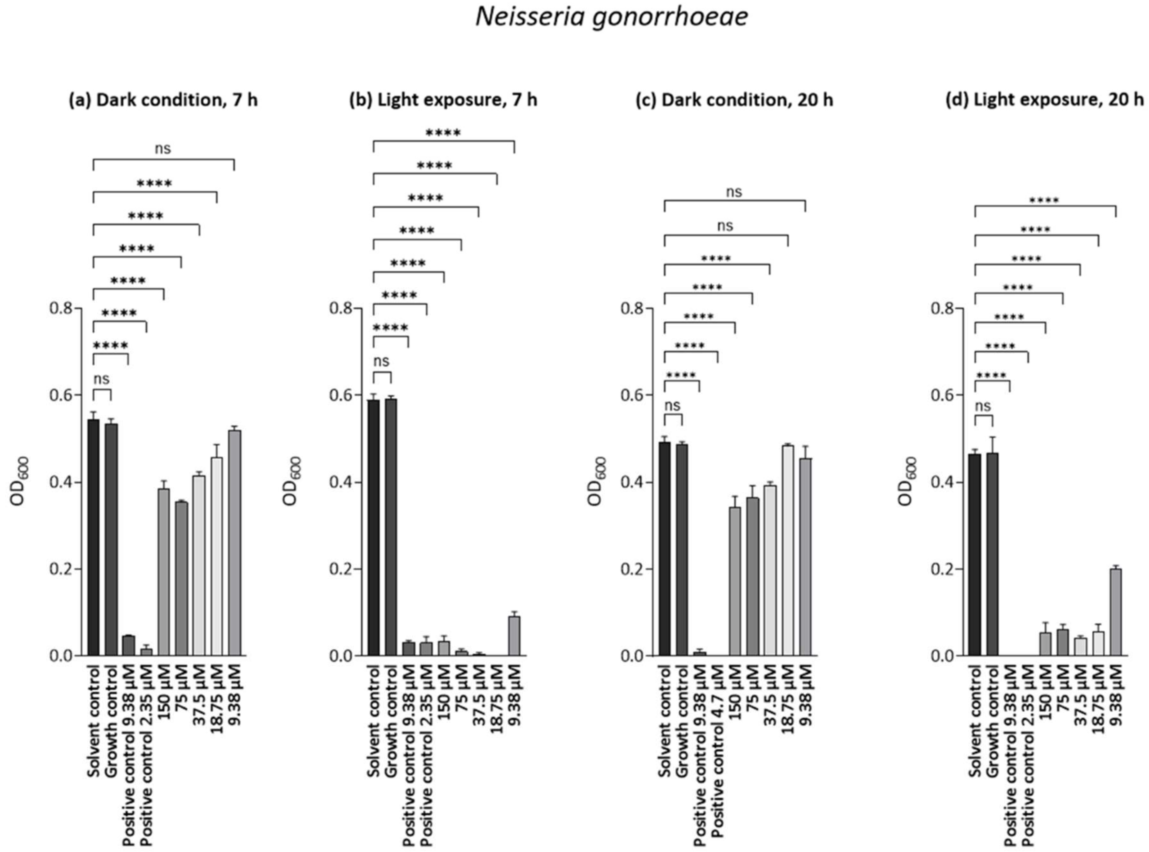

Figure S77. Antibacterial activity of trioxacarcin 692 (**2**) against *N. gonorrhoeae*. (**a**) Dark condition, 7 h; (**b**) Light exposure, 7 h; (**c**) Dark condition, 20 h; (**d**) Light exposure, 20 h. For light exposure a combined red/blue LED lamp (λ = 465 nm + 620 nm, 60 min) was used.

Solvent control: microorganism grown in respective medium with addition of DMSO (1% final concentration).

Growth control: microorganism grown in respective medium without addition of DMSO and without test compound.

Positive control: microorganism grown in respective medium with addition of ciprofloxacin (9.4 µM, 4.7 µM) in DMSO (1% final concentration).

Baseline value: medium and trioxacarcin 692 (**2**) in DMSO (1% final concentration) was incubated alongside in the 96 well plate and served for background subtraction.

In the light exposure condition, solvent controls, growth controls, positive controls, baseline values and test samples were equally subjected to irradiation.

Growth (OD_{600}) was measured after 7 h in a 96-well plate. Bars represent mean ± standard deviation (SD) of three independent replicates inoculated from the same preculture (n = 3). Statistical significance was determined by one-way ANOVA with Dunnett's post hoc test compared to 1% DMSO control. NS, not significant; $P < 0.05$ (*), $P < 0.01$ (**), $P < 0.001$ (***), $P < 0.0001$ (****). The lowest concentration tested (9.4 µM) did not result in complete loss of the activity. Therefore, the MIC value is slightly below the tested concentration range.

Antibacterial activity of trioxacarcin 692 (2) against *Lysinibacillus sphaericus*

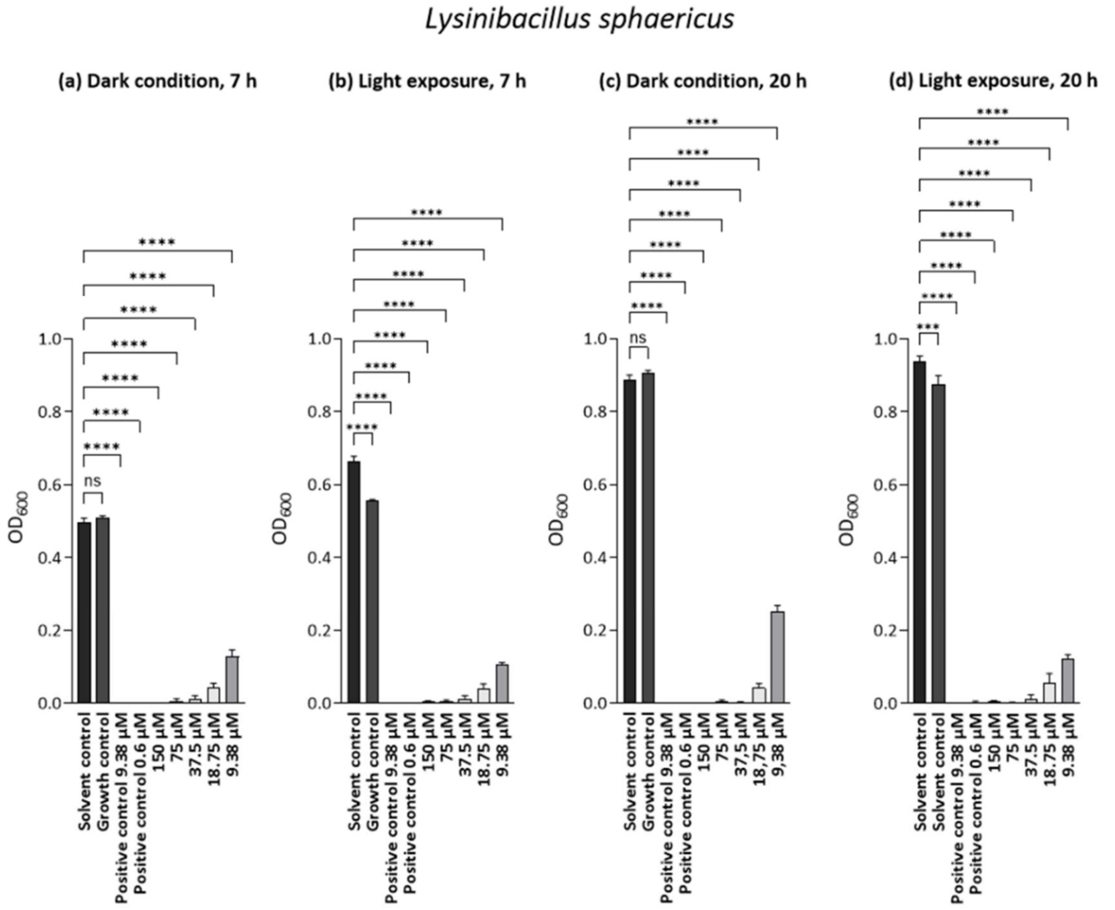

Figure S78. Antibacterial activity of trioxacarcin 692 (**2**) against *L. sphaericus*. (**a**) Dark condition, 7 h; (**b**) Light exposure, 7 h; (**c**) Dark condition, 20 h; (**d**) Light exposure, 20 h. For light exposure a combined red/blue LED lamp (λ = 465 nm + 620 nm, 60 min) was used.

Solvent control: microorganism grown in respective medium with addition of DMSO (1% final concentration).

Growth control: microorganism grown in respective medium without addition of DMSO and without test compound.

Positive control: microorganism grown in respective medium with addition of vancomycin (9.4 µM, 0.6 µM) in DMSO (1% final concentration).

Baseline value: medium and trioxacarcin 692 (**2**) in DMSO (1% final concentration) was incubated alongside in the 96 well plate and served for background subtraction.

In the light exposure condition, solvent controls, growth controls, positive controls, baseline values and test samples were equally subjected to irradiation.

Growth (OD_{600}) was measured after 7 h in a 96-well plate. Bars represent mean ± standard deviation (SD) of three independent replicates inoculated from the same preculture (n = 3). Statistical significance was determined by one-way ANOVA with Dunnett's post hoc test compared to 1% DMSO control. NS, not significant; $P < 0.05$ (*), $P < 0.01$ (**), $P < 0.001$ (***), $P < 0.0001$ (****). The lowest concentration tested (9.4 µM) did not result in complete loss of the activity. Therefore, the MIC value is below the tested concentration range.
